# QiShenYiQi Pills Attenuates Ischemia/Reperfusion-Induced Cardiac Microvascular Hyperpermeability Implicating Src/Caveolin-1 and RhoA/ROCK/MLC Signaling

**DOI:** 10.3389/fphys.2021.753761

**Published:** 2021-12-17

**Authors:** Chun-Shui Pan, Li Yan, Se-Qi Lin, Ke He, Yuan-Chen Cui, Yu-Ying Liu, Bai-He Hu, Xin Chang, Xin-Rong Zhao, Jing-Yu Fan, Jing-Yan Han

**Affiliations:** ^1^Tasly Microcirculation Research Center, Peking University Health Science Center, Beijing, China; ^2^Key Laboratory of Microcirculation, State Administration of Traditional Chinese Medicine of the People's Republic of China, Beijing, China; ^3^Key Laboratory of Stasis and Phlegm, State Administration of Traditional Chinese Medicine of the People's Republic of China, Beijing, China; ^4^Department of Integration of Chinese and Western Medicine, School of Basic Medical Sciences, Peking University, Beijing, China; ^5^Key Laboratory of Modern Preparation of Traditional Chinese Medicine, Ministry of Education, Jiangxi University of Traditional Chinese Medicine, Nanchang, China; ^6^State Key Laboratory of Core Technology in Innovative Chinese Medicine, Tianjin, China

**Keywords:** QSYQ, cardiac microvascular hyperpermeability, endothelial damage, cell-cell junctions, cytoskeleton

## Abstract

**Aims:** Coronary microvascular hyperpermeability is an important contributor to ischemia or reperfusion (I/R) injury. However, the effective strategy for this insult remains limited. This study aimed to explore the protective effect of the compound Chinese medicine QiShenYiQi Pills (QSYQ) against coronary microvascular hyperpermeability after cardiac I/R with focusing on the underlying mechanism.

**Methods and Results:** Male Sprague-Dawley rats under anesthesia were subjected to occlusion of left coronary anterior descending artery followed by reperfusion. QSYQ was administrated 90 min before ischemia initiation. Human cardiac microvascular endothelial cells (HCMECs) underwent hypoxia or reoxygenation (H/R) challenge with QSYQ administrated 1 h prior to hypoxia. QSYQ exhibited effects on attenuating microvascular damage and albumin leakage after I/R injury, showing a role in maintaining endothelial junctions, caveolae, and collagen in basement membrane (BM) of microvessels. Study using HCMECs disclosed that QSYQ protected endothelial barrier from impairment by H/R, attenuating the decline of respiratory chain complex I and ATP synthase, activation of Src/caveolin-1 and increase of RhoA/ROCK/p-MLC, MMP-9, and CTSS. PP2, a Src inhibitor, partially imitated the effect of QSYQ.

**Conclusions:** The QSYQ was able to prevent I/R-induced cardiac microvascular hyperpermeability *via* a mechanism involving Src/caveolin-1 and RhoA/ROCK/MLC signaling.

## New and Noteworthy

- QSYQ prevents I/R-induced cardiac microvascular hyperpermeability *in vivo* and *in vitro*.
- QSYQ interfered with Src/caveolin-1 and RhoA/ROCK signaling pathways in HCMECs exposed to H/R.

## Introduction

Timely and complete reperfusion after acute myocardial infarction is the most effective way of limiting infarct size and subsequent ventricular remodeling, which, however, provokes irreversible injury to the myocardium and the coronary circulation (Heusch, 2004; Yellon and Hausenloy, 2007; Heusch et al., 2014) leading to ischemia and reperfusion (I/R) injury with microvascular hyperpermeability as an initiator (Heusch and Gersh, 2017). Thus, development of strategies to protect the microvasculature from impairment by I/R is required (Yang et al., 2016; Shen et al., 2018).

The microvascular endothelium constitutes a physical barrier that regulates the exchange of molecules between blood and tissues (Pries and Kuebler, 2006). The transport of macromolecules across the microvascular endothelium is adjusted by caveolae within endothelial cells (transcellular pathway) and the junctions between adjacent endothelial cells (paracellular pathway) (Weis, 2008) including tight and adherent junctions (TJs and AJs) (Wallez and Huber, 2008). Src/caveolin-1 signaling is known to mediate the transcellular traffic, whereas RhoA/ROCK pathway is involved in modulation of intercellular junctions by imposing effect on the expression of respiratory chain complex to restore the impaired energy metabolism (Pries and Kuebler, 2006; Yan et al., 2021) and depolymerized F-actin cytoskeleton after I/R (Yan et al., 2018). NDUFA12 encodes one of the accessory subunits of complex I (Ostergaard et al., 2011) and is required for the formation of the extramembrane arm of human mitochondrial complex I (Rak and Rustin, 2014). YME1L1, an ATP-dependent metalloprotease, plays an important role in regulating mitochondrial morphology and function and maintaining complex I respiration activity (Stiburek et al., 2012; El-Hattab et al., 2018). ATP5D is known as the delta subunit of mitochondrial ATP synthase and could be regulated by RhoA/ROCK pathway. Besides, vascular BM contributes to microvascular barrier significantly, which consists of laminins, collagen IV isoforms, nidogens, and heparan sulfate proteoglycans (Katt et al., 2018). The collagen degradation is mostly mediated by matrix metalloproteinases (MMPs) including MMP-2 and MMP-9. Cathepsin S (CTSS) released from lysosome also has elastolytic and collagenolytic activities (Brown et al., 2020). Obviously, to safeguard the microvascular barrier, a measure with potential to target most, if not all, the signaling involved is needed.

QiShenYiQi Pills (QSYQ) is a traditional Chinese medicine composing of Astragalus membranaceus (Huangqi), Salvia miltiorrhiza (Danshen), and Panax notoginseng (Sanqi), which was approved for treating coronary heart disease and angina by the Chinese State Food and Drug Administration. The metabolite profiling and pharmacokinetics of herbal compounds following oral administration of QSYQ in rats have been reported (Zhang et al., 2012). Our laboratory revealed that QSYQ and its major effective ingredients, such as R1, Rb1, Rg1, DLA, and astragaloside IV, could alleviate infarct area, myocyte apoptosis, and cardiac dysfunction after I/R injury in rats (Lin et al., 2013; Tu et al., 2013; He et al., 2014; Yang et al., 2015; Cui et al., 2017; Li et al., 2018). It was also reported to cause a marked increase in myocardial capillary density and activation of endothelial growth factor in infarct heart (Zhang et al., 2010). However, the effect of QSYQ on cardiac microvascular hyperpermeability after I/R injury is still not clear. In this study, we explored whether QSYQ could reduce I/R-induced hyperpermeability of cardiac microvessels with focusing on the underlying mechanism.

## Materials and Methods

### Animals

Male Sprague-Dawley (SD) rats weighing 230–270 g were obtained from the Animal Center of Peking University (certificate number SCXK (Jing) 2006-0008). The rats were raised at a temperature of 22 ± 2°C and relative humidity 40 ± 5% under a 12-h light/dark cycle with access to standard diet and water *ad libitum*. The rats were fasted for 12 h before experiment while allowing to reach water freely. The investigations complied with the Guide of Peking University Animal Research Committee. All experimental procedures were approved by Peking University Biomedical Ethics Committee Experimental Animal Ethics Branch (LA2016314). Animals were randomly assigned to different groups.

### Drug

The QSYQ (batch number: 20120914) was obtained from Tasly Pharmaceutical Co. Ltd. (Tianjin, China), which was manufactured in accordance with the guidelines of Good Manufacturing Practice and Good Laboratory Practice.

### Cardiac I/R Model and Experiment Protocols

Animals were anesthetized with urethane (1.25 g/kg, i.m) and placed backside down, assisted with an animal breathing apparatus (ALC-V8; Shanghai Alcott Biotech Co., Shanghai, China) through a tracheal cannula inserted *via* mouth, which was set at the breathing ratio 1:1, the frequency 75 times/min, and tidal volume 12 ml/kg. The heart was exposed by thoracotomy, and the left anterior descending coronary artery was ligated with a 5/0 silk. The suture silk was released after 30 min, allowing reperfusion for 90 min. The animals in normal saline (NS) + Sham and QSYQ + Sham groups underwent the same procedure except for ligation of suture silk. Ninety min before ischemia, the animals in QSYQ pretreatment group were administrated through gavage with QSYQ in saline at a dose of 0.6 g/kg. The animals in NS + Sham group and NS + I/R group received equal volume of saline in the same way.

### Cell Culture and H/R Model

The primary human cardiac microvascular endothelial cells (HCMEC, ScienCell™ Research Laboratories, Carlsbad, California, USA) were cultured in a 5% CO_2_ incubator at 37°C with a culture medium containing 10% heat-inactivated fetal bovine serum (FBS, Gibco Laboratories, Grand Island, New York, USA), extracellular matrix (EMC, HyClone Laboratories, Logan, Utah, USA), and 100 units/mL penicillin or streptomycin (Gibco Laboratories, Grand Island, New York, USA). To establish hypoxia and reoxygenation (H/R) model, the cells were subjected to hypoxia (5% CO_2_ and 1% O_2_) by culturing in a microaerophilic system (Thermo Scientific, Waltham, MA) for 2 h with low glucose DMEM medium without FBS and then placed in the normoxic incubator for 3 h for reoxygenation with the normal culture medium. The control group cells were kept in the normal incubator. QSYQ (0.1 mg/ml) or PP2 (a Src inhibitor, 10 μM) was administrated 1 h ahead of H/R.

### Vascular Corrosion Casts of Rat Heart

After 90-min reperfusion, the right atrium was cut, blood was flushed out with 60 ml of PBS (pH 7.4, room temperature) containing heparin (0.2 ml, 1.000 USP u/ml), and 30 ml of low-viscosity resin (xinxingbairui, China) was infused into left ventricle (LV). Fifteen min later, resin-filled heart was removed and immersed in water for 30 min to complete resin polymerization. Heart was cut with razor blades and immersed alternatively in 20% NaOH and distilled water to get rid of tissue. This procedure was undertaken about 1 week for complete removal of tissue. The coronary vascular casts were rinsed thoroughly in distilled water and air-dried and carefully mounted on an aluminum stub using double-stick carbon tape. Samples were then coated with gold in argon gas at 25 mA for 2 min in a sputter coater and examined in a field emission scanning electron microscope (SEM) (JSM-5600LV, JEOL, Tokyo, Japan).

### Ultrastructure Examination

Rat heart was perfused for 40 min with 4% paraformaldehyde and 2% glutaraldehyde (Ted Pella, Redding, CA, USA) in 0.1 mol/L phosphate buffer at a speed of 3 ml/min and then removed. Myocardial tissue was collected from the surrounding region of infarct in LV and cut into blocks <1 mm^3^. The tissue blocks were fixed overnight at 4°C with 3% glutaraldehyde, washed 3 times with 0.1 mol/L phosphate-buffered solution (PBS), and then postfixed with 1% osmium tetroxide for 2 h. The ultrathin sections were prepared as routine and observed and photographed with a transmission electron microscope (JEM 1400 plus, JEOL, Tokyo, Japan).

### Fluorescein Isothiocyanate Albumin Leakage From Coronary Venules

The animals were intravenously injected with FITC-bovine serum albumin (BSA) (SIGMA, USA) at 50 mg/kg after reperfusion. After 10 min of baseline observation, fluorescence intensity of FITC-BSA was recorded (excitation, 455 nm; emission, 530 nm) using a silicon-intensified target camera (C-2400-08; Hamamatsu, Shizuoka, Japan) and a DVD videocassette recorder (DVR-R25; Malata, Xiamen, China). The fluorescent intensity within the venules (Iv) and in the perivenular interstitium (Ip) was measured with Image-Pro Plus 5.0 software (Bethesda, MD). Albumin leakage was evaluated by Ip/Iv.

### Cardiovascular Collagen Stain

To determine the collagen in vascular BM, the heart tissues were immersion-fixed in 4% paraformaldehyde, embedded in paraffin, and processed for 5-μm slices. Deparaffinized and rehydrated slices were stained with Sirius Red, examined using a light microscope (Olympus, Tokyo, Japan), and photographed with a digital camera.

### Immunofluorescence Staining and Confocal Microscopy

Heart paraffin sections were permeabilized with 0.3% TritonX-100, blocked with goat serum, and incubated with primary antibodies diluted in PBS overnight at 4°C. The primary antibodies applied were as follows: mouse anticlaudin-5 (1:100, Invitrogen, Camarillo, CA, USA), rabbit anti-vWF (1:100, Millipore, Temecula, CA, USA), and rabbit anticollage-IV (1:50, Invitrogen, Camarillo, CA, USA). After rinsing with PBS, heart sections were then incubated with Dylight 488-labeled goat antirabbit IgG (KPL, Gaithersburg, MD, USA) and Dylight 549-labeled goat antimouse IgG (KPL, Gaithersburg, MD, USA) for 2 h at room temperature.

The 4% paraformaldehyde-fixed HCMECs were permeabilized with 0.3% TritonX-100 and stained with rhodamine phalloidin (Invitrogen, Carlsbad, USA) for F-actin at 37°C for 2 h. Hoechst 33342 (Molecular Probes) was applied to stain all the nuclei. The results were examined by a laser scanning confocal microscope (TCS SP5, Mannheim, Germany).

### Cell Viability Assay

Cell viability was assessed by a Cell Counting Kit-8 (CCK-8; Dojindo Molecular Technologies, Gaithersburg, MD, USA) according to the manufacturer's instructions. Briefly, after treatment of cells, the CCK-8 solution was added to the culture medium and incubated at 37°C for 1 h. The absorbance of each well was measured at 450 nm using microplate reader (Multiskan MK3, Thermo, USA). Cell viability was quantified using average absorbance × 100%.

### ELISA for Content of ATP and AMP and the Activity of YME1L1, MMP2, MMP9, and CTSS

Human cardiac microvascular endothelial cells were harvested and mixed with 100 μL RIPA lysis buffer. The whole protein of cells was extracted with a protein extraction kit (Applygen, Beijing, China). In brief, the mixture was homogenized, incubated on ice for 30 min, and centrifuged at 20,000 g, 4°C, for 10 min. The resultant supernatant was taken as whole protein. The content of ATP and AMP and the activities of MMP2, MMP9, CTSS, and YME1L1 were assessed by respective ELISA kit (Huanya Biomedicine Technology, Beijing, Andygene, Beijing, China) and detected by microplate reader (Multiskan MK3, Thermo, USA), according to the manufacturer's instruction.

### Activities of Mitochondrial Respiratory Chain Complex I and ATP Synthase

Human cardiac microvascular endothelial cells were harvested and then pipetted up and down to disperse the cells. The activity of mitochondrial respiratory chain complex I and ATP synthase was measured using respective ELISA kit (Abcam, Cambridge, United Kingdom). Briefly, samples were homogenized, pelleted and adjusted to 5.5 mg/ml, subjected to detergent extraction and centrifugation for 20 min at 16,000 g (for complex I), or 20,000 rpm (for ATP synthase), and followed by loading supernatant on plate and incubation for 3 h. Following washing for 3 times, optical density was measured after addition of assay solution. The activity was expressed as the change in absorbance at 340 nm (in optical density units/min).

### Endothelial Cell Monolayer Permeability Assay

The endothelial monolayer permeability to FITC-conjugated dextran (FITC-dextran; Sigma-Aldrich Chemicals, St. Louis, MO, USA) was assessed by using transwell permeable membranes (24-well cell culture inserts) with 0.4-μm pore size (Costar, Corning, NY, USA). HCMECs (2 × 10^5^) were seeded on gelatin-coated transwell filters and allowed to grow for 2 days in high glucose DMEM medium with FBS. The concentration of FITC-dextran transferred to the lower chamber in different conditions was determined with excitation and emission wavelengths of 492 and 520 nm, respectively, using the microplate reader (Multiskan MK3, Thermo, USA).

### Western Blot

The whole protein was extracted as described above. The concentration of total protein of each sample was determined two times with a BCA protein assay kit (Applygen Technologies, Beijing, China), according the to the manufacture's instruction, taking the average as the concentration. The protein was separated on 8 or 10% SDS-PAGE and transferred to polyvinylidene difluoride membrane. The membrane was incubated overnight at 4°C with antibodies, respectively, against p-Src/Src (1:1000, CST, Vermont, USA), p-caveolin-1/caveolin-1 (1:1000, CST, Vermont, USA), claudin-5 (1:1000, Invitrogen, California, USA), occludin (1:1000, Invitrogen, California, USA), VE-cadherin (1:1000, Abcam, Cambridge, UK), ZO-1 (1:1000, Invitrogen, California, USA), ATP5D (1:200, Santa Cruz, California, USA), p-MLC/MLC (1:1000, CST, Vermont, USA), ROCK (1:1000, Abcam, Cambridge, UK), RhoA (1:1000, Abcam, Cambridge, UK), YME1L1 (1:1000, Abcam, Cambridge, UK), NDUFA12 (1:1000, Abcam, Cambridge, UK), MMP-2 (1:1000, Abcam, Cambridge, UK), MMP-9 (1:1000, Abcam, Cambridge, UK), CTSS (1:1000, Abcam, Cambridge, UK), and GAPDH (1:1000, CST, Vermont, USA). GAPDH was used as a loading control. Enhanced chemiluminescence detection kit (Applygen Technologies, Beijing, China) was applied for revealing the bands. Band intensities were quantified by densitometry and presented as mean area density using Quantity One Image Analyzer software (Bio-Rad; Richmond, CA, USA).

### Statistical Analysis

All parameters were expressed as mean ± SE. Statistical analysis was performed using one-way ANOVA followed by Newman–Keuls test or using two-way ANOVA followed by Bonferroni for multiple comparisons. Data were analyzed using GraphPad Prism 7 software (GraphPad software Inc., USA). A *p* < 0.05 was considered to be statistically significant.

## Results

### QSYQ Protected Vascular Architecture From Disorder in Rat Heart and Ameliorated Albumin Leakage From Coronary Venules

**Figure 1:**
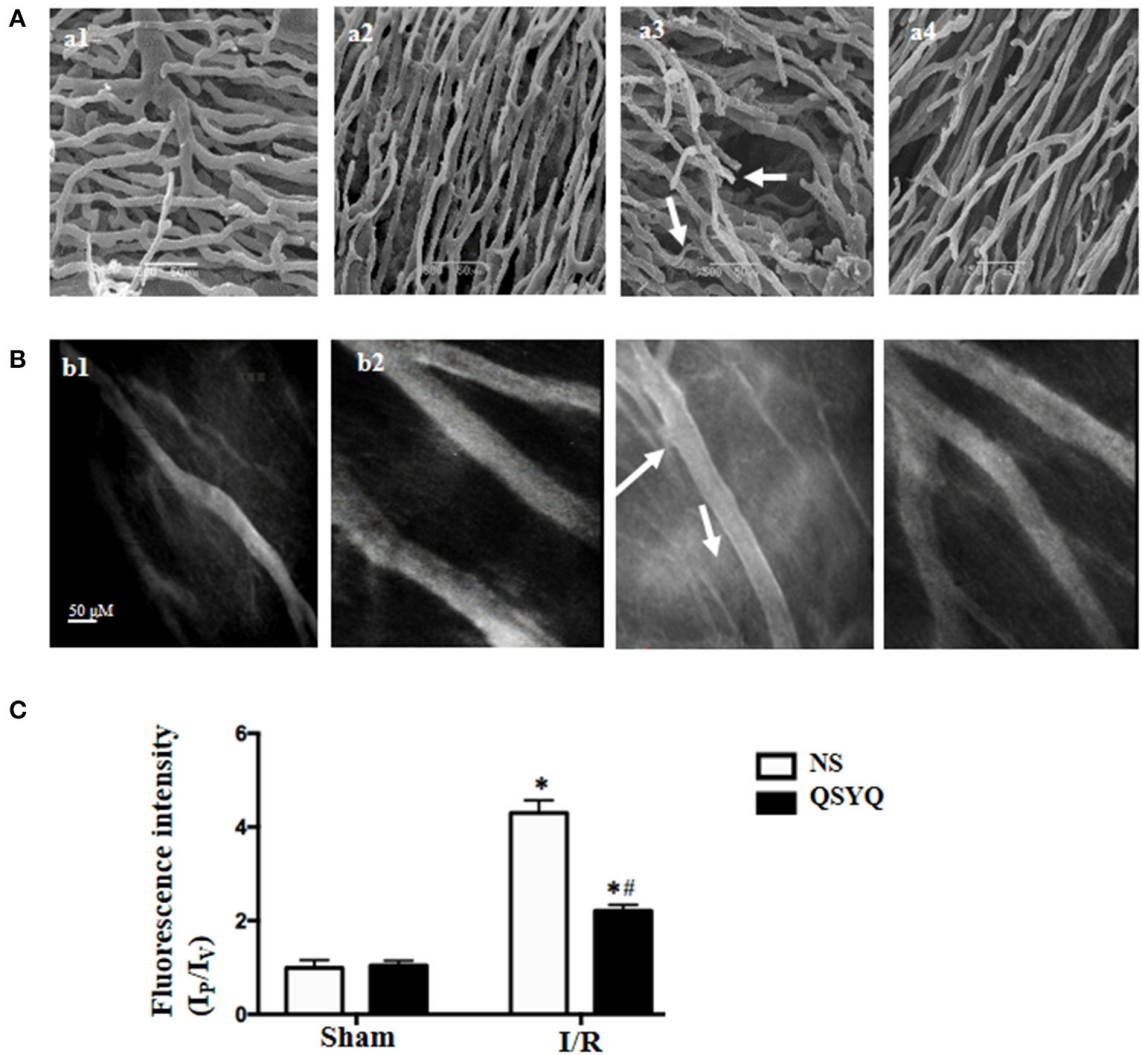
Effect of pretreatment with QSYQ on the vascular corrosion casts and coronary venular albumin leakage of rat heart. **(A)** The representative scanning electron micrographs of myocardial vascular corrosion casts in different groups. Arrows: distorted and narrowed vessel segments. a1: NS + Sham group; a2: QSYQ + Sham group; a3: NS+I/R group; a4: QSYQ+I/R group. Bar = 50 μ m. **(B)** Representative images of FITC-albumin leakage from coronary venules in different groups. b1: NS + Sham group; b2: QSYQ + Sham group; b3: NS+I/R group; b4: QSYQ+I/R group. Bar = 50 μ m. **(C)** Statistical results of albumin leakage expressed as ratio of fluorescence intensity in the Ip to that Iv. Results are presented as mean ± SEM (*n* = 6). *p < 0.05 vs. NS + Sham group, ^#^*p* < 0.05 vs. NS + I/R group. Statistical analysis was performed using two-way ANOVA followed by Bonferroni for multiple comparisons.

Illustrated in Figure 1 are the representative SEM images of corrosion casting vasculatures in hearts from different groups. Capillaries in NS + Sham group (Figure 1A,a1) and QSYQ + Sham group (Figure 1A,a2) exhibited a rather regular orientation with uniform diameter. In contrast, capillaries in NS+I/R heart manifested distorted and narrowed vessel segments (Figure 1A,a3), which most likely resulted from the pressure imposed by interstice edema and accounts for the elevated resistance and slowed blood flow and indicates microcirculation disturbance. QSYQ pretreatment ameliorated vasculature disorders induced by I/R (Figure 1A,a4).

**Figure 2:**
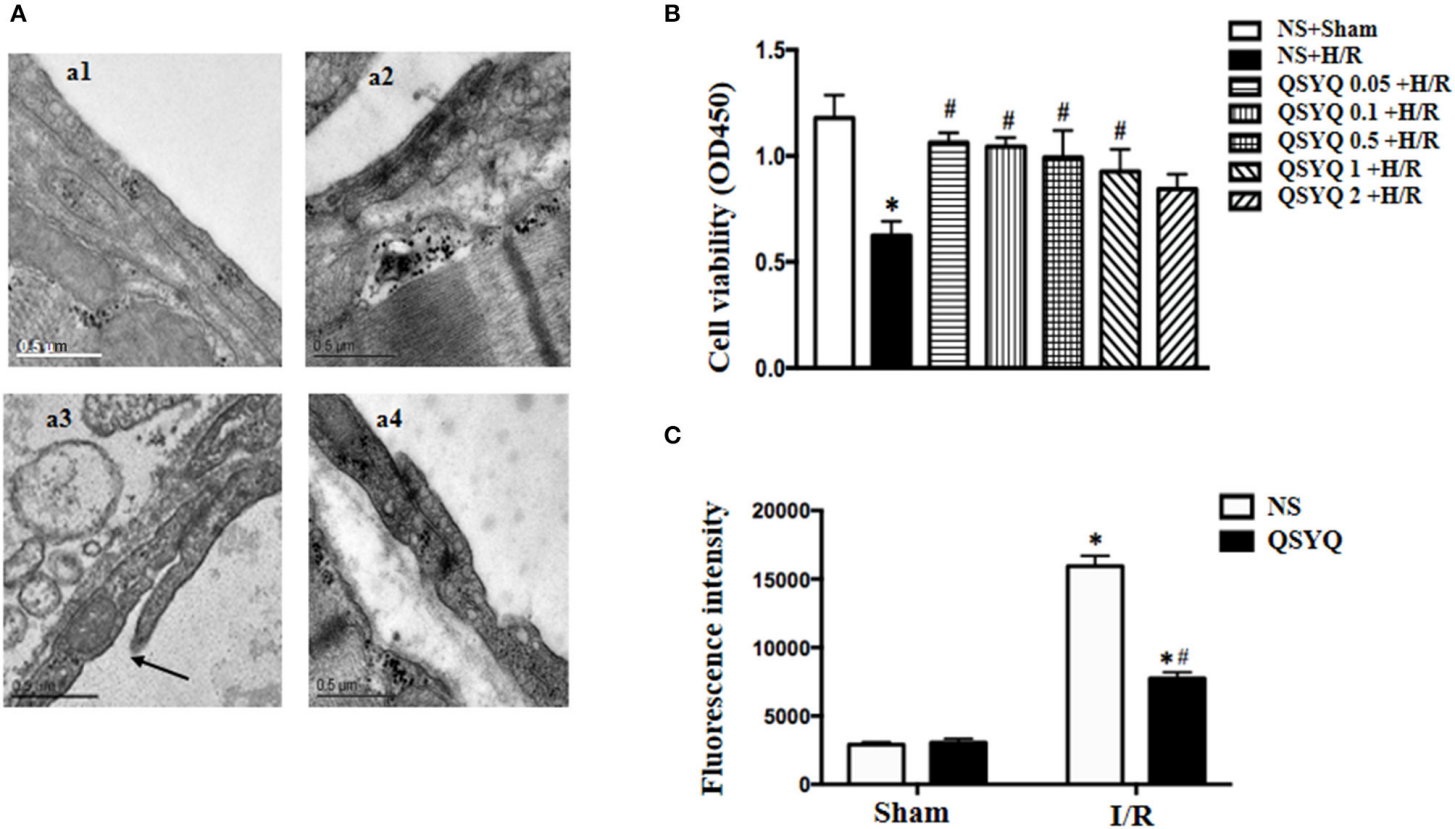
Effect of pretreatment with QSYQ on cardiac microstructure in rats and viability and endothelial barrier integrity of HCMECs. **(A)** Representative electron micrographs of cardiac vascular endothelium in different groups. a1: NS + Sham group; a2: QSYQ + Sham group; a3: NS+I/R group; a4: QSYQ+I/R group. Arrow: intercellular gap. Bar = 0.5 μ m. **(B)** Viability of HCMECs challenged by H/R with or without various dosages of QSYQ. Statistical analysis was performed using one-way ANOVA followed by Newman–Keuls test. **(C)** Effect of QSYQ on hyperpermeability of HCMEC monolayer challenged by H/R. The permeability was expressed as the optical density at 492 nm of FITC-dextran in the lower chamber. Data are expressed as means ± SEM (*n* = 6). *p < 0.05 vs. NS + Sham group or control group, ^#^*p* < 0.05 vs. NS + I/R group. Statistical analysis was performed using two-way ANOVA followed by Bonferroni for multiple comparisons.

The observation of albumin leakage from coronary venules and ultrastructure of microvessels confirmed the results form corrosion casting and SEM. Figure 1B presented the representative images of FITC-labeled albumin leakage from coronary venules in different groups with statistic result depicting in Figure 1C. Compared with NS + Sham and QSYQ + Sham groups (Figure 1B,b1,b2), I/R evoked an obvious FITC-labeled albumin leakage from coronary venules (Figure 1B,b3), which was significantly prevented by pretreatment with QSYQ (Figure 1B,b4). Vascular permeability is regulated by endothelial cells, in which intercellular junctions are the major determinants. As expected, electron microscopy revealed an obvious intercellular gap in vascular endothelial cells in NS+I/R group (Figure 2A,a3) as compared to NS + Sham and QSYQ + Sham groups (Figure 2A,a1,a2), suggesting involvement of endothelial cell junctions in microvascular hyperpermeability after I/R, which was alleviated by pretreatment with QSYQ (Figure 2A,a4).

### QSYQ Protected HCMECs From Damage and Ameliorated FITC-Dextran Moving Across HCMECs Monolayer After H/R

To assay the role of QSYQ in H/R-deteriorated viability of endothelial cells, HCMECs were examined in different conditions using the CCK-8 assay. There was no statistics difference between NS + Sham and QSYQ + Sham groups (1.18 ± 0.11 vs. 1.11 ± 0.06). Compared with NS + Sham, H/R evoked an obvious decrease in cell viability, which was significantly prevented by pretreatment with QSYQ in concentrations ranging from 0.05 to 1 mg/ml, with lower concentrations appearing more effective. The dose of 0.1 mg/ml QSYQ was applied in all subsequent *in vitro* experiments (Figure 2B).

The permeability of HCMECs monolayer for FITC-dextran was assessed in various conditions by culturing the cells in a 24-well transwell chamber. Compared with NS + Sham and QSYQ + Sham groups, a significant increase of FITC-dextran was observed in the lower chamber in H/R group, suggesting an increased permeability of the HCMECs monolayer (Figure 2C). Apparently, pretreatment with 0.1 mg/ml of QSYQ showed a reduced fluorescence intensity of liquid in the lower chamber as compared to H/R, suggesting the ameliorating effect of QSYQ on the endothelial barrier.

### QSYQ Blocked the Phosphorylation of Src and Caveolin-1 in HCMECs Induced by H/R

**Figure 3:**
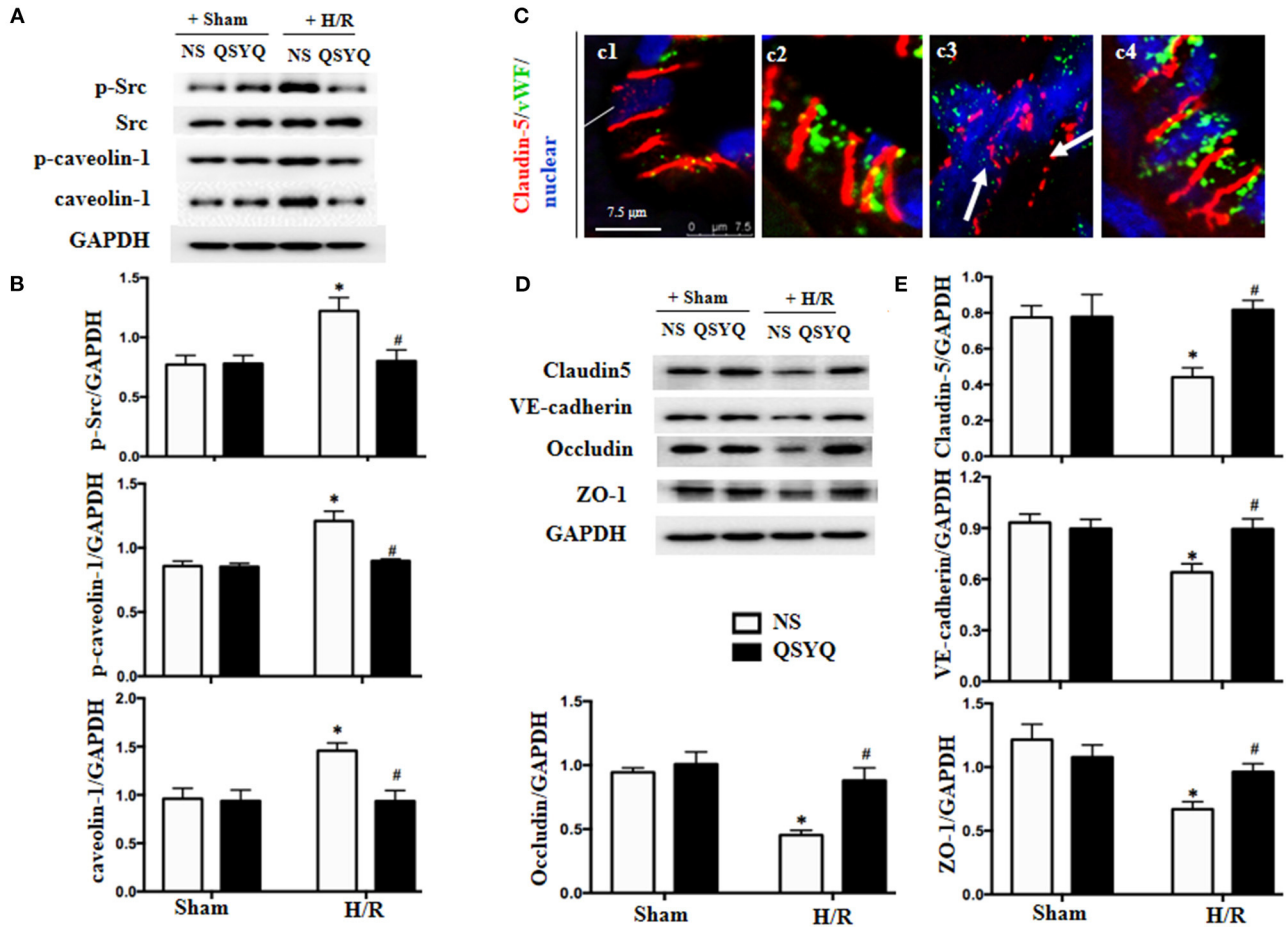
Effect of QSYQ pretreatment on intercellular junction proteins and Src/caveolin-1 in HCMECs after H/R. **(A)** Representative western blot bands of Src, p-Src, caveolin-1, and p-caveolin-1 in various groups of HCMECs. GAPDH was used as a loading control. **(B)** Relative densitometric values of p-Src, caveolin-1, and p-caveolin-1 corrected for GAPDH. Data are expressed as means ± SEM (*n* = 6). *p < 0.05 vs. NS + control group, ^#^*p* < 0.05 vs. NS + H/R group. Statistical analysis was performed using two-way ANOVA followed by Bonferroni for multiple comparisons. **(C)** Representative immunofluorescence confocal images of claudin-5 in rat heart vessels. Arrows: discontinuous distribution of claudin-5. c1: NS + Sham group; c2: QSYQ + Sham group; c3: NS+I/R group; c4: QSYQ+I/R group. Claudin-5 (red) localized between the endothelial cells with marker vWF (green). Bar = 7.5 μm. **(D,E)** Representative western blot bands **(D)** and relative densitometric values **(E)** of claudin-5, ZO-1, occludin, and VE-cadherin in HCMECs in different groups. GAPDH was used as a loading control. Data are expressed as means ± SEM (*n* = 6). *p < 0.05 vs. NS + control group, ^#^*p* < 0.05 vs. NS + H/R group. Statistical analysis was performed using two-way ANOVA followed by Bonferroni for multiple comparisons.

Src has been known as an upstream signaling pathway to regulate endothelial permeability *via* modulation of caveolin activity. To investigate the role of Src in the protective effect of QSYQ on microvascular endothelium barrier after I/R injury, we examined the expression and activation of Src and caveolin-1 in HCMECs by western blot. As shown in Figures 3A,B, H/R increased the phosphorylated Src obviously while had no effect on Src expression. On the other hand, H/R increased both the expression and phosphorylation of caveolin-1. The change in phosphorylation of Src and caveolin-1 and the expression of caveolin-1 in response to H/R were all alleviated by pretreatment with QSYQ significantly. This result suggests an involvement of Src/caveolin-1 signaling in QSYQ protective effect on endothelial hyperpermeability after H/R.

### QSYQ Alleviated Degradation of Junction Proteins in I/R-Induced Rat Heart and HCMECs Induced by H/R

In view of the critical role of intercellular junctions in maintaining vascular integrity, the expression and distribution of vascular endothelial TJ protein claudin-5 were first examined by confocal microscopy, with representative images of different groups displaying in Figure 3C. Apparently, claudin-5 localized between endothelial cells as continuous lines in Sham groups (Figure 3C,c1–c2). Reperfusion for 90 min disrupted the continuously distributed claudin-5, resulting in an intermittent and weakened staining, indicating degradation of claudin-5 in response to I/R (Figure 3C,c3). Interestingly, this degradation was reversed remarkably by QSYQ pretreatment (Figure 3C,c4).

The role of QSYQ in protection of endothelial junction proteins was further verified by *in vitro* study using HCMECs H/R model. Western blot showed that, as compared to NS + Sham and QSYQ + Sham groups, H/R exposure provoked a decrease in the expressions of TJ proteins claudin-5, occludin and zona occludens-1 protein (ZO-1) and the AJ protein VE-cadherin (Figures 3D,E), suggesting a damaged endothelial cell barrier induced by H/R. Pretreatment with QSYQ protected the changes in junction proteins after H/R significantly. This result suggests an implication of modulating junction proteins in QSYQ protective effect on endothelial hyperpermeability after H/R.

### QSYQ Attenuated Actin Cytoskeleton Abnormality and ATP/AMP Ratio Decrease of HCMECs After H/R

**Figure 4:**
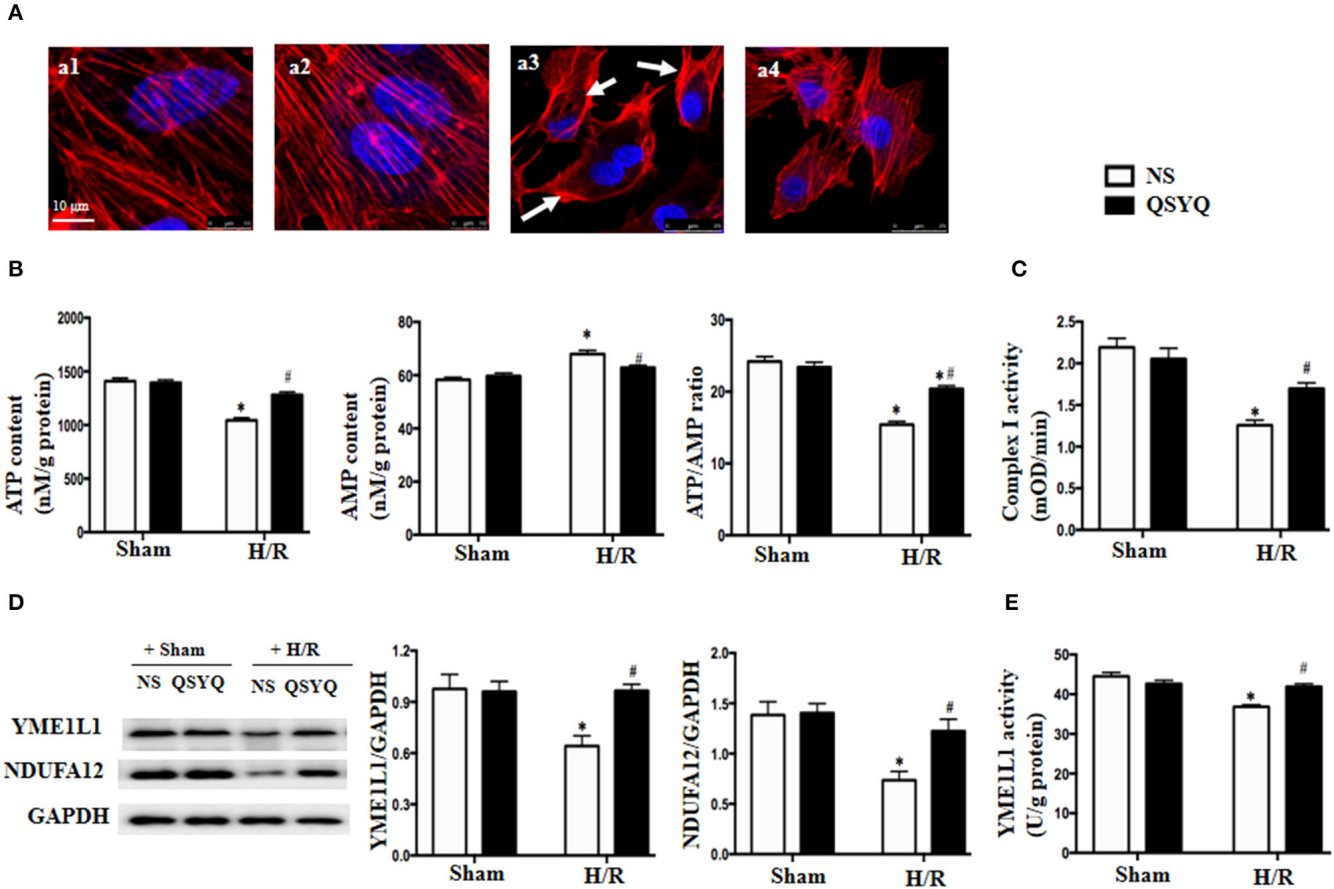
Effect of pretreatment with QSYQ on cytoskeleton, ATP/AMP content, the expression of YME1L1 and NUDFA12 and complex I activity after H/R. **(A)** The F-actin cytoskeleton in HCMECs (red). Arrow: F-actin cytoskeleton that is concentrated at the periphery of cell and disintegrated. Bar = 10 μm. a1: NS + Sham group; a2: QSYQ + Sham group; a3: NS + H/R group; a4: QSYQ + H/R group. **(B)** Quantitative measurement of ATP and AMP by ELISA in HCMECs. **(C)** Quantitative measurement of complex I activity by ELISA in HCMECs. **(D)** Representative western blot bands and relative densitometric values of YME1L1 and NUDFA12 in HCMECs in different groups. GAPDH was used as a loading control. Data are expressed as means ± SEM (*n* = 6). *p < 0.05 vs. NS + control group, ^#^*p* < 0.05 vs. NS + H/R group. **(E)** Quantitative measurement of YEM1L1 activity by ELISA in HCMECs. Values are means ± SEM (*n* = 6). *p < 0.05 vs. NS + control group, ^#^*p* < 0.05 vs. NS + H/R group. Statistical analysis was performed using two-way ANOVA followed by Bonferroni for multiple comparisons.

Actin is known to play a crucial role in maintaining endothelium barrier integrity. We thus assessed the actin cytoskeleton of HCMECs by rhodamine-phalloidine staining in different conditions (Figure 4A). As compared to NS + Sham and QSYQ + Sham cells (a1, a2), H/R led to a dramatic change in F-actin structure and distribution in HCMECs, with most of the actin stress fibers localizing as bundles in the periphery of the cells, whereas those in the cytoplasm becoming disintegrated (a3). Obviously, the H/R-elicited change in actin stress fibers was significantly attenuated by pretreatment with QSYQ (a4).

The ATP is required for F-actin polymerization, and disarrangement of F-actin after H/R implied a depletion of ATP. We tested this deduction in HCMECs by ELISA. The result in Figure 4B shows that as compared to NS + Sham and QSYQ + Sham groups, ATP level in NS+H/R group significantly decreased, whereas AMP level increased, suggesting an impairment of energy metabolism after H/R. Interestingly, alteration in ATP and AMP levels caused by H/R was prevented by pretreatment with QSYQ.

### QSYQ Protected the Activity of Complex I and ATP Synthase From Decline During H/R

**Figure 5:**
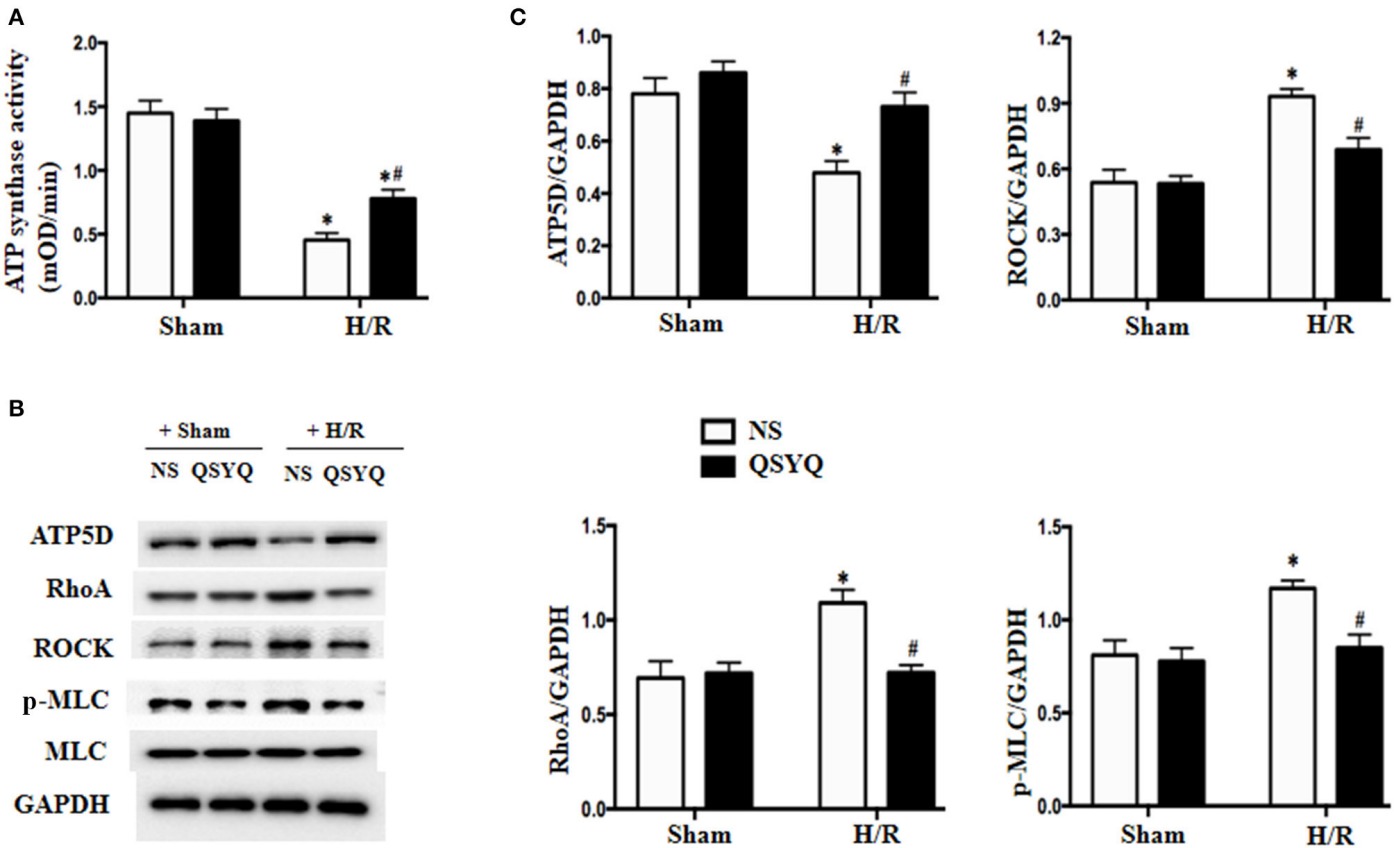
Effect of QSYQ on the activity of ATP synthase and the expression of RhoA, ROCK, and p-MLC in HCMECs after H/R. **(A)** Quantitative measurement of complex I activity by ELISA in HCMECs. **(B)** Representative western blot bands of ATP5D, RhoA, ROCK, MLC, and p-MLC in HCMECs. GAPDH was used as a loading control. **(C)** Relative densitometric values of ATP5D, RhoA, ROCK, and p-MLC corrected for GAPDH. Data are expressed as means ± SEM (*n* = 6). *p < 0.05 vs. NS + control group, ^#^*p* < 0.05 vs. NS + H/R group. Statistical analysis was performed using two-way ANOVA followed by Bonferroni for multiple comparisons.

The majority of ATP in cells is generated by electron transport chain complexes in mitochondria. To assay the role of QSYQ on ATP production, we detected the activity of complex I and ATP synthase in HCMECs. The results showed that the activities of complex I and ATP synthase were remarkably reduced in NS+H/R group as compared to NS + Sham and QSYQ + Sham groups, which were ameliorated by pretreatment with QSYQ (Figures 4C, 5A).

We next detected changes in NDUFA12 and YME1L1, two proteins that are important for the activity of mitochondrial complex I, in HCMECs under H/R by using western blot. The result showed that H/R challenge elicited a significant decline in the expression of both NDUFA12 and YME1L1 (Figure 4D) and also the activity of YME1L1 (Figure 4E), which were ameliorated by pretreatment with QSYQ (Figures 4D,E). This result suggests an implication of NDUFA12 and YME1L1 in the protective effect of QSYQ on the activity of complex I.

The ATP5D is the delta subunit of mitochondrial ATP synthase. Result of western blot revealed that compared with NS + Sham and QSYQ + Sham groups, ATP5D level was remarkably reduced in NS+H/R group, which was reversed by pretreatment with QSYQ (Figures 5B,C).

### RhoA/ROCK Signaling Pathway Is Involved in the Protected Effect of QSYQ on HCMECs During H/R

The RhoA/ROCK has been known to play an important role in endothelial barrier dysfunction, whereas MLC is one of the targets of ROCK. We thus tested the likely involvement of RhoA/ROCK in the mechanism of QSYQ effect. Western blot in Figures 5B,C showed that H/R significantly elevated the expressions of RhoA and ROCK and promoted myosin light chain (MLC) phosphorylation in HCMECs. QSYQ lessened RhoA/ROCK/p-MLC increase induced by H/R, highlighting an involvement of the RhoA/ROCK/MLC signaling in the beneficial role of QSYQ observed.

### QSYQ Decreased the Expressions and Activities of MMP-2, MMP-9, and CTSS in HCMECs and Maintained the Vascular BM Integrity

**Figure 6:**
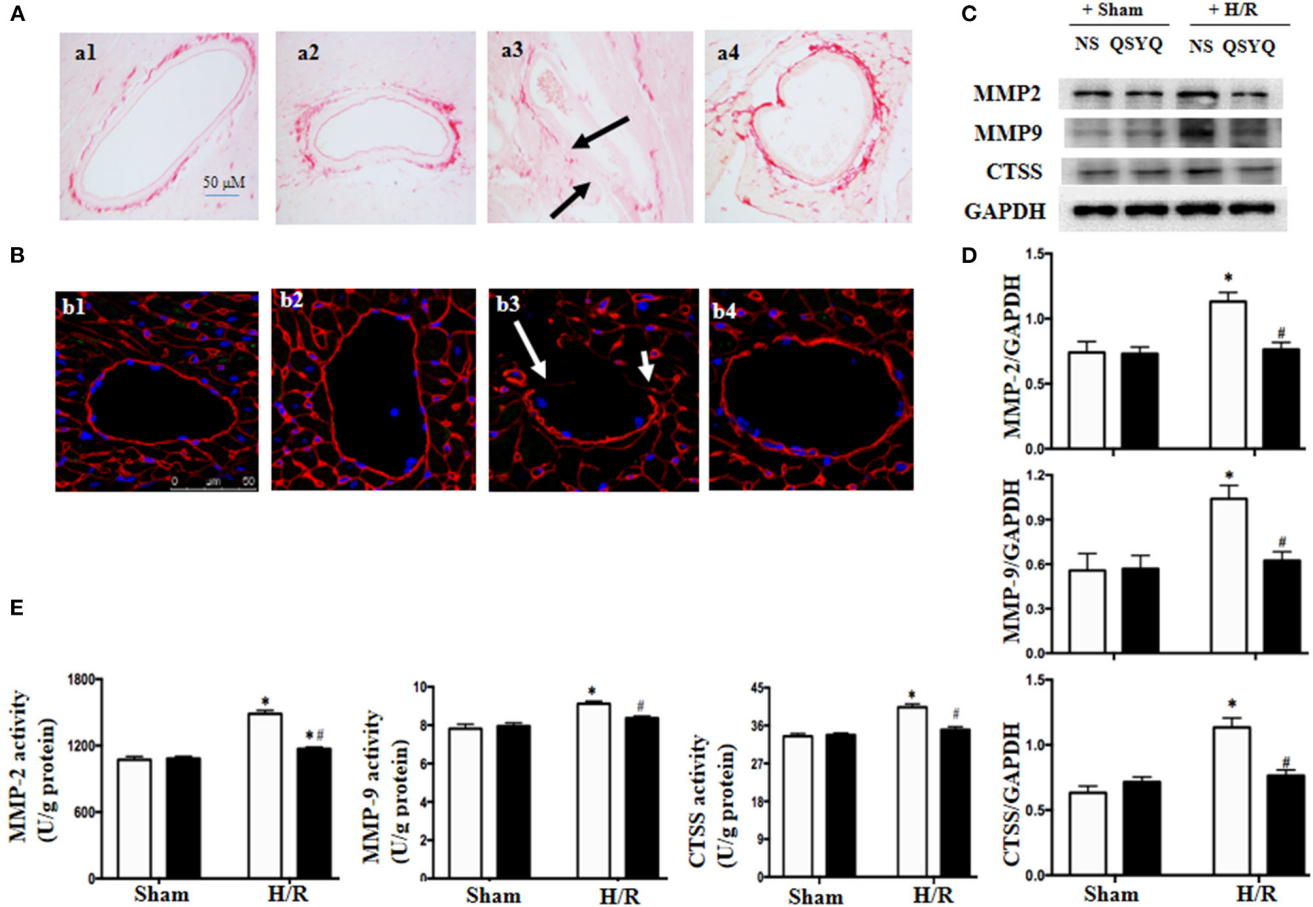
Effect of pretreatment with QSYQ on maintaining the vascular BM integrity and related proteins. **(A)** Collagen was stained by Sirius Red in different groups to show the collagen deposition in cardiac microvascular BM of rat. Arrows: decreased area of collagen deposition in BM. a1: NS + Sham group; a2: QSYQ + Sham group; a3: NS + I/R group; a4: QSYQ + I/R group. Bar = 50 μm. **(B)** Immunofluorescence confocal images of collage-IV (red) in coronary venules of rat in each group. Arrows: discontinuous distribution of collagen IV. b1: NS + Sham group; b2: QSYQ + Sham group; b3: NS + I/R group; b4: QSYQ + I/R group. Bars = 50 μm. **(C,D)** Representative western blot bands **(C)** and relative densitometric values **(D)** of MMP-2, MMP-9, and CTSS in HCMECs in different groups. GAPDH was used as a loading control. **(E)** The activity of MMP-2, MMP-9, and CTSS tested by ELISA in different groups of HCMECs. Data are expressed as means ± SEM (*n* = 6). *p < 0.05 vs. NS + control group, ^#^*p* < 0.05 vs. NS + H/R group. Statistical analysis was performed using two-way ANOVA followed by Bonferroni for multiple comparisons.

Basement membrane is an essential part of vascular endothelium barrier, which contains collagen as a major component. We thus assessed the status of collagen deposition in microvascular wall to explore the role of QSYQ in maintaining BM integrity. Displayed in Figure 6A are the Sirius Red-stained cardiac microvessels from different groups, which shows that the area of collagen deposition in BM decreased significantly after I/R (a3) compared with Sham groups (a1, a2). In contrast, treatments with QSYQ enhanced the collagen deposition in the vascular walls after I/R (a4). This result was confirmed by immunofluorescence staining of collagen IV in cardiac vascular wall as shown in Figure 6B, wherein a discontinuous distribution of collagen IV was observed in NS+I/R group (b3), which is in striking contrast to Sham groups (b1, b2), indicating degradation of collage-IV in response to I/R. Interestingly, this degradation was restored by pretreatment with QSYQ (b4).

The collagen degradation is mostly mediated by MMPs including MMP-2 and MMP-9, and also by CTSS, an enzyme released from lysosomes having elastolytic and collagenolytic activities. We next examined the expression and activities of MMP-2, MMP-9, and CTSS in HCMECs from various groups by western blot and ELISA, respectively. The results showed that H/R led to an increase in expressions (Figures 6C,D) and activities (Figure 6E) of MMP-2, MMP-9, and CTSS, which was in consonance with collagen degradation found in cardiac vascular wall after I/R. Intriguingly, the alterations of MMP-2, MMP-9, and CTSS were relieved by pretreatment with QSYQ (Figures 6C–E).

### PP2, Inhibitor of Src, Had an Effect Similar to QSYQ on HCMECs Exposed to H/R

**Figure 7:**
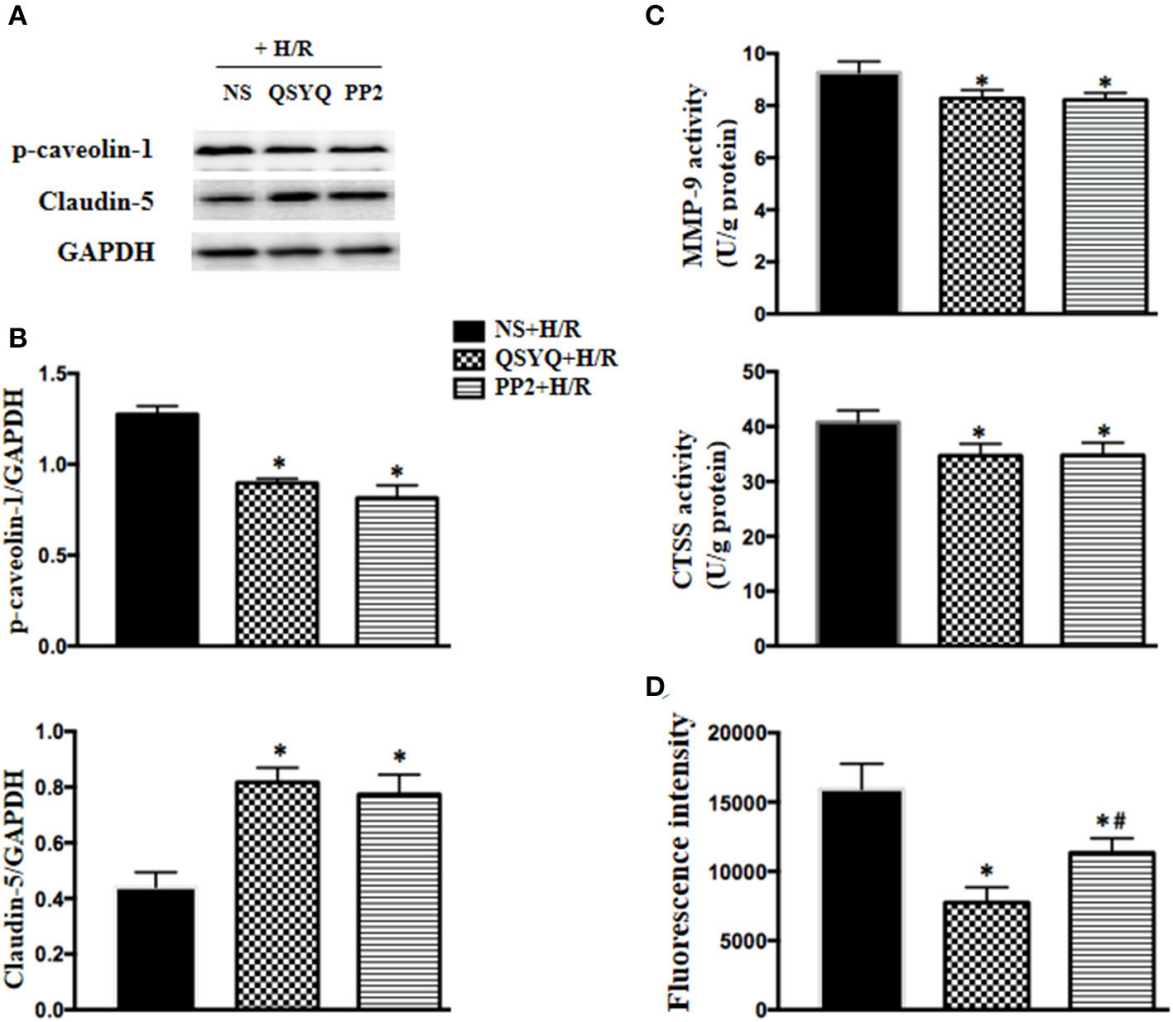
Effect of PP2 on the expression of claudin-5 and p-caveolin-1, the activity of MMP-9 and CTSS, and hyperpermeability of HCMECs monolayer exposed to H/R. **(A)** Representative western blot bands **(A)** and relative densitometric values **(B)** of p-caveolin-1 and claudin-5 in HCMECs in different groups. GAPDH was used as a loading control. **(C)** Quantitative measurement of MMP-9 and CTSS activity by ELISA in HCMECs. **(D)** Effect of QSYQ on hyperpermeability of HCMEC monolayer induced by H/R. The permeability was expressed as the optical density of FITC-dextran at 492 nm in the lower chamber. Data are expressed as means ± SEM (*n* = 6). *p < 0.05 vs. NS + Sham group or control group, ^#^*p* < 0.05 vs. NS + I/R group. Statistical analysis was performed using one-way ANOVA followed by Newman–Keuls test.

Caveolae-mediated transendothelial traffic contributes to endothelium permeability, which is regulated by Src. To explore whether Src/caveolae pathway is implicated in the mechanism whereby QSYQ takes effect, we treated HCMECs with PP2, a Src inhibitor, 1 h before H/R. The results showed that PP2 restored the level of p-caveolin-1 as QSYQ did (Figures 7A,B), demonstrating the role of Src in the mechanism for QSYQ mediating vascular permeability *via* transendothelial pathway. Furthermore, akin to QSYQ, PP2 restored the expression of claudin-5 and inhibited the activity of CTSS and MMP-9 (Figure 7C), the two enzymes known to exhibit activity to hydrolyze both BM proteins and junction proteins. Finally, PP2 protected H/R-caused hyperpermeability of HCMEC monolayer to FITC-dextran (Figure 7D), similar to QSYQ, suggesting involvement of Src/caveolae signal pathway in the effect of QSYQ on ameliorating microvascular hyperpermeability.

## Discussion

This study showed that reperfusion following coronary occlusion impaired rat cardiac microvessels manifesting abnormal vascular morphology, increased albumin leakage from venules, concurrent with a disconnection of the junctions between adjacent endothelial cells in cardiac capillaries, a disarranged collagen deposition and a downregulated expression of collagen IV in vascular wall *in vivo*, all these alterations were ameliorated by pretreatment with QSYQ. Study using H/R-challenged HCMECs revealed the mechanism for QSYQ to protect cardiac microvascular barrier involving regulation of paracellular pathway and transcellular pathway and integrity of vascular BM, a process implicating RhoA/ROCK/MLC and Src/caveolae signaling and restoration of energy metabolism.

Coronary microvascular dysfunction occurs in up to half of patients submitted to apparently successful primary percutaneous transluminal coronary intervention. Microvascular hyperpermeability after I/R results in no-reflow and myocardium tissue edema which limits tissue reoxygenation and provokes cardiac myocyte apoptosis (Kottke and Walters, 2016), aggravating ischemia-induced myocardium injury. Thus, development of approach to deal with I/R-elicited cardiac microvascular hyperpermeability has gained increasing attention. However, the so far proposed approaches prove to be either controversial (Zhao et al., 2006; Selker et al., 2012) in efficiency or inadequate (Erlinge et al., 2014; Montalescot et al., 2014; Korayem et al., 2017). In this work, we found that QSYQ ameliorated cardiac microvascular morphology and endothelial hyperpermeability induced by I/R, indicating QSYQ as a promising option for such a purpose.

Microvascular barrier is determined by TJs and AJs between adjacent endothelial cells, the number and function of caveolae in endothelial cell, and the integrity of BM under beneath the endothelial cell layer, which are regulated by a variety of signaling pathways. Besides, the actin filaments in the vascular endothelial cells contribute to keeping microvascular barrier integrity as well, which offer a support from inside for the intercellular junctions *via* ZO-1 (Alluri et al., 2016). Actin polymerization is an energy-dependent process, which may be reversed by depletion of ATP. This work found that I/R or H/R induced a decrease in junction proteins, an increase in the activity and level of caveolin-1, a disintegrated BM, a disrupted actin and a malregulated energy metabolism, including disfunction of mitochondrial respiratory chain. Pretreatment with QSYQ ameliorated all the insults, demonstrating its multitargeting edge over the currently available measure in containing I/R-caused cardiac microvascular hyperpermeability.

Src family protein tyrosine kinases have been implicated in upstream signaling pathways that lead to endothelial hyperpermeability through both intercellular and transendothelial pathways (Mehta and Malik, 2006). This work showed an increase in activity of Src in HCMECs exposed to H/R, and PP2, the inhibitor of Src, ameliorated the H/R-induced increase in p-caveolin-1, MMP-9, and CTSS and decrease in claudin-5, similar to QSYQ did, suggesting Src/caveolin as one of the signaling that mediates QSYQ effect. As a compound Chinese medicine, QSYQ contains numerous bioactive ingredients, some of which have been reported to exert effect by targeting Src/caveolin-1 signaling. For example, salvianolic acid B was reported to ameliorate lipopolysaccharide-induced albumin leakage from rat mesenteric venules by binding to Src (Pan et al., 2015), Astragaloside IV was shown to enhance taxol chemosensitivity of breast cancer by targeting caveolin-1 (Zheng et al., 2019). Are there any other ingredients in QSYQ that interfere in Src/caveolin-1 signaling remain to be identified? Noticeably, PP2 was less effective than QSYQ in protection of H/R-elicited hyperpermeability of HCMEC monolayer, implying existence of another signaling (s) that participates in mediation of QSYQ effect. Further work is required to identify the target (s) of QSYQ by specially mutating or blocking some potential targets downstream of QSYQ.

This study showed that H/R activated RhoA/ROCK/p-MLC signal in HCMECs which was attenuated by QSYQ, indicating an involvement of RhoA/ROCK/p-MLC in the mechanism for QSYQ to work. This result is in line with the reports regarding the effect of some QSYQ ingredients, such as ginsenoside Rb1 and R1, which were shown able to inhibit RhoA/ROCK meanwhile elevate ATP5D expression and ATP level (He et al., 2014; Cui et al., 2017), protecting I/R-induced energy metabolism disorder. Thus, in this case, among the ingredients of QSYQ, at least ginsenoside Rb1 and R1 contribute to the regulation of the disordered energy metabolism after I/R or H/R by targeting RhoA/ROCK signaling. In addition, the result of this study unraveled that H/R resulted in a decrease in NDUFA12 and YME1L1 alike, the two proteins essential for respiratory chain complex I with the former being one of the subunits of the complex while the later an ATP-dependent metalloprotease that regulates the function of the complex. The reduction in the two proteins partially accounts for the reduced activity of complex I in response to H/R challenge. QSYQ pretreatment restrained the decrease in these two proteins after H/R as well. Dihydroxylphenyl lactic acid, an ingredient of QSYQ, has been reported having potential to restore respiratory chain complex I activity by preserving NDUFA 10 *via* activation of Sirt1 (Yang et al., 2015), which certainly played a role as well in this case in attenuation of complex I dysfunction observed. On the other hand, the ingredient (s) in QSYQ responsible for protection of NDUFA12 and YME1L1 from decrease after H/R is so far unknown and needs to be identified by further research. Nevertheless, this result demonstrated once again the pleiotropic effects of QSYQ on I/R-caused cardiac vascular hyperpermeability.

In conclusion, this study showed that the cardiac microvascular hyperpermeability after I/R took place by a mechanism implicating multiple signaling, and QSYQ presented as an option to deal with this insult with advantage of targeting at least Src/caveolin-1 and RhoA/ROCK/MLC signaling.

## Data Availability Statement

The original contributions presented in the study are included in the article/Supplementary Material, further inquiries can be directed to the corresponding author/s.

## Author Contributions

C-SP performed the *in vitro* experiments, analyzed the data, and wrote the manuscript. Y-YL provided scientific guidance and oversight. LY and S-QL contributed to the animal experiments. KH and Y-CC contributed to confocal imaging. B-HH, XC, and X-RZ participated in the preparation of sample for a transmission electron microscope analysis. J-YF and J-YH revised the article. J-YH supervised the research and provided key research directions. All authors read and agreed with the final article.

## Funding

This work was supported by the National Natural Science Foundation of China [81273637] for J-YH.

## Conflict of Interest

The authors declare that the research was conducted in the absence of any commercial or financial relationships that could be construed as a potential conflict of interest.

## Publisher's Note

All claims expressed in this article are solely those of the authors and do not necessarily represent those of their affiliated organizations, or those of the publisher, the editors and the reviewers. Any product that may be evaluated in this article, or claim that may be made by its manufacturer, is not guaranteed or endorsed by the publisher.

## References

[B1] Alluri H. Grimsley M. Anasooya Shaji C. Varghese K. P. Zhang S. L. Peddaboina C.. (2016). Attenuation of blood-brain barrier breakdown and hyperpermeability by calpain inhibition. J. Biol. Chem. 291, 26958–26969. 10.1074/jbc.M116.735365 27875293 PMC5207131

[B2] Brown R. Nath S. Lora A. Samaha G. Elgamal Z. Kaiser R.. (2020). Cathepsin S: investigating an old player in lung disease pathogenesis, comorbidities, and potential therapeutics. Respir. Res. 21:111. 10.1186/s12931-020-01381-5 32398133 PMC7216426

[B3] Cui Y. C. Pan C. S. Yan L. Li L. Hu B. H. Chang X.. (2017). Ginsenoside Rb1 protects against ischemia/reperfusion-induced myocardial injury via energy metabolism regulation mediated by RhoA signaling pathway. Sci. Rep. 7:44579. 10.1038/srep44579 28327605 PMC5361119

[B4] El-Hattab A. W. Suleiman J. Almannai M. Scaglia F. (2018). Mitochondrial dynamics: Biological roles, molecular machinery, and related diseases. Mol. Genet. Metab. 125, 315–321. 10.1016/j.ymgme.2018.10.003 30361041

[B5] Erlinge D. Gotberg M. Lang I. Holzer M. Noc M. Clemmensen P.. (2014). Rapid endovascular catheter core cooling combined with cold saline as an adjunct to percutaneous coronary intervention for the treatment of acute myocardial infarction. The CHILL-MI trial: a randomized controlled study of the use of central venous catheter core cooling combined with cold saline as an adjunct to percutaneous coronary intervention for the treatment of acute myocardial infarction. J. Am. Coll. Cardiol. 63, 1857–65. 10.1016/j.jacc.2013.12.027 24509284

[B6] He K. Yan L. Pan C. S. Liu Y. Y. Cui Y. C. Hu B. H.. (2014). ROCK-dependent ATP5D modulation contributes to the protection of notoginsenoside NR1 against ischemia-reperfusion-induced myocardial injury. Am. J. Physiol. Heart Circ. Physiol. 307, H1764–76. 10.1152/ajpheart.00259.2014 25305180

[B7] Heusch G. (2004). Postconditioning: old wine in a new bottle? J. Am. Coll. Cardiol. 44, 1111–2. 10.1016/j.jacc.2004.06.013 15337226

[B8] Heusch G. Gersh B. J. (2017). The pathophysiology of acute myocardial infarction and strategies of protection beyond reperfusion: a continual challenge. Eur. Heart J. 38, 774–784. 10.1093/eurheartj/ehw224 27354052

[B9] Heusch G. Libby P. Gersh B. Yellon D. Bohm M. Lopaschuk G.. (2014). Cardiovascular remodelling in coronary artery disease and heart failure. Lancet 383, 1933–1943. 10.1016/S0140-6736(14)60107-0 24831770 PMC4330973

[B10] Katt M. E. Linville R. M. Mayo L. N. Xu Z. S. Searson P. C. (2018). Functional brain-specific microvessels from iPSC-derived human brain microvascular endothelial cells: the role of matrix composition on monolayer formation. Fluids Barriers CNS 15:7. 10.1186/s12987-018-0092-7 29463314 PMC5819713

[B11] Korayem A. H. Mujica P. E. Aramoto H. Durán R. G. Nepali P. R. Kim D. D.. (2017). Endothelial cAMP deactivates ischemia-reperfusion-induced microvascular hyperpermeability via Rap1-mediated mechanisms. Am. J. Physiol. Heart Circ. Physiol. 313, H179–H189. 10.1152/ajpheart.00002.2017 28476918 PMC5538859

[B12] Kottke M. A. Walters T. J. (2016). Where's the leak in vascular barriers? A review. Shock 46, 20–36. 10.1097/SHK.0000000000000666 27405062

[B13] Li L. Pan C. S. Yan L. Cui Y. C. Liu Y. Y. Mu H. N.. (2018). Ginsenoside Rg1 ameliorates rat myocardial ischemia-reperfusion injury by modulating energy metabolism pathways. Front. Physiol. 9:78. 10.3389/fphys.2018.00078 29467677 PMC5808323

[B14] Lin S. Q. Wei X. H. Huang P. Liu Y. Y. Zhao N. Li Q.. (2013). QiShenYiQi Pills(R). prevent cardiac ischemia-reperfusion injury via energy modulation. Int. J. Cardiol. 168, 967–974. 10.1016/j.ijcard.2012.10.042 23168012

[B15] Mehta D. Malik A. B. (2006). Signaling mechanisms regulating endothelial permeability. Physiol. Rev. 86, 279–367. 10.1152/physrev.00012.2005 16371600

[B16] Montalescot G. van 't Hof A. W. Lapostolle F. Silvain J. Lassen J. F. Bolognese L.. (2014). Prehospital ticagrelor in ST-segment elevation myocardial infarction. N. Engl. J. Med. 371, 1016–1027. 10.1056/NEJMoa1407024 25175921

[B17] Ostergaard E. Rodenburg R. J. van den Brand M. Thomsen L. L. Duno M. Batbayli M.. (2011). Respiratory chain complex I deficiency due to NDUFA12 mutations as a new cause of Leigh syndrome. J. Med. Genet. 48, 737–740. 10.1136/jmg.2011.088856 21617257

[B18] Pan C. S. Liu Y. H. Liu Y. Y. Zhang Y. He K. Yang X. Y.. (2015). Salvianolic acid B ameliorates lipopolysaccharide-induced albumin leakage from rat mesenteric venules through Src-regulated transcelluar pathway and paracellular pathway. PLoS ONE 10:e0126640. 10.1371/journal.pone.0126640 25992563 PMC4438061

[B19] Pries A. R. Kuebler W. M. (2006). Normal endothelium. Handb. Experi. Pharmacol. 176, 1–40. 10.1007/3-540-32967-6_1 16999215

[B20] Rak M. Rustin P. (2014). Supernumerary subunits NDUFA3, NDUFA5 and NDUFA12 are required for the formation of the extramembrane arm of human mitochondrial complex I. FEBS Lett. 588, 1832–8. 10.1016/j.febslet.2014.03.046 24717771

[B21] Selker H. P. Beshansky J. R. Sheehan P. R. Massaro J. M. Griffith J. L. D'Agostino R. B.. (2012). Out-of-hospital administration of intravenous glucose-insulin-potassium in patients with suspected acute coronary syndromes: the IMMEDIATE randomized controlled trial. JAMA 307, 1925–33. 10.1001/jama.2012.426 22452807 PMC4167391

[B22] Shen W. C. Chou Y. H. Huang H. P. Sheen J. F. Hung S. C. Chen H. F. (2018). Induced pluripotent stem cell-derived endothelial progenitor cells attenuate ischemic acute kidney injury and cardiac dysfunction. Stem Cell Res. Ther. 9:344. 10.1186/s13287-018-1092-x 30526689 PMC6288873

[B23] Stiburek L. Cesnekova J. Kostkova O. Fornuskova D. Vinsova K. Wenchich L.. (2012). YME1L controls the accumulation of respiratory chain subunits and is required for apoptotic resistance, cristae morphogenesis, and cell proliferation. Mol. Biol. Cell 23, 1010–1023. 10.1091/mbc.e11-08-0674 22262461 PMC3302729

[B24] Tu L. Pan C. S. Wei X. H. Yan L. Liu Y. Y. Fan J. Y.. (2013). Astragaloside IV protects heart from ischemia and reperfusion injury via energy regulation mechanisms. Microcirculation 20, 736–747. 10.1111/micc.12074 23809007

[B25] Wallez Y. Huber P. (2008). Endothelial adherens and tight junctions in vascular homeostasis, inflammation and angiogenesis. Biochim. Biophys. Acta 1778, 794–809. 10.1016/j.bbamem.2007.09.003 17961505

[B26] Weis S. M. (2008). Vascular permeability in cardiovascular disease and cancer. Curr. Opin. Hematol. 15, 243–249. 10.1097/MOH.0b013e3282f97d86 18391792

[B27] Yan L. Pan C. S. Liu Y. Y. Cui Y. C. Hu B. H. Chang X.. (2021). The composite of 3, 4-dihydroxyl-phenyl lactic acid and notoginsenoside R1 attenuates myocardial ischemia and reperfusion injury through regulating mitochondrial respiratory chain. Front. Physiol. 12:538962. 10.3389/fphys.2021.538962 34322032 PMC8311465

[B28] Yan L. L. Zhang W. Y. Wei X. H. Yan L. Pan C. S. Yu Y.. (2018). gualou xiebai decoction, a traditional chinese medicine, prevents cardiac reperfusion injury of hyperlipidemia rat via energy modulation. Front. Physiol. 9:296. 10.3389/fphys.2018.00296 29674972 PMC5895855

[B29] Yang Q. He G. W. Underwood M. J. Yu C. M. (2016). Cellular and molecular mechanisms of endothelial ischemia/reperfusion injury: perspectives and implications for postischemic myocardial protection. Am. J. Transl. Res. 8, 765–777. 27158368 PMC4846925

[B30] Yang X. Y. He K. Pan C. S. Li Q. Liu Y. Y. Yan L.. (2015). 3, 4-dihydroxyl-phenyl lactic acid restores NADH dehydrogenase 1 α subunit 10 to ameliorate cardiac reperfusion injury. Sci. Rep. 5:10739. 10.1038/srep10739 26030156 PMC5377067

[B31] Yellon D. M. Hausenloy D. J. (2007). Myocardial reperfusion injury. N. Engl. J. Med. 357, 1121–1135. 10.1056/NEJMra071667 17855673

[B32] Zhang L. Wang Y. Yu L. Liu L. Qu H. Gao X.. (2010). QI-SHEN-YI-QI accelerates angiogenesis after myocardial infarction in rats. Int. J. Cardiol. 143, 105–109. 10.1016/j.ijcard.2008.11.210 19203810

[B33] Zhang Y. Shi P. Yao H. Shao Q. Fan X. (2012). Metabolite profiling and pharmacokinetics of herbal compounds following oral administration of a cardiovascular multi-herb medicine (Qishen yiqi pills). in rats. Curr. Drug Metab. 13, 510–523. 10.2174/1389200211209050510 22292791

[B34] Zhao J. L. Yang Y. J. You S. J. Jing Z. C. Wu Y. J. Cheng J. L.. (2006). Pretreatment with fosinopril or valsartan reduces myocardial no-reflow after acute myocardial infarction and reperfusion. Coron. Artery Dis. 17, 463–469 10.1097/00019501-200608000-00010 16845255

[B35] Zheng Y. Dai Y. Liu W. Wang N. Cai Y. Wang S.. (2019). Astragaloside IV enhances taxol chemosensitivity of breast cancer via caveolin-1-targeting oxidant damage. J. Cell. Physiol. 234, 4277–4290. 10.1002/jcp.27196 30146689

