# Supplementary material for: QiShenYiQi Pills Attenuates Ischemia/Reperfusion-Induced Cardiac Microvascular Hyperpermeability Implicating Src/Caveolin-1 and RhoA/ROCK/MLC Signaling
Source: Front Physiol. 2021 Dec 17;12:753761. doi: 10.3389/fphys.2021.753761 (PMC8718710; doi:10.3389/fphys.2021.753761)
Supplement: Supplementary file 1 [file Presentation_1.PPTX]

## Slide 1
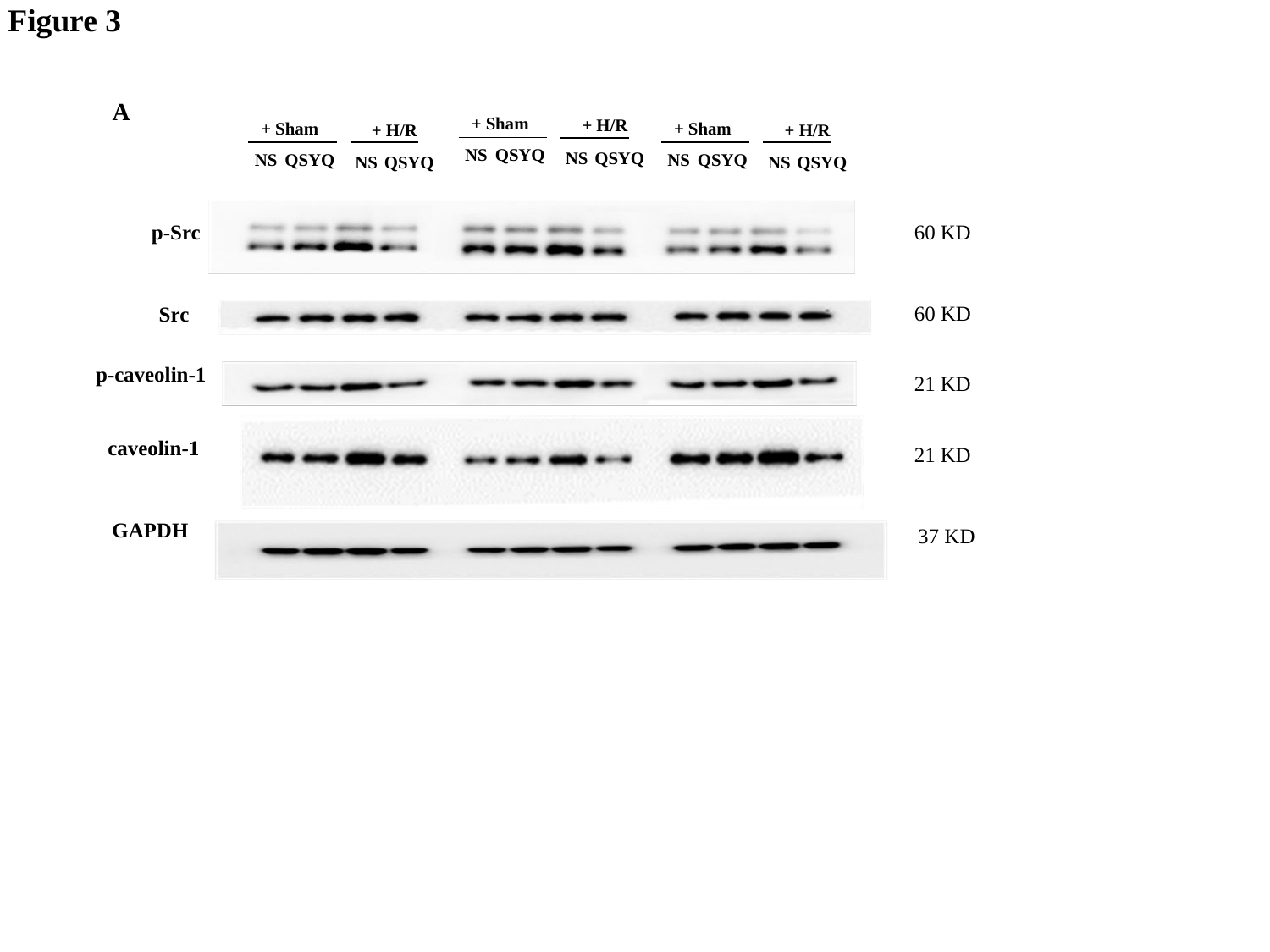

Figure 3
A
+ Sham
+ H/R
NS
QSYQ
QSYQ
NS
+ Sham
+ H/R
NS
QSYQ
QSYQ
NS
+ Sham
+ H/R
NS
QSYQ
QSYQ
NS
p-Src
60 KD
60 KD
Src
p-caveolin-1
21 KD
caveolin-1
21 KD
GAPDH
37 KD

## Slide 2
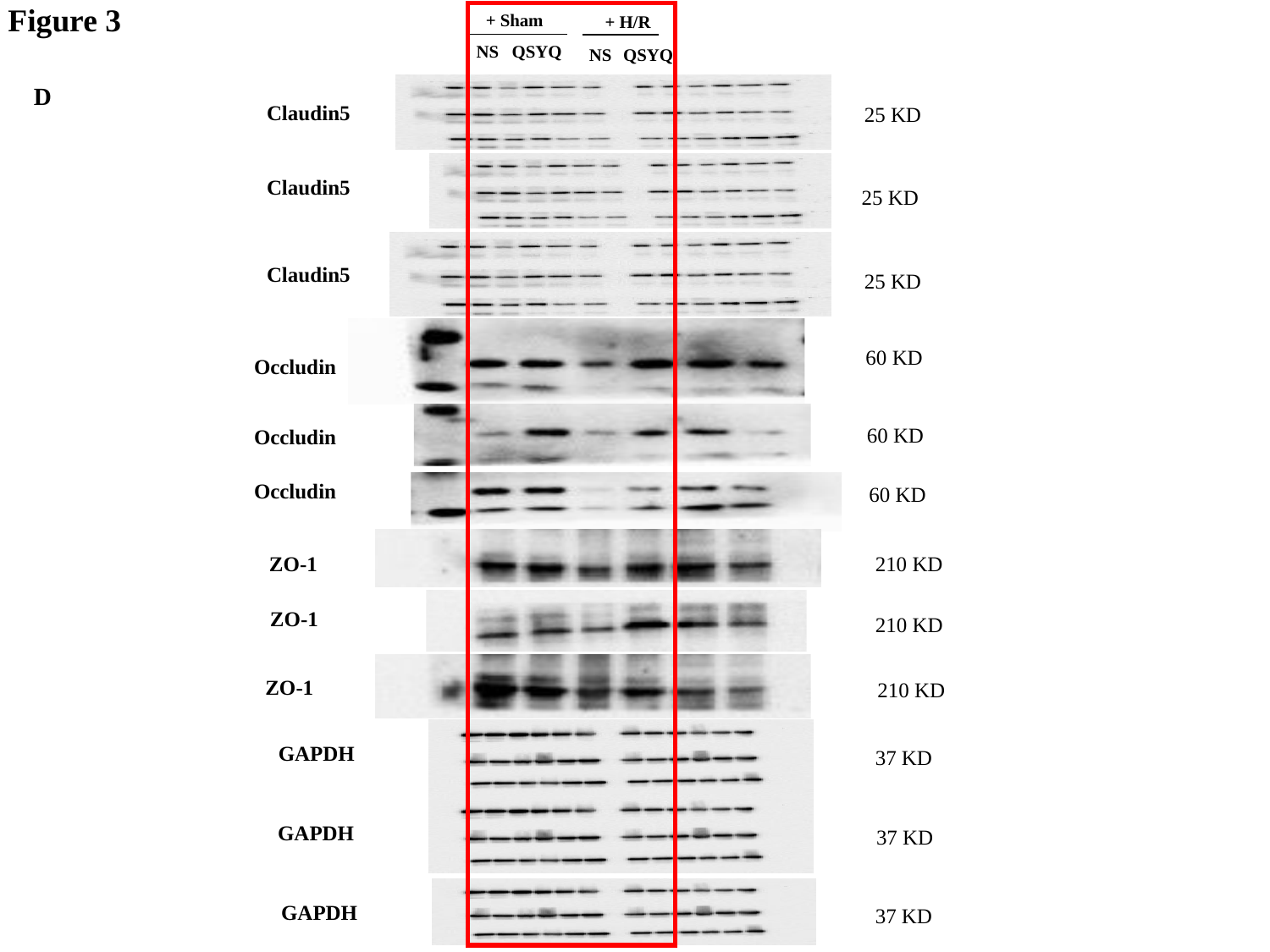

Figure 3
+ Sham
+ H/R
NS
QSYQ
QSYQ
NS
Claudin5
25 KD
Claudin5
25 KD
Claudin5
25 KD
60 KD
Occludin
60 KD
Occludin
Occludin
60 KD
ZO-1
210 KD
ZO-1
210 KD
ZO-1
210 KD
GAPDH
37 KD
GAPDH
37 KD
GAPDH
37 KD
D

## Slide 3
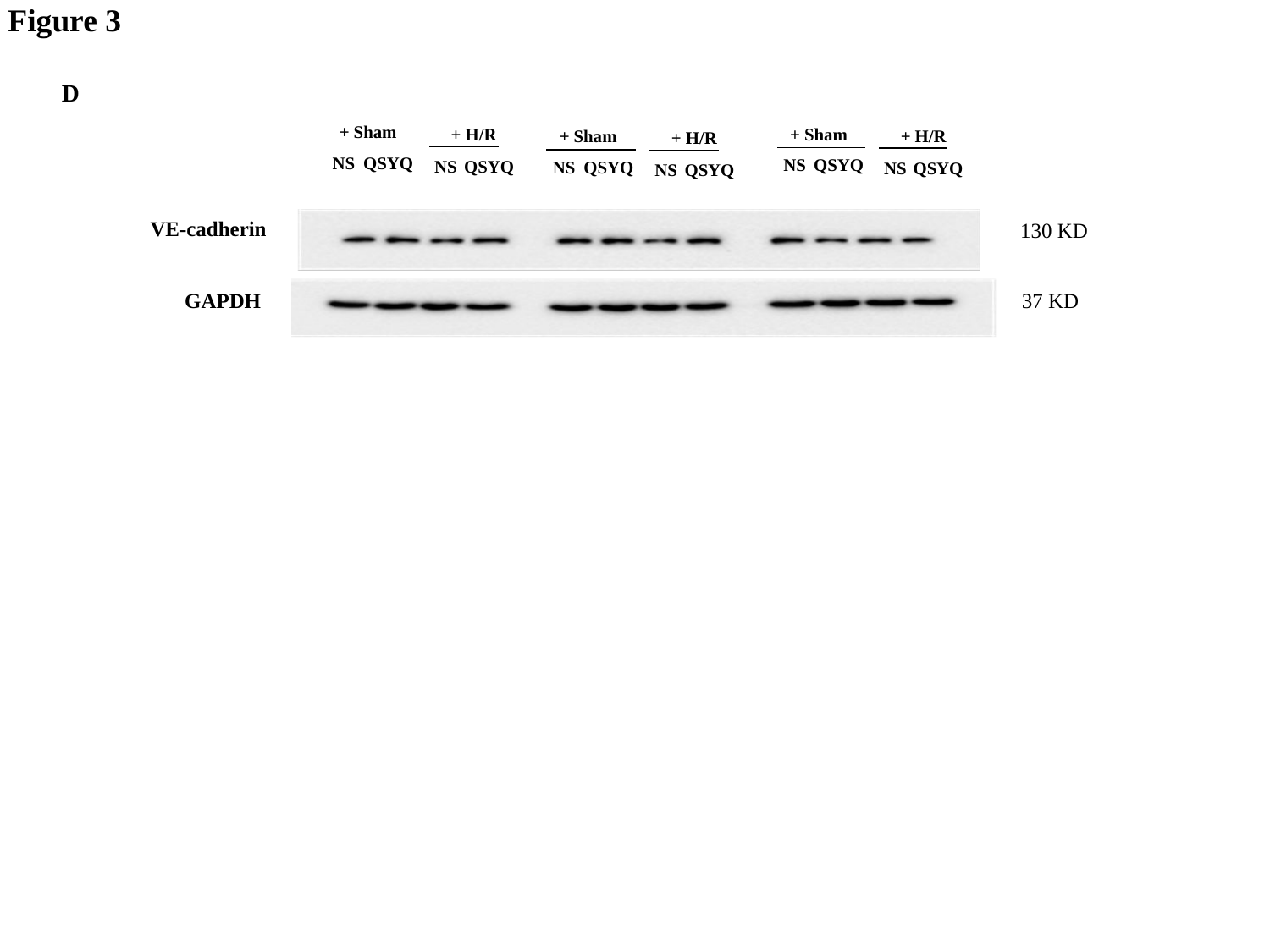

Figure 3
D
+ Sham
+ H/R
NS
QSYQ
QSYQ
NS
+ Sham
+ H/R
NS
QSYQ
QSYQ
NS
+ Sham
+ H/R
NS
QSYQ
QSYQ
NS
VE-cadherin
130 KD
GAPDH
37 KD

## Slide 4
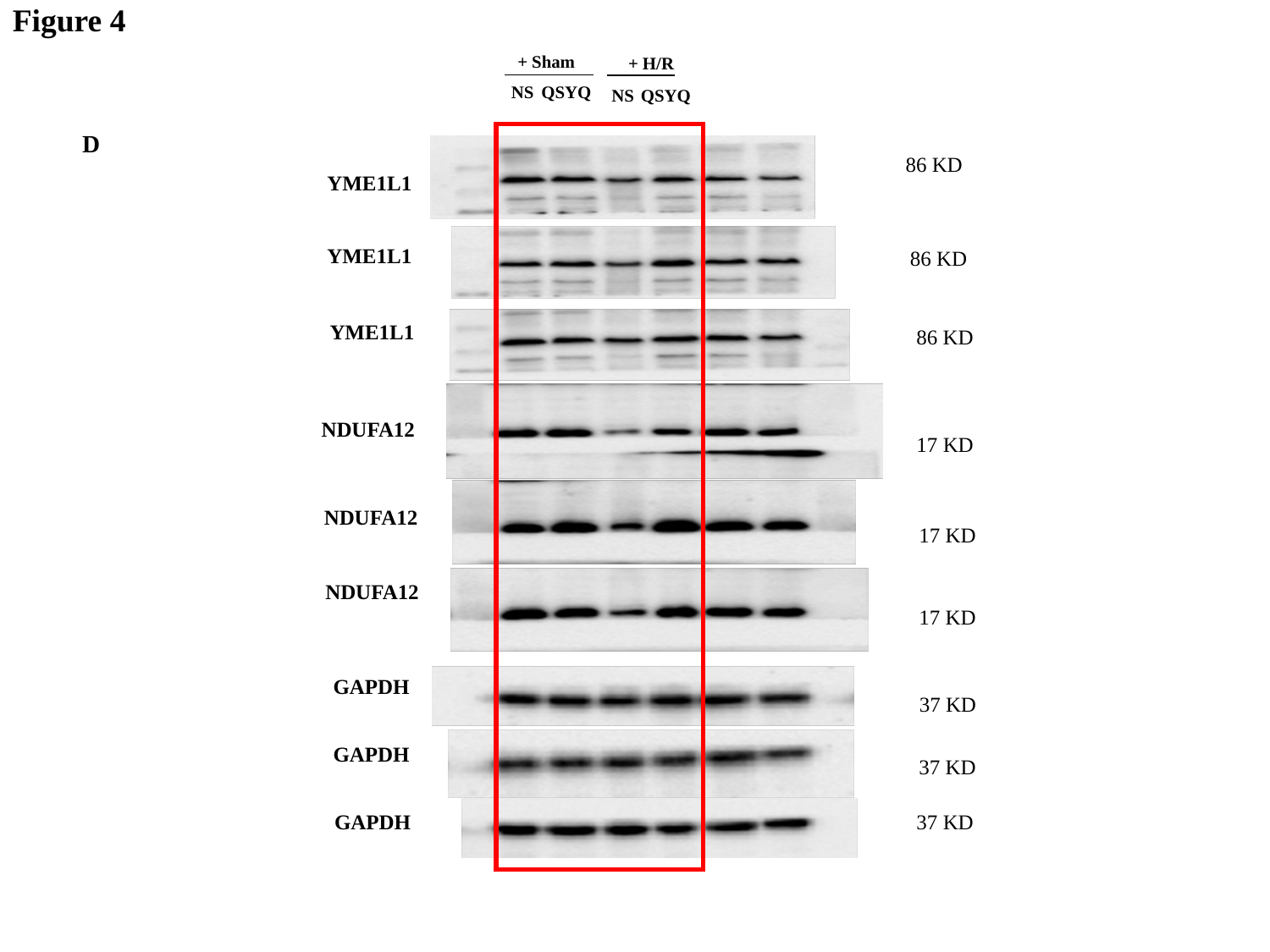

Figure 4
+ Sham
+ H/R
NS
QSYQ
QSYQ
NS
D
86 KD
YME1L1
YME1L1
86 KD
YME1L1
86 KD
NDUFA12
17 KD
NDUFA12
17 KD
NDUFA12
17 KD
GAPDH
37 KD
GAPDH
37 KD
GAPDH
37 KD

## Slide 5
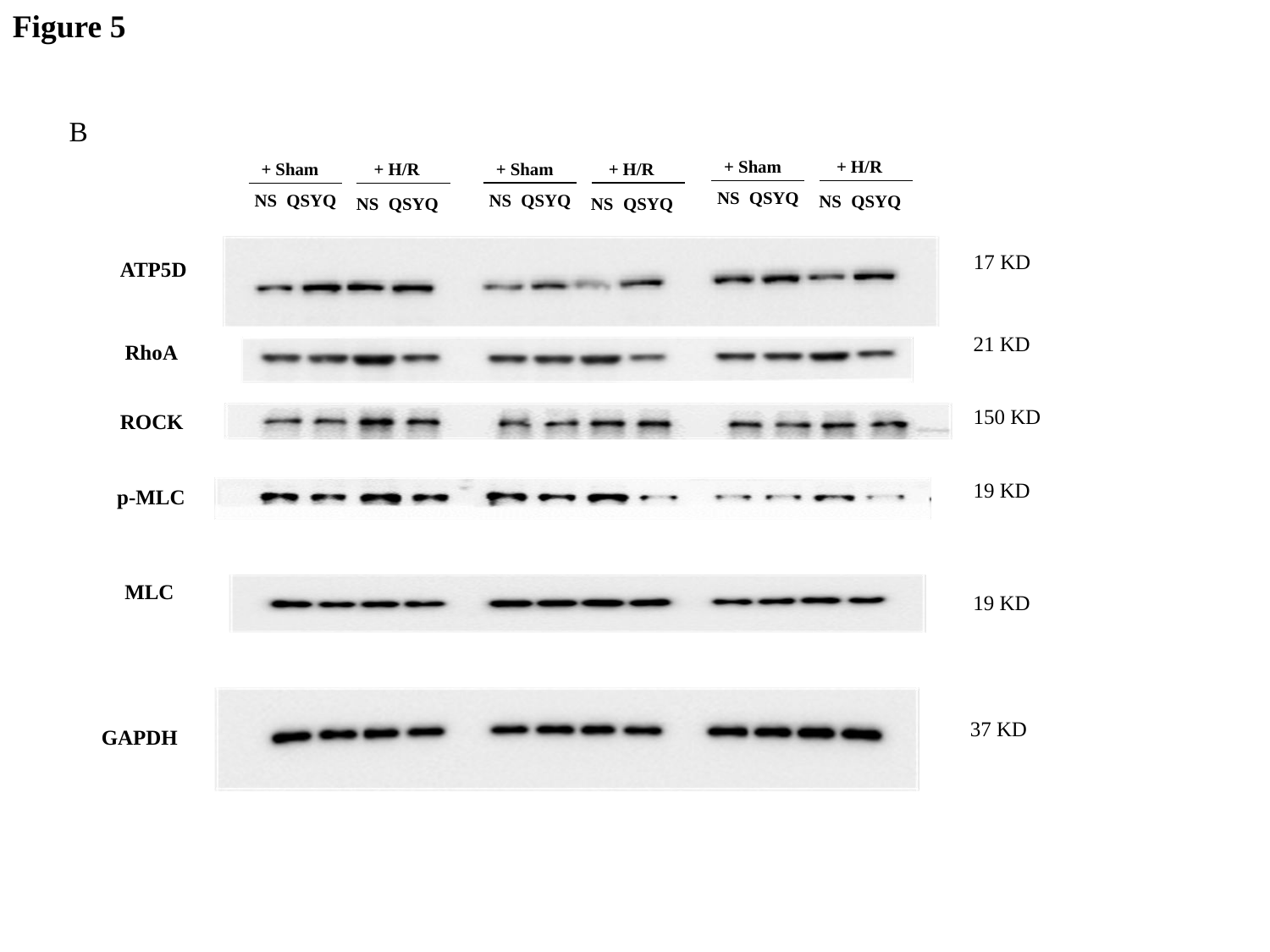

Figure 5
B
+ Sham
+ H/R
NS
QSYQ
QSYQ
NS
+ Sham
+ H/R
NS
QSYQ
QSYQ
NS
+ Sham
+ H/R
NS
QSYQ
QSYQ
NS
17 KD
ATP5D
21 KD
RhoA
150 KD
ROCK
19 KD
p-MLC
MLC
19 KD
37 KD
GAPDH

## Slide 6
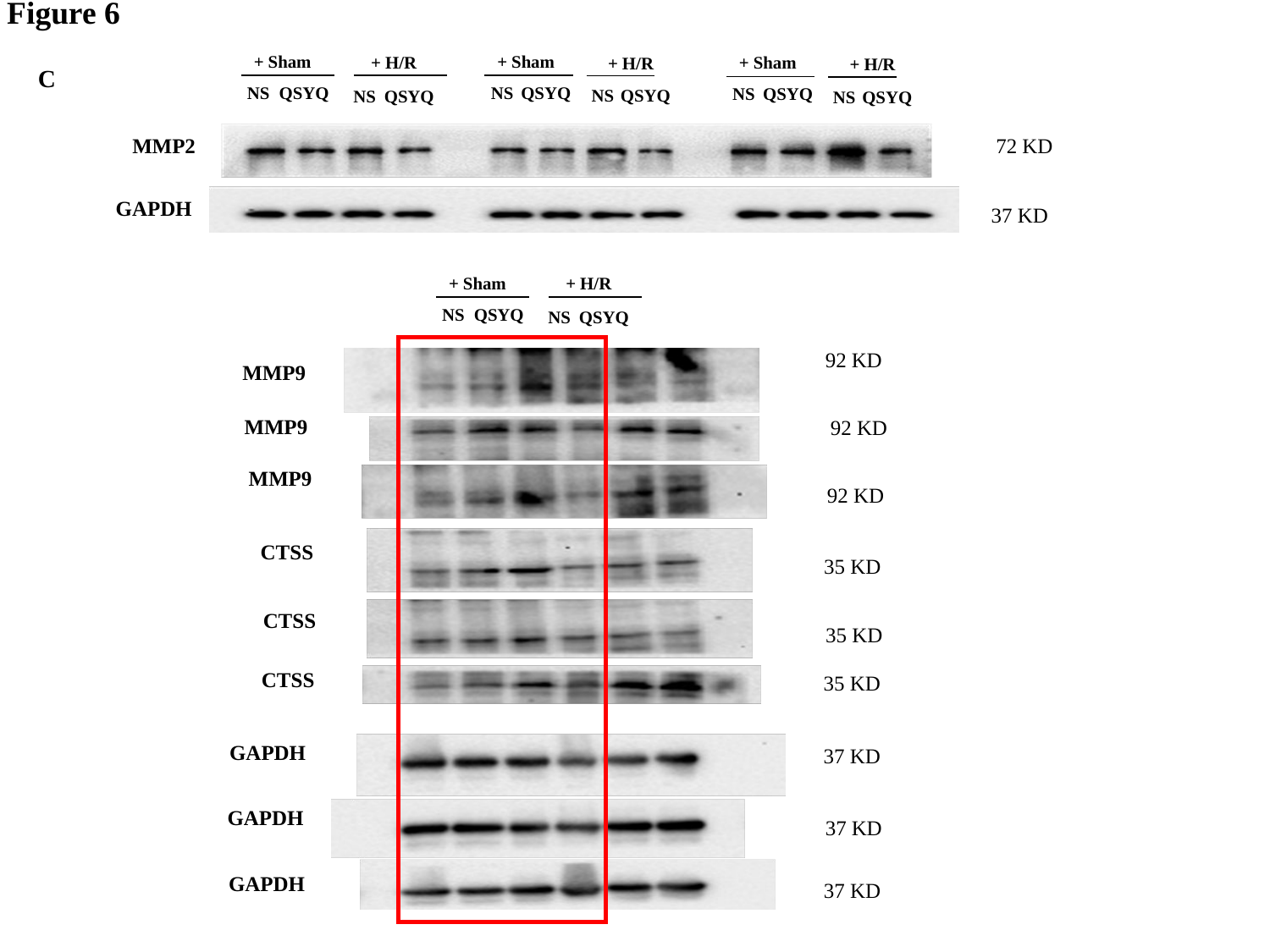

Figure 6
+ Sham
+ H/R
NS
QSYQ
QSYQ
NS
+ Sham
+ H/R
NS
QSYQ
QSYQ
NS
+ Sham
+ H/R
NS
QSYQ
QSYQ
NS
C
MMP2
72 KD
GAPDH
37 KD
+ Sham
+ H/R
NS
QSYQ
QSYQ
NS
92 KD
MMP9
MMP9
92 KD
MMP9
92 KD
CTSS
35 KD
CTSS
35 KD
CTSS
35 KD
GAPDH
37 KD
GAPDH
37 KD
GAPDH
37 KD

## Slide 7
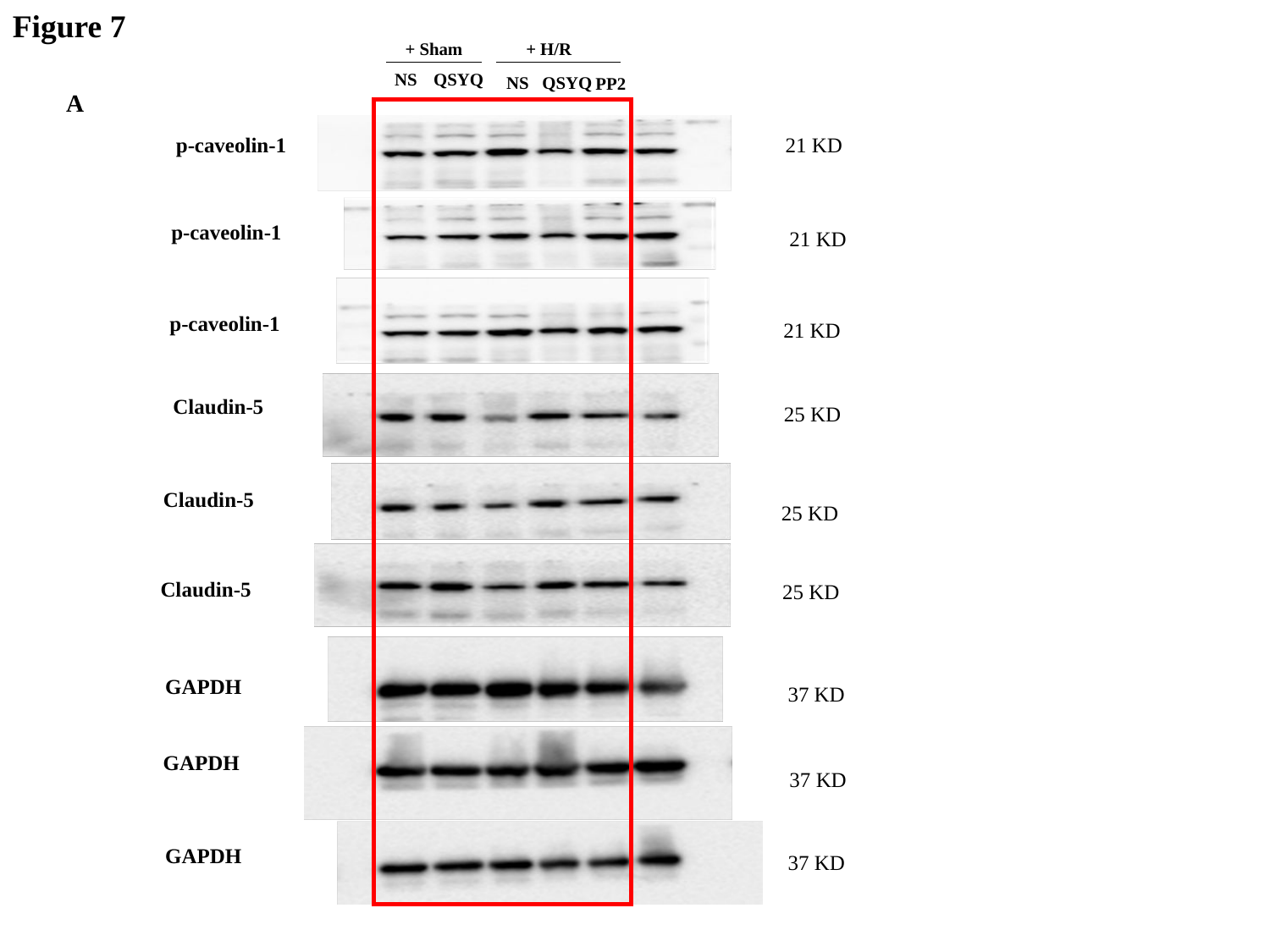

Figure 7
+ Sham
+ H/R
NS
QSYQ
QSYQ
NS
PP2
A
p-caveolin-1
21 KD
p-caveolin-1
21 KD
p-caveolin-1
21 KD
Claudin-5
25 KD
Claudin-5
25 KD
Claudin-5
25 KD
GAPDH
37 KD
GAPDH
37 KD
GAPDH
37 KD
