# Supplementary material for: QiShenYiQi Pills Attenuates Ischemia/Reperfusion-Induced Cardiac Microvascular Hyperpermeability Implicating Src/Caveolin-1 and RhoA/ROCK/MLC Signaling
Source: Front Physiol. 2021 Dec 17;12:753761. doi: 10.3389/fphys.2021.753761 (PMC8718710; doi:10.3389/fphys.2021.753761)
Supplement: Supplementary file 2 [file Presentation_2.PPTX]

## Slide 1
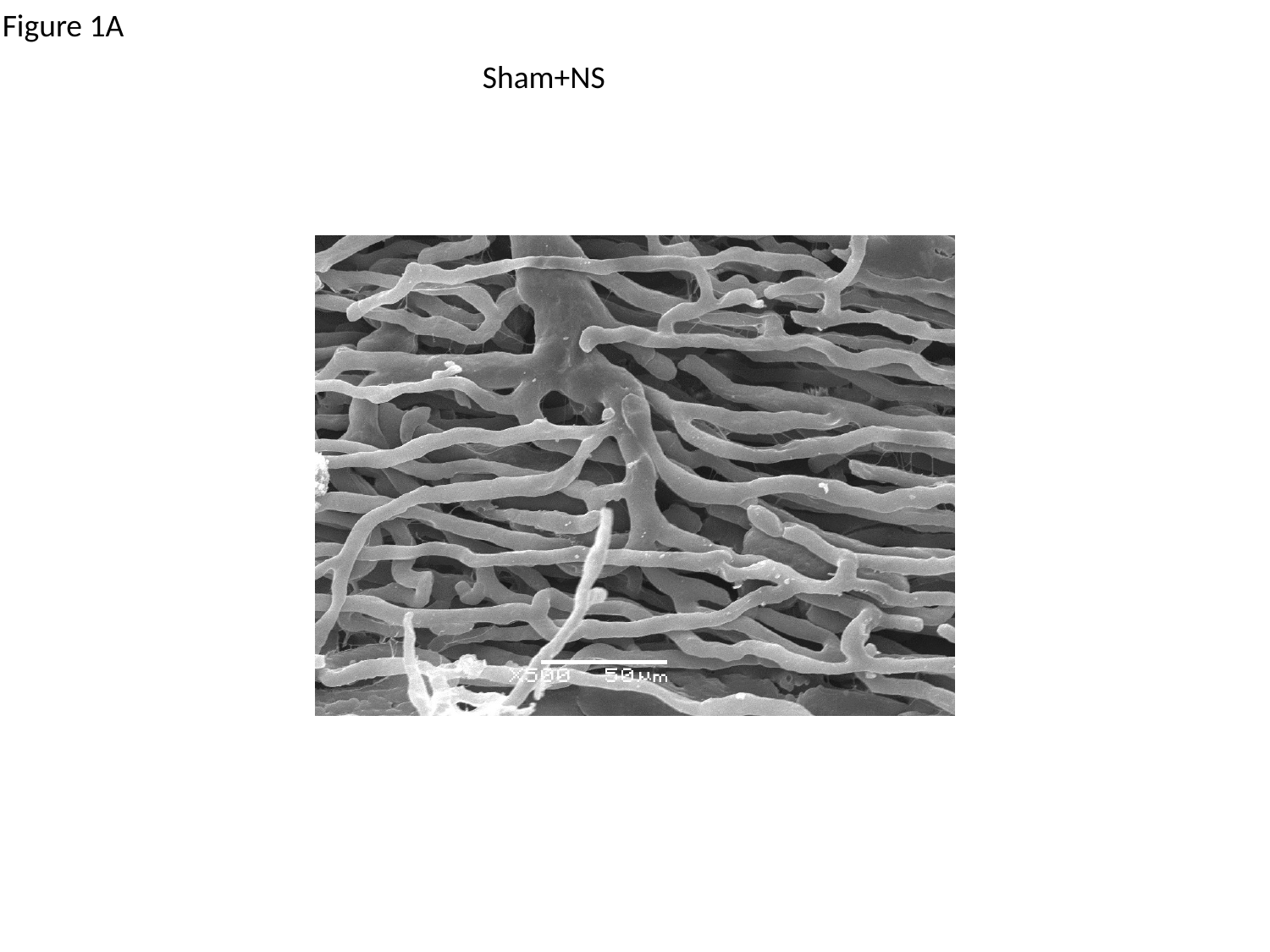

Figure 1A
Sham+NS

## Slide 2
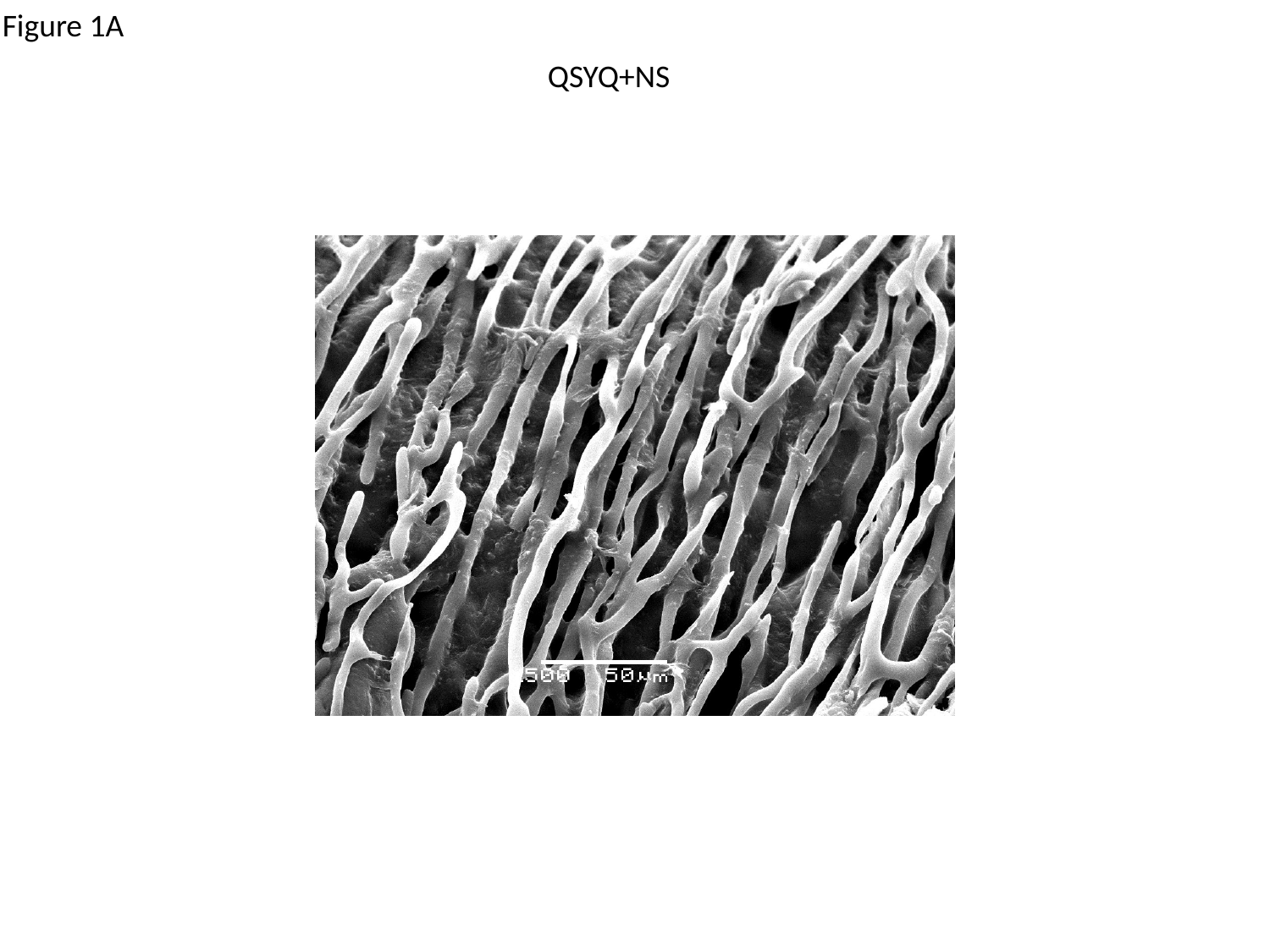

Figure 1A
QSYQ+NS

## Slide 3
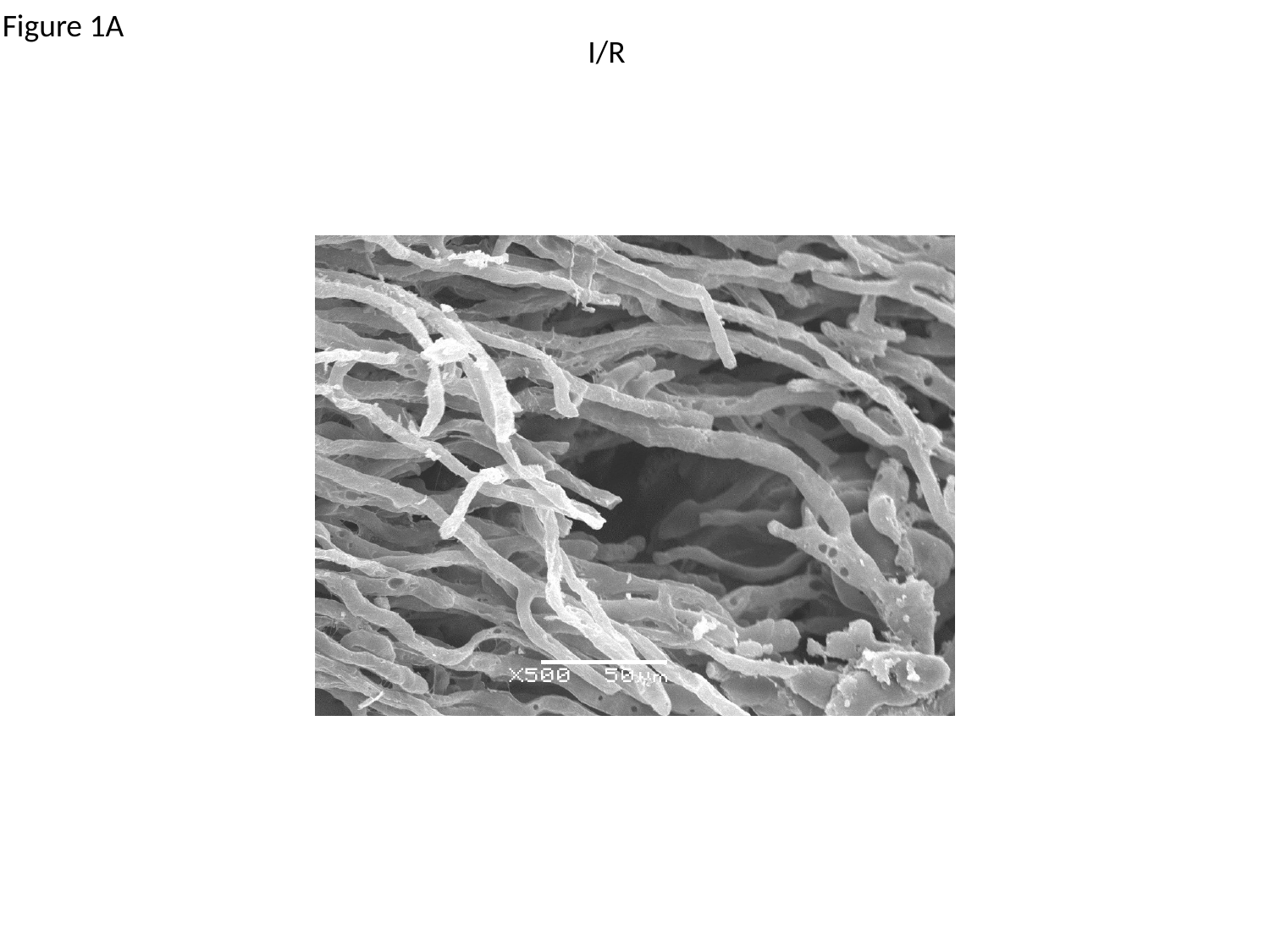

Figure 1A
I/R

## Slide 4
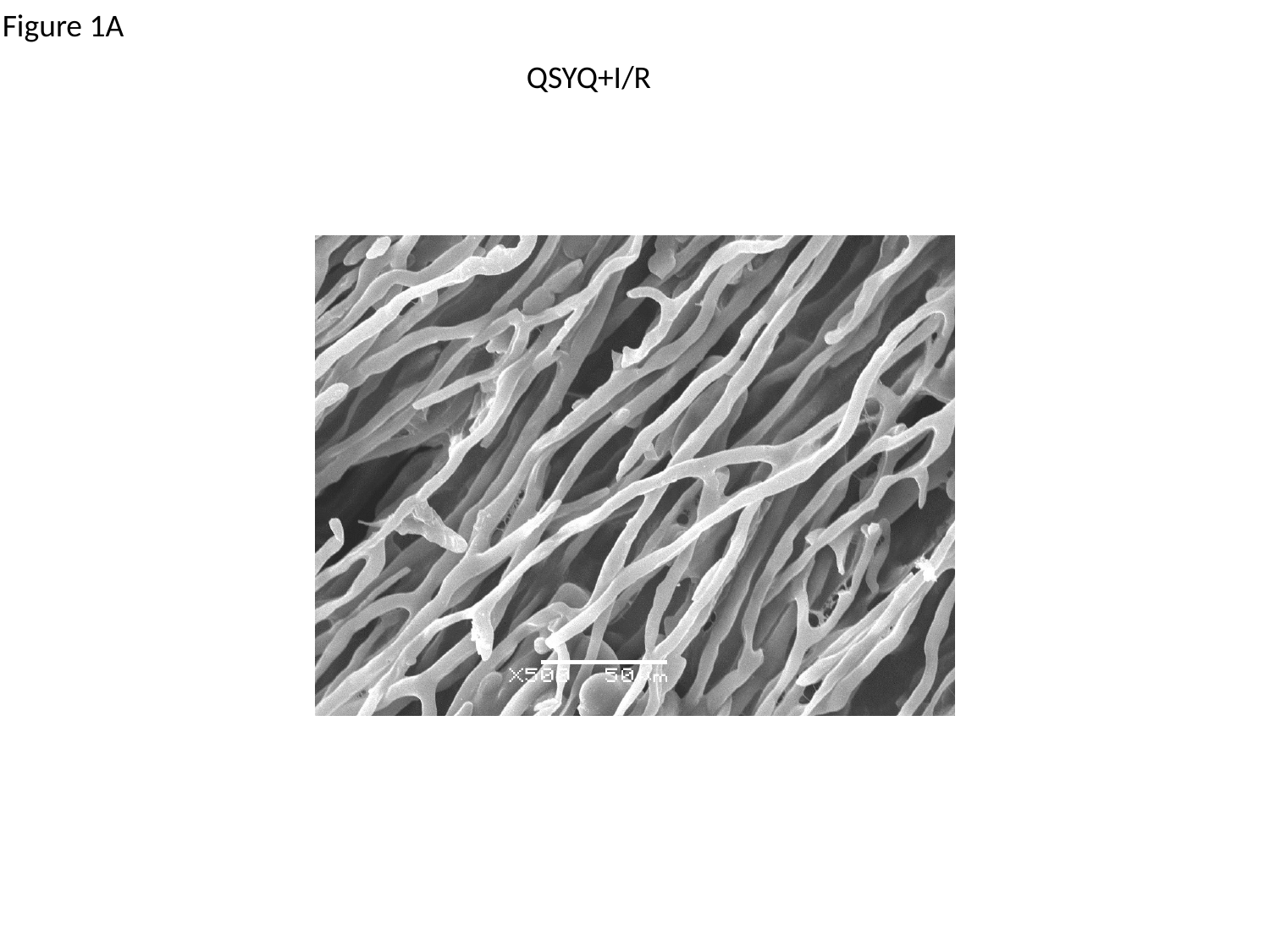

Figure 1A
QSYQ+I/R

## Slide 5
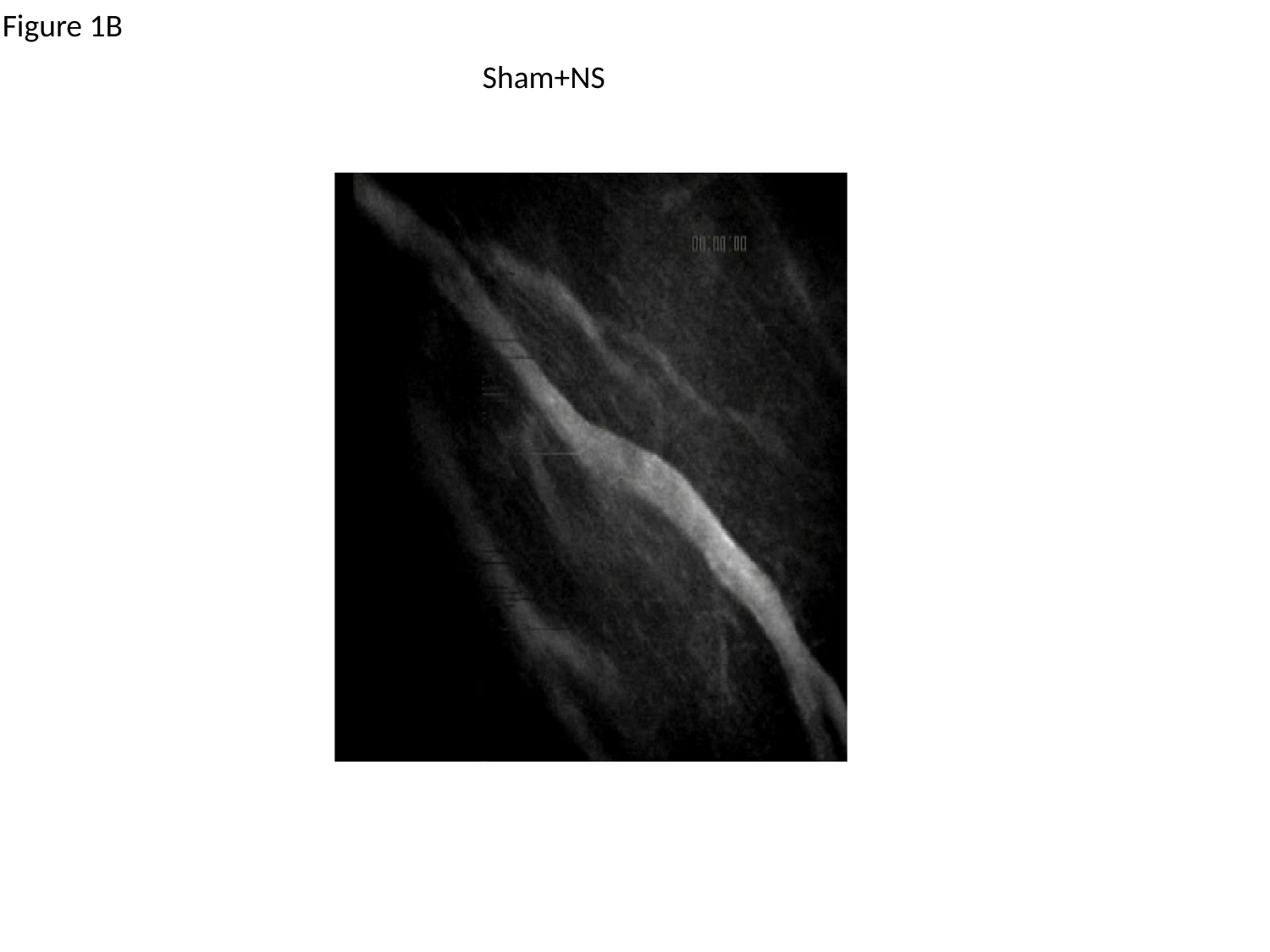

Figure 1B
Sham+NS

## Slide 6
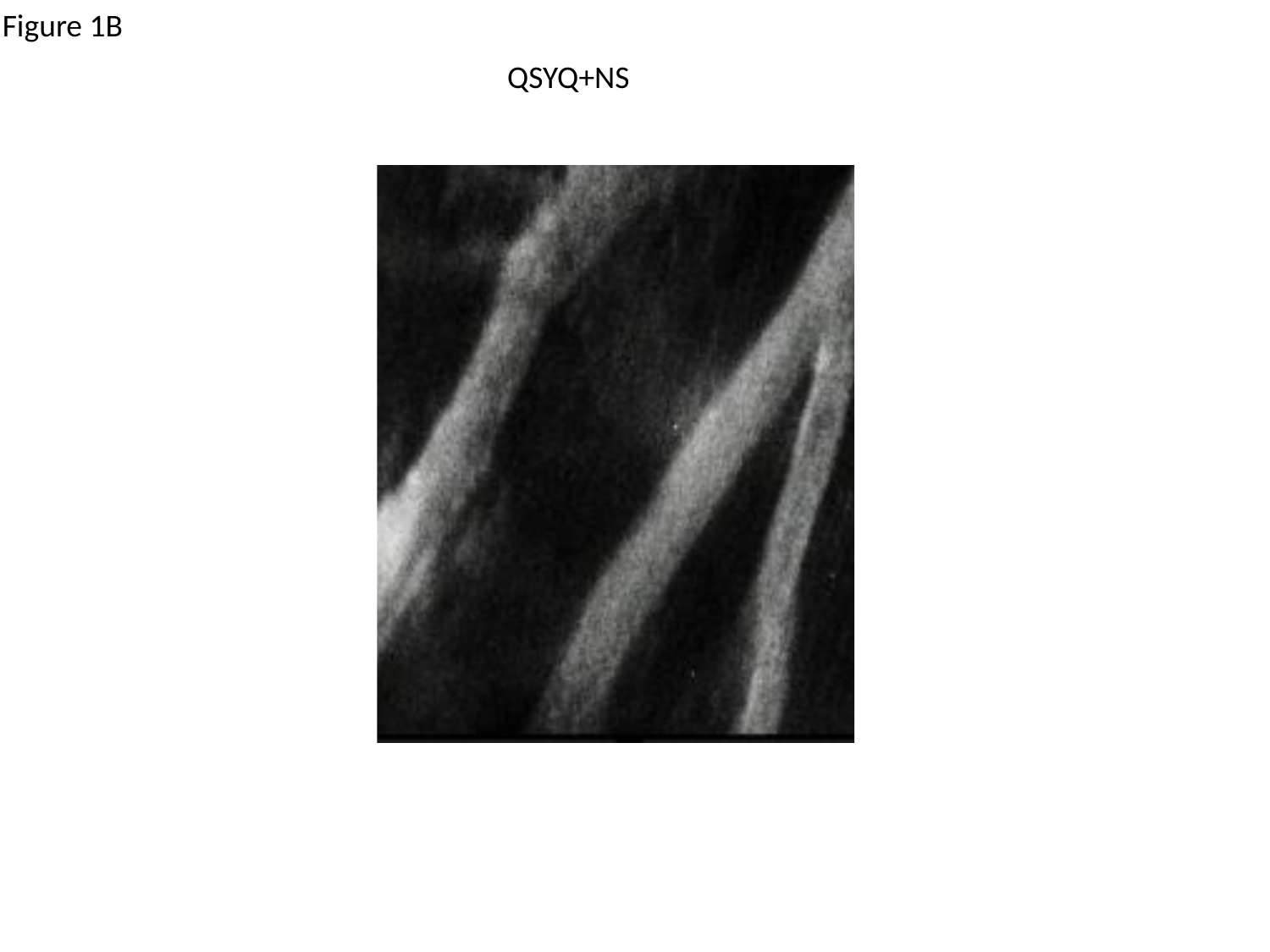

Figure 1B
QSYQ+NS

## Slide 7
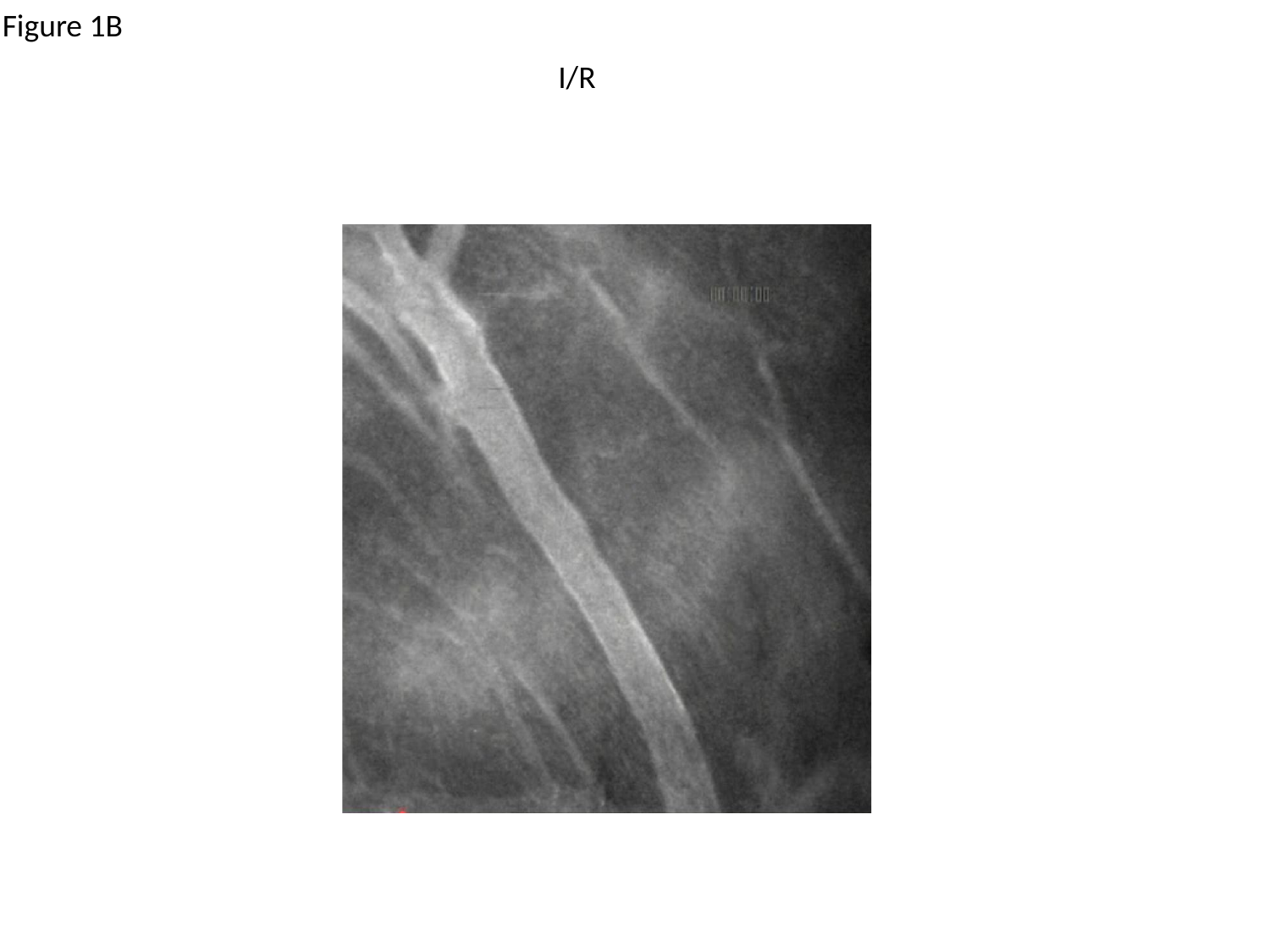

Figure 1B
I/R

## Slide 8
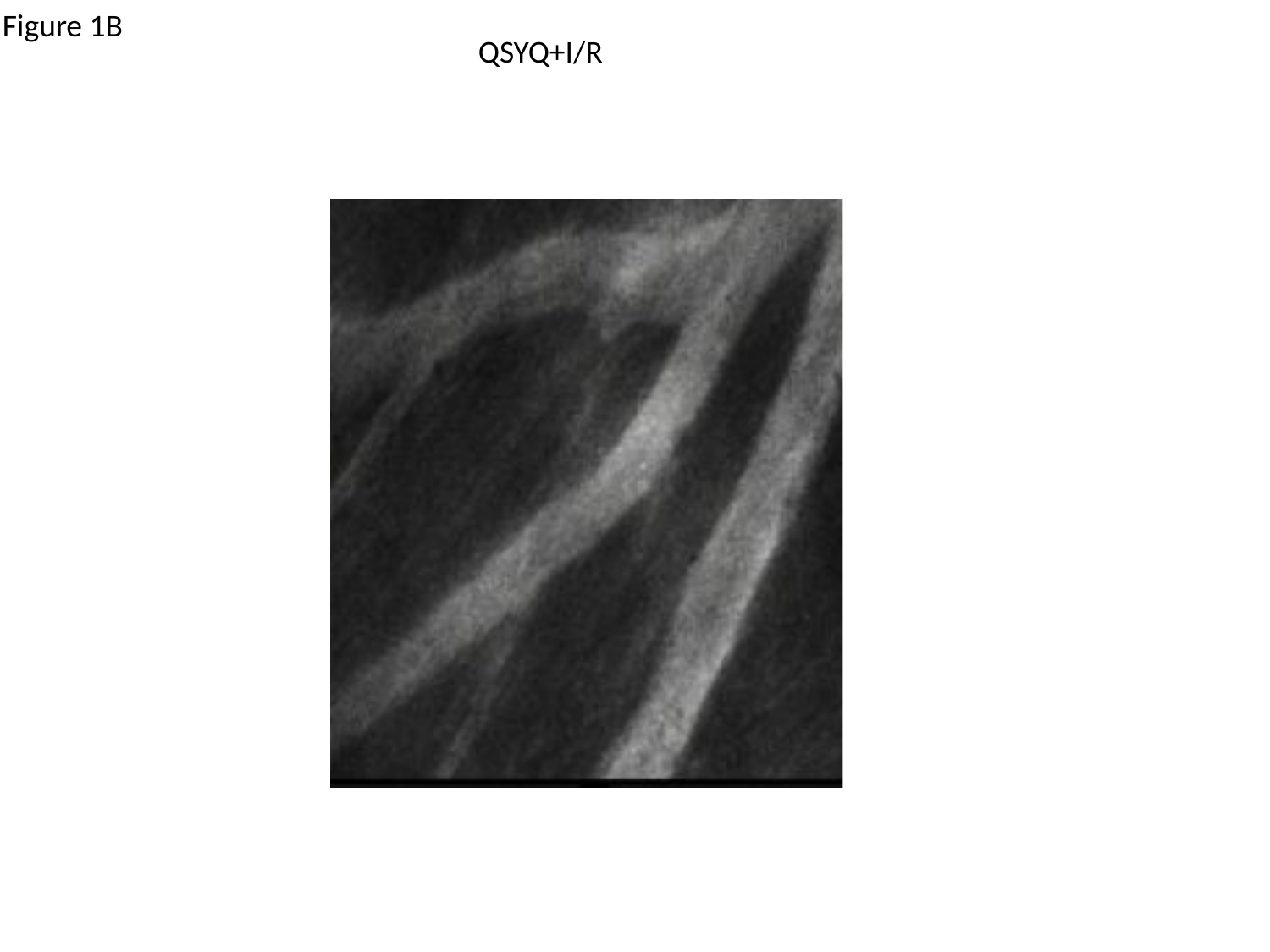

Figure 1B
QSYQ+I/R

## Slide 9
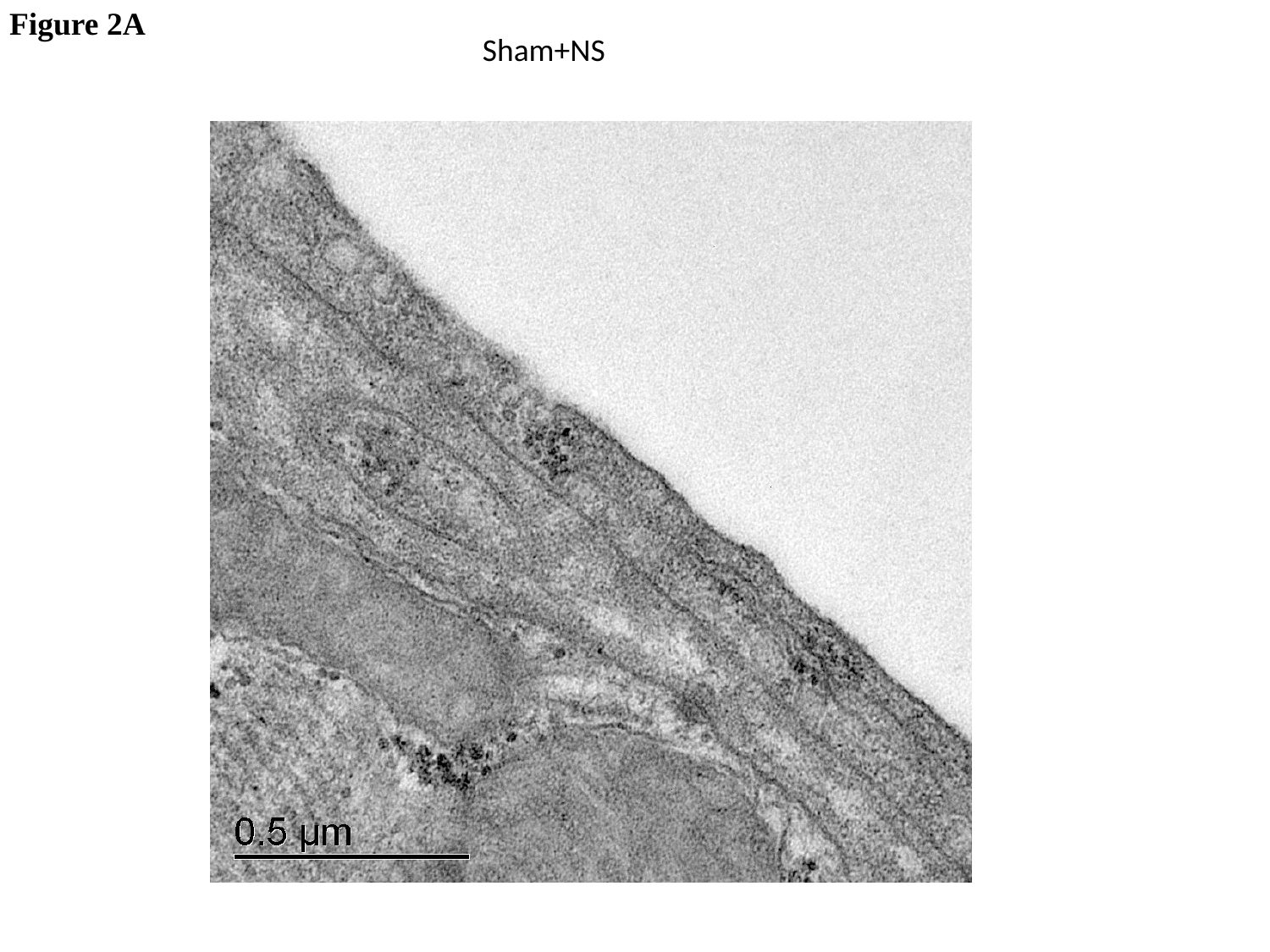

Figure 2A
Sham+NS

## Slide 10
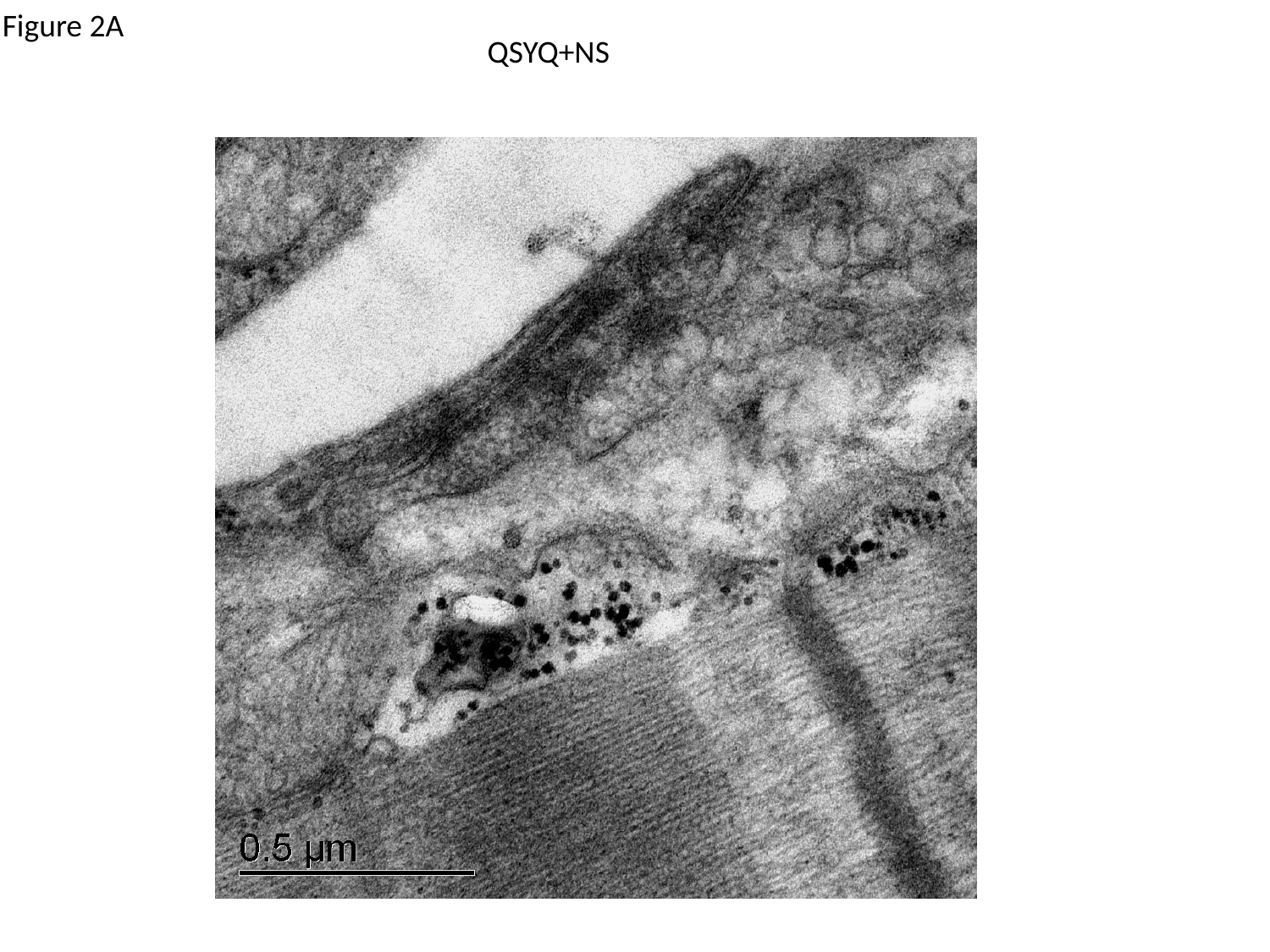

Figure 2A
QSYQ+NS

## Slide 11
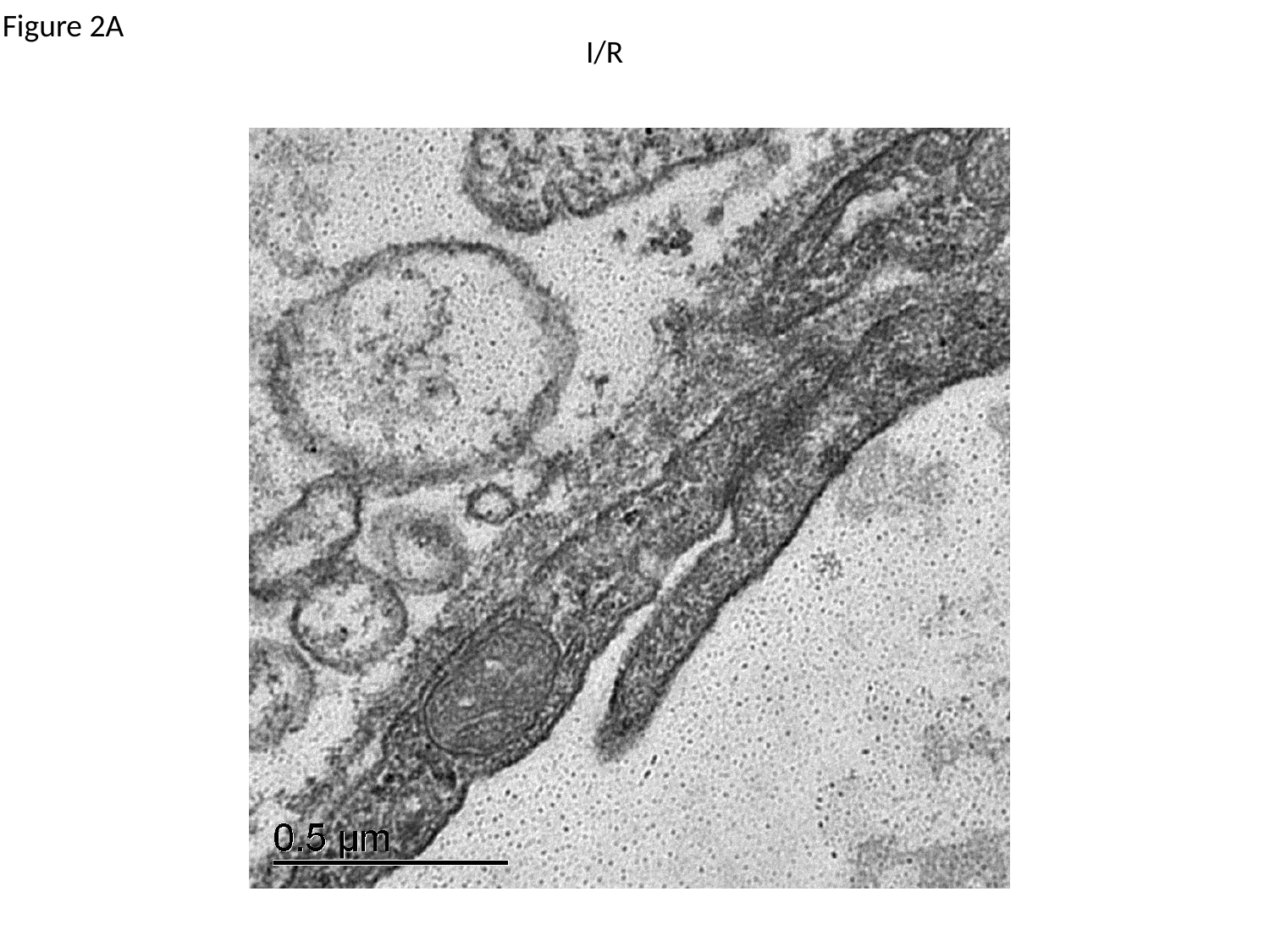

Figure 2A
I/R

## Slide 12
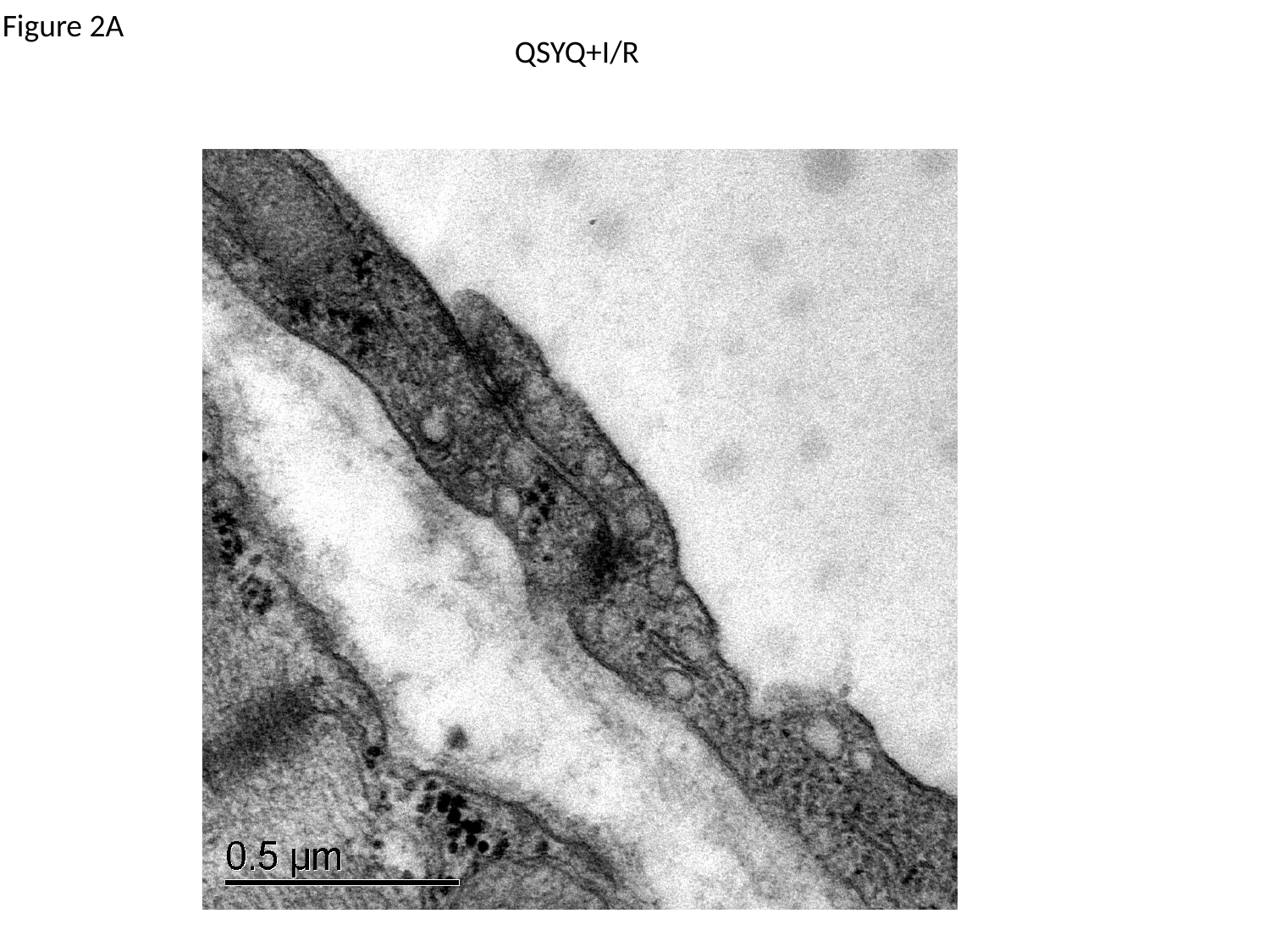

Figure 2A
QSYQ+I/R

## Slide 13
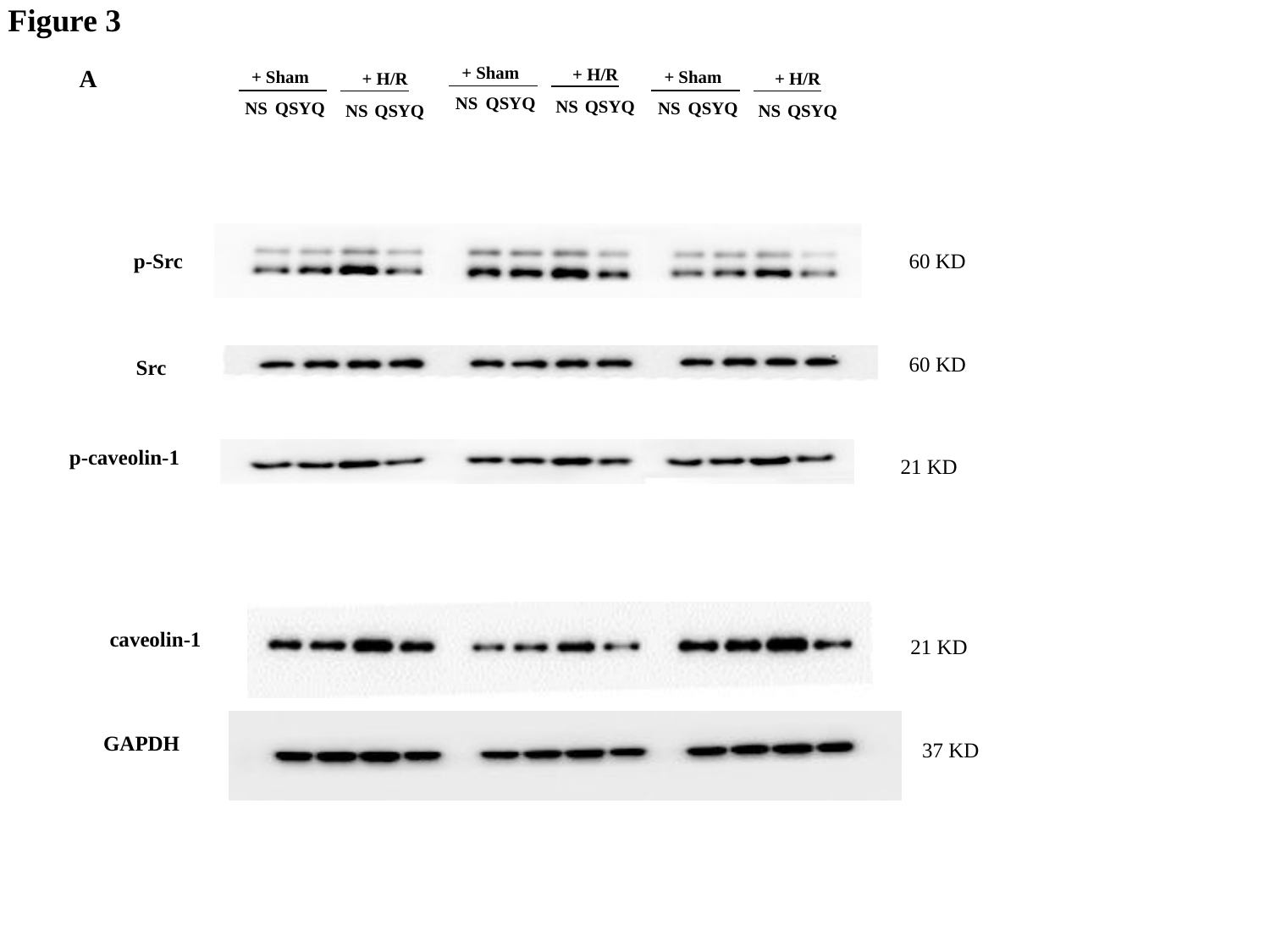

Figure 3
+ Sham
+ H/R
NS
QSYQ
QSYQ
NS
A
+ Sham
+ H/R
NS
QSYQ
QSYQ
NS
+ Sham
+ H/R
NS
QSYQ
QSYQ
NS
60 KD
p-Src
60 KD
Src
p-caveolin-1
21 KD
caveolin-1
21 KD
GAPDH
37 KD

## Slide 14
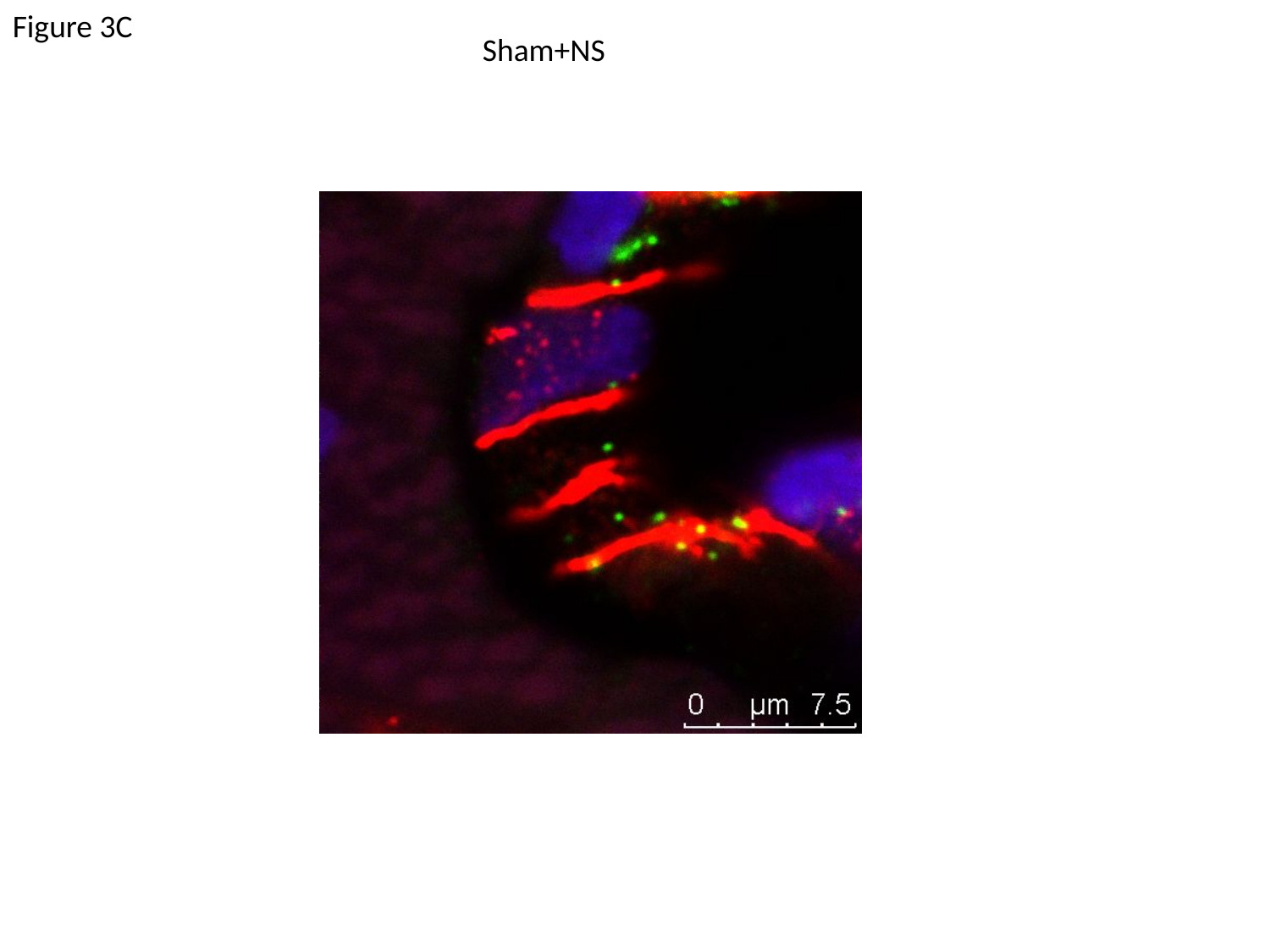

Figure 3C
Sham+NS

## Slide 15
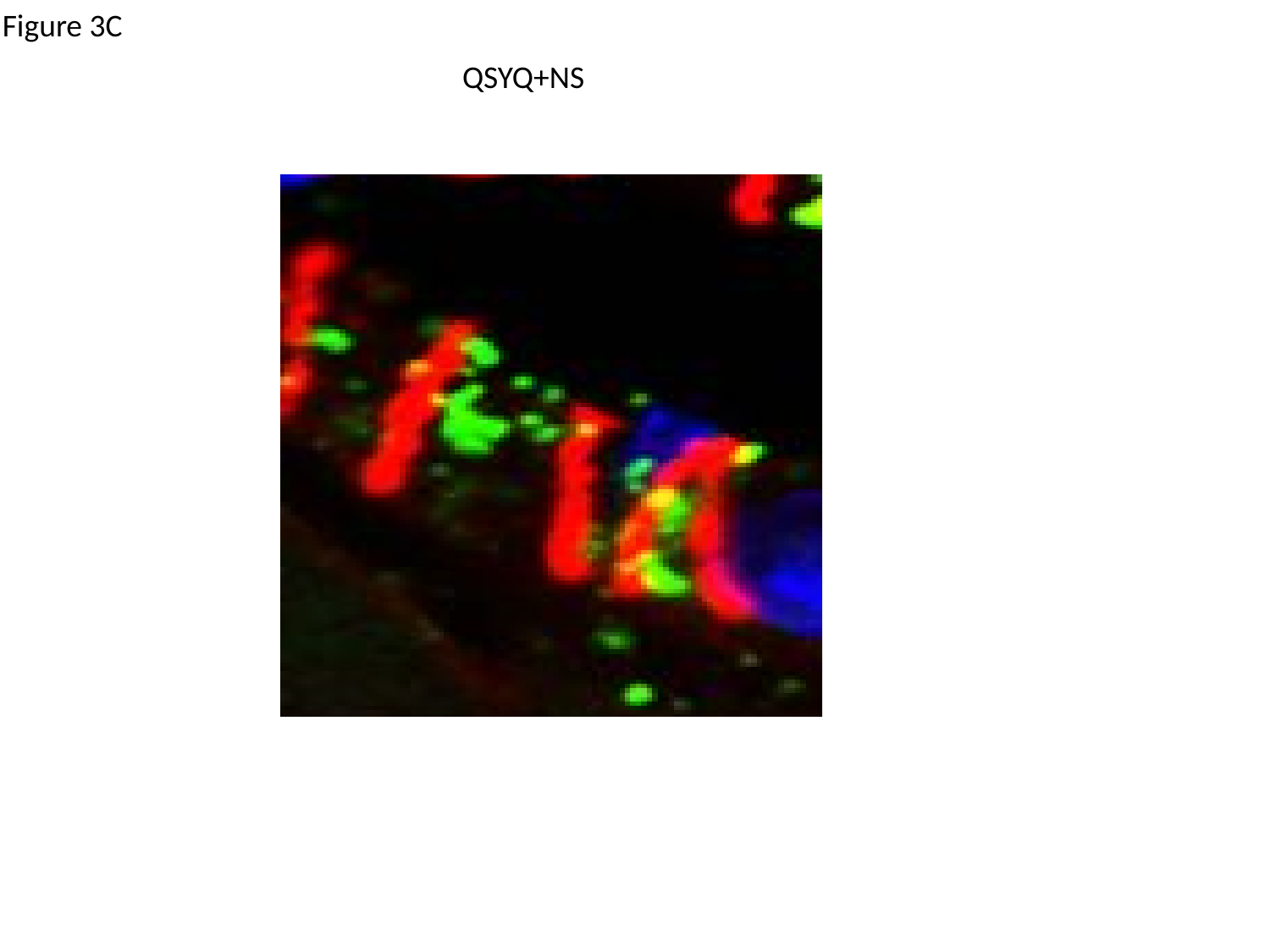

Figure 3C
QSYQ+NS

## Slide 16
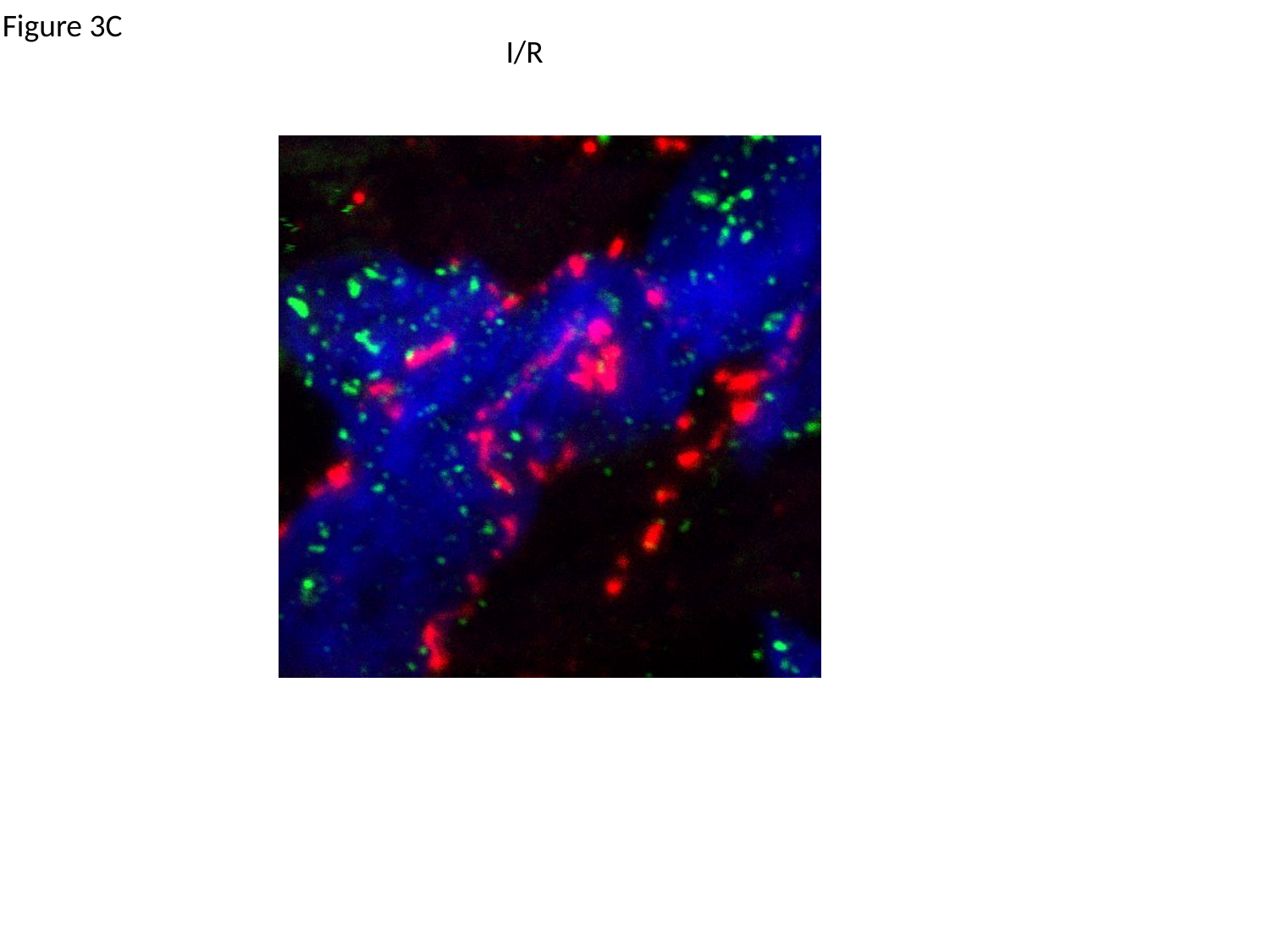

Figure 3C
I/R

## Slide 17
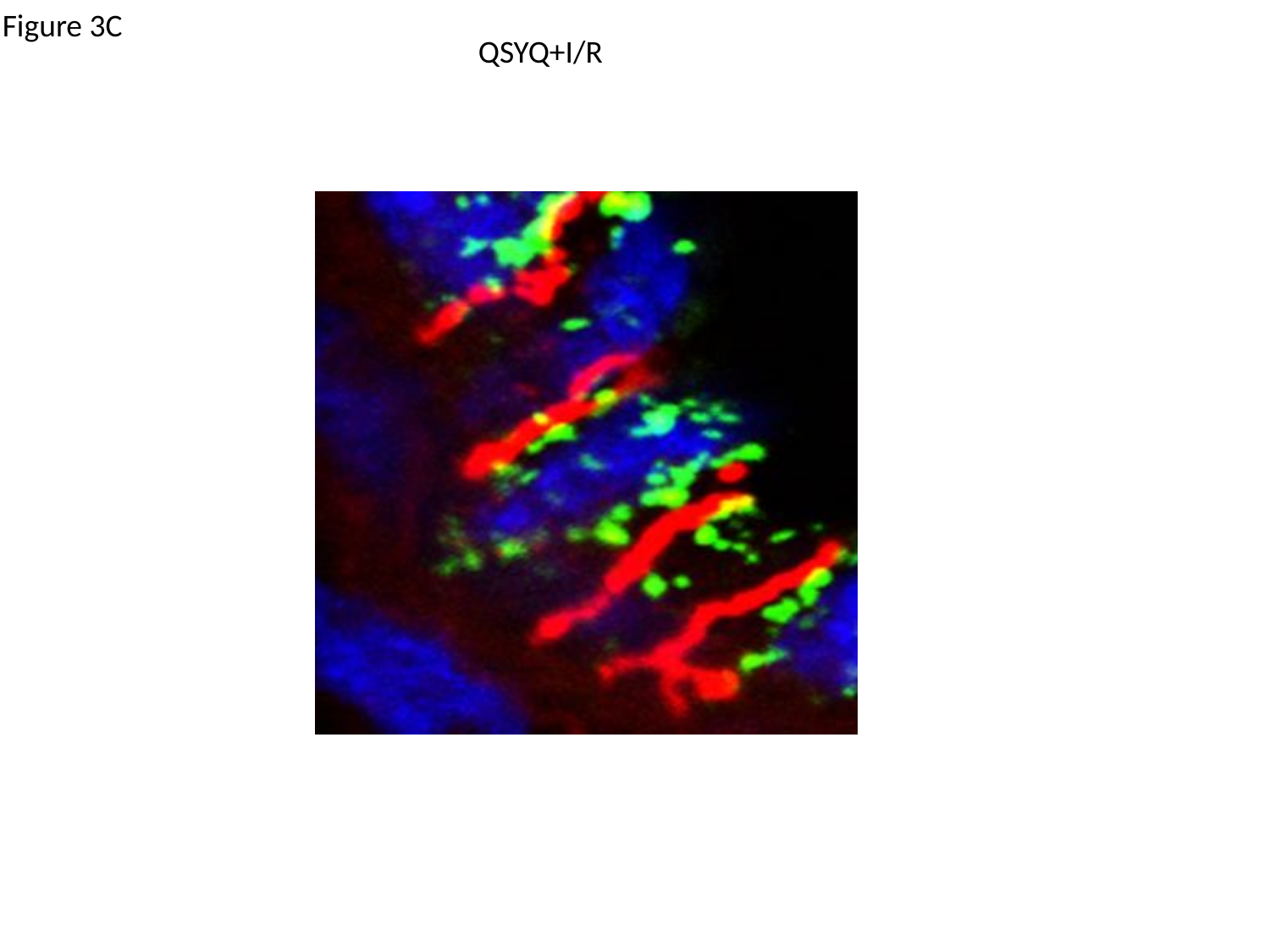

Figure 3C
QSYQ+I/R

## Slide 18
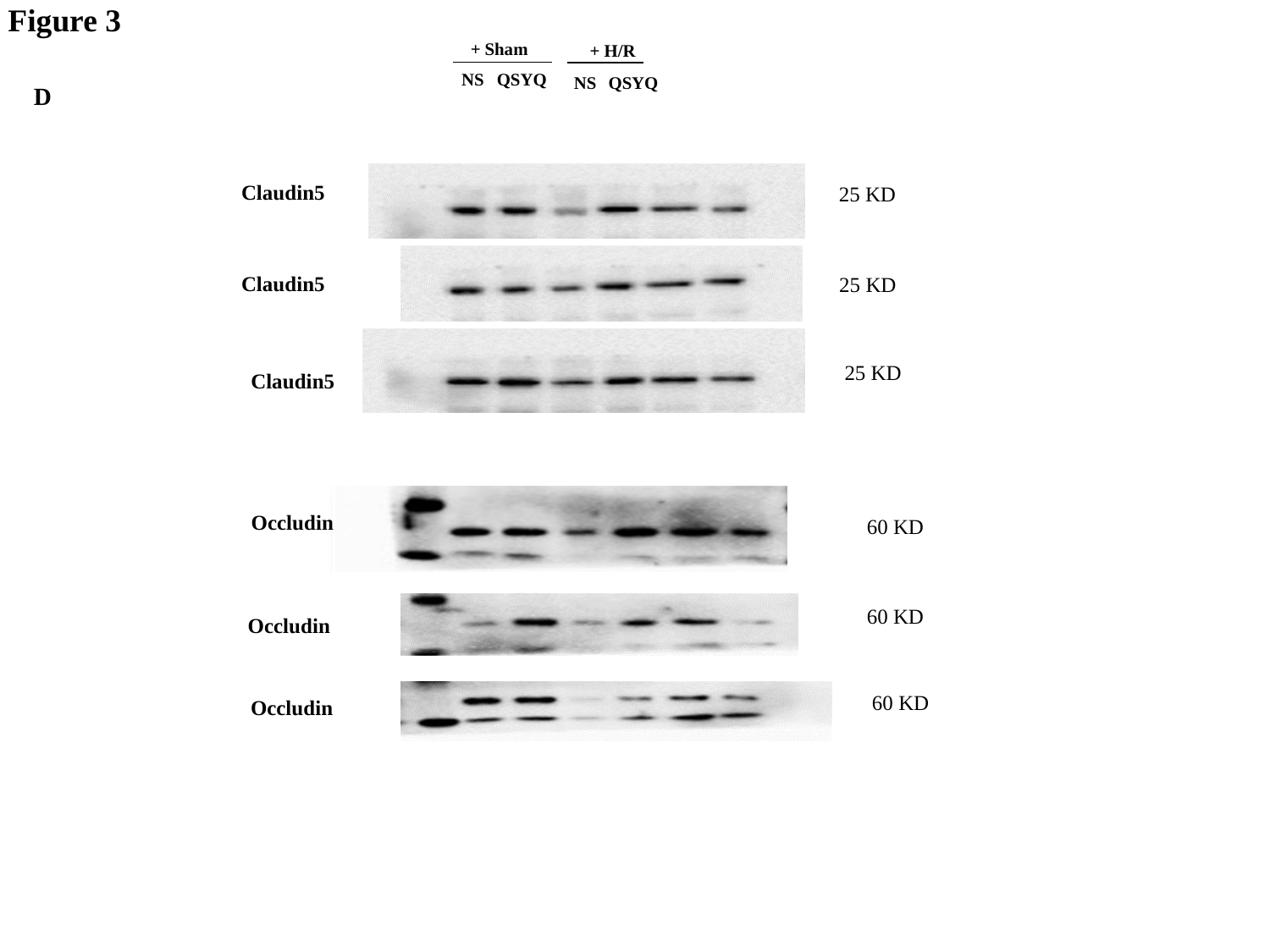

Figure 3
+ Sham
+ H/R
NS
QSYQ
QSYQ
NS
D
Claudin5
25 KD
Claudin5
25 KD
25 KD
Claudin5
Occludin
60 KD
60 KD
Occludin
60 KD
Occludin

## Slide 19
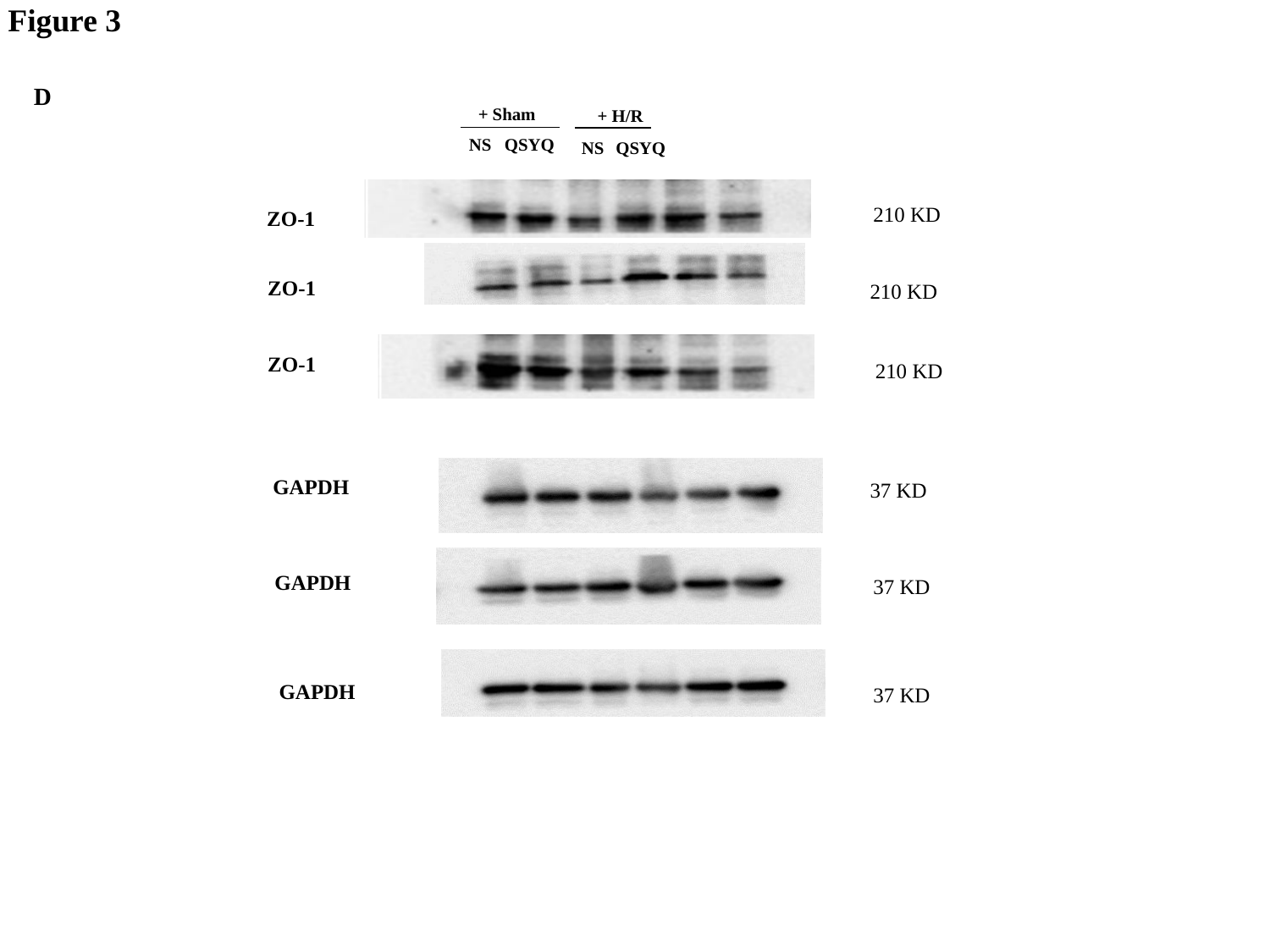

Figure 3
D
+ Sham
+ H/R
NS
QSYQ
QSYQ
NS
210 KD
ZO-1
ZO-1
210 KD
ZO-1
210 KD
GAPDH
37 KD
GAPDH
37 KD
GAPDH
37 KD

## Slide 20
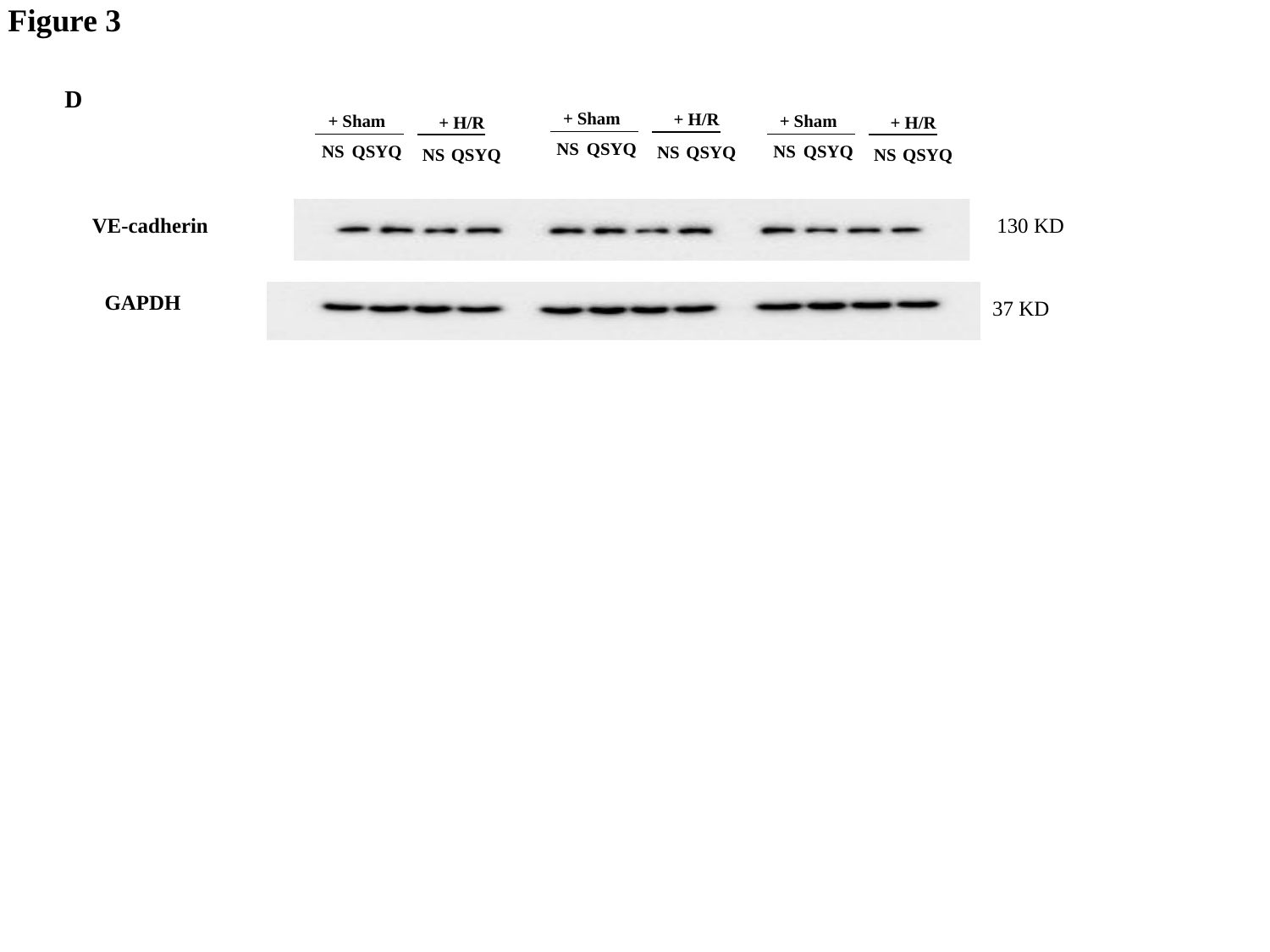

Figure 3
D
+ Sham
+ H/R
NS
QSYQ
QSYQ
NS
+ Sham
+ H/R
NS
QSYQ
QSYQ
NS
+ Sham
+ H/R
NS
QSYQ
QSYQ
NS
130 KD
VE-cadherin
GAPDH
37 KD

## Slide 21
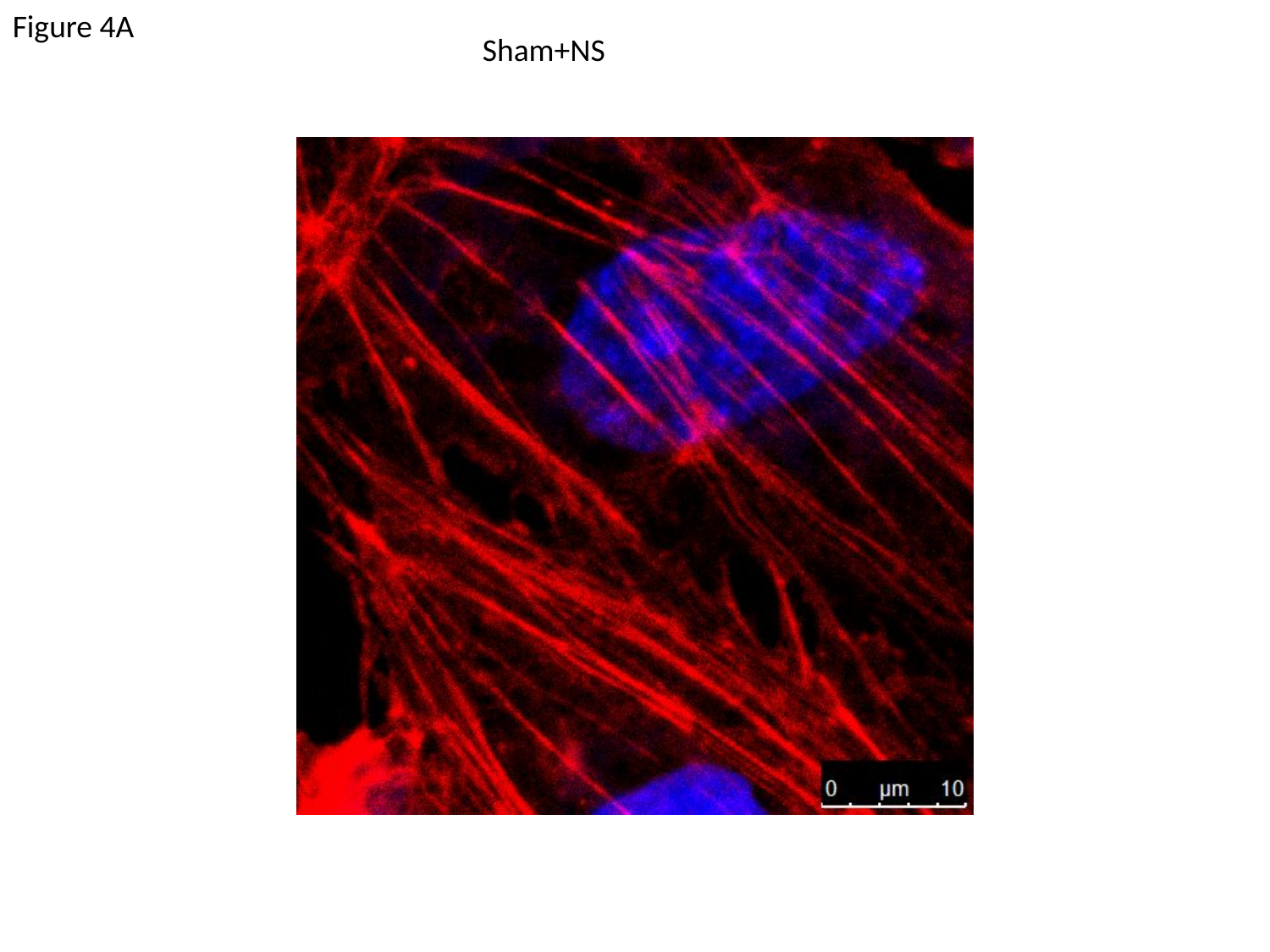

Figure 4A
Sham+NS

## Slide 22
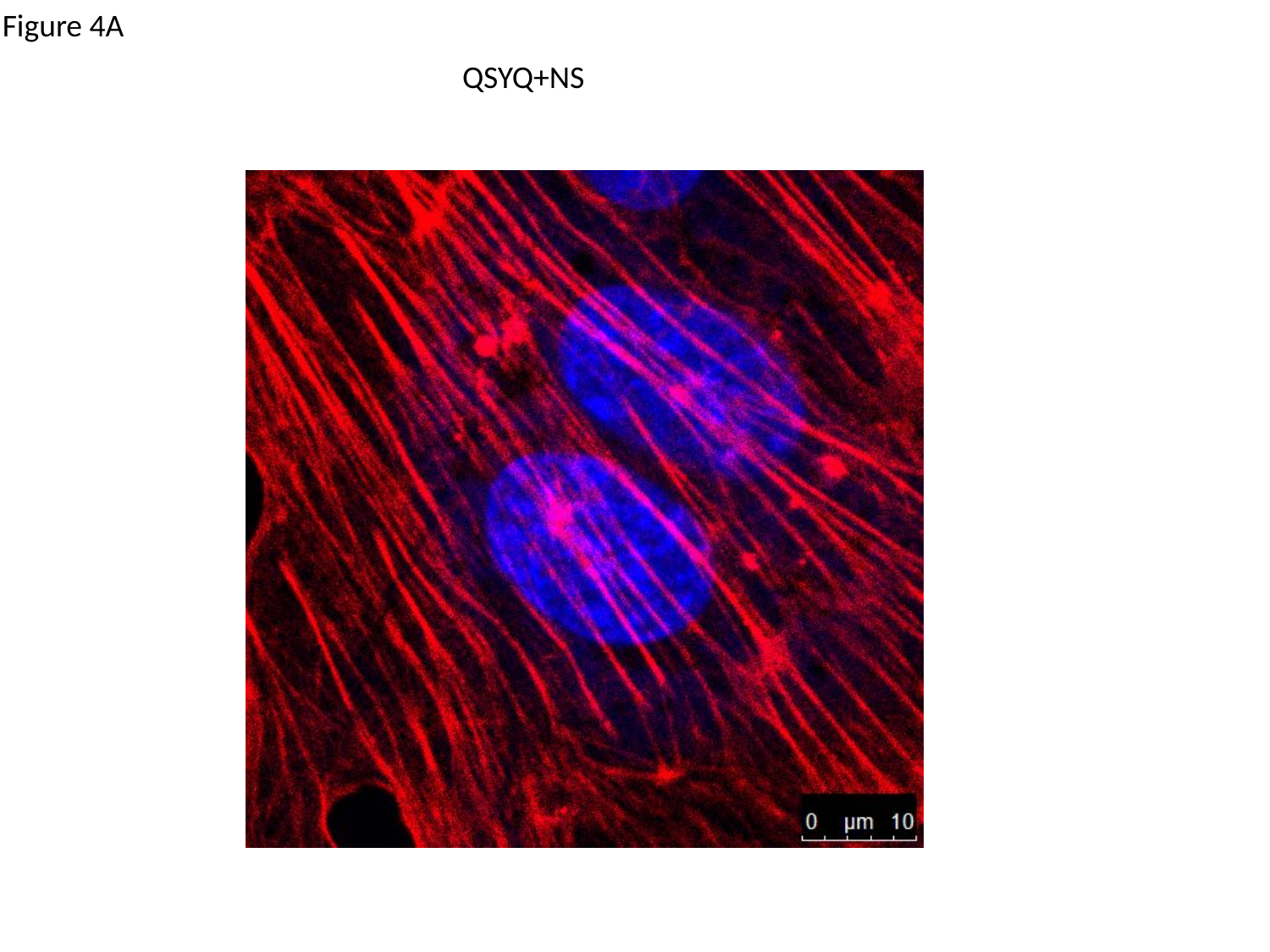

Figure 4A
QSYQ+NS

## Slide 23
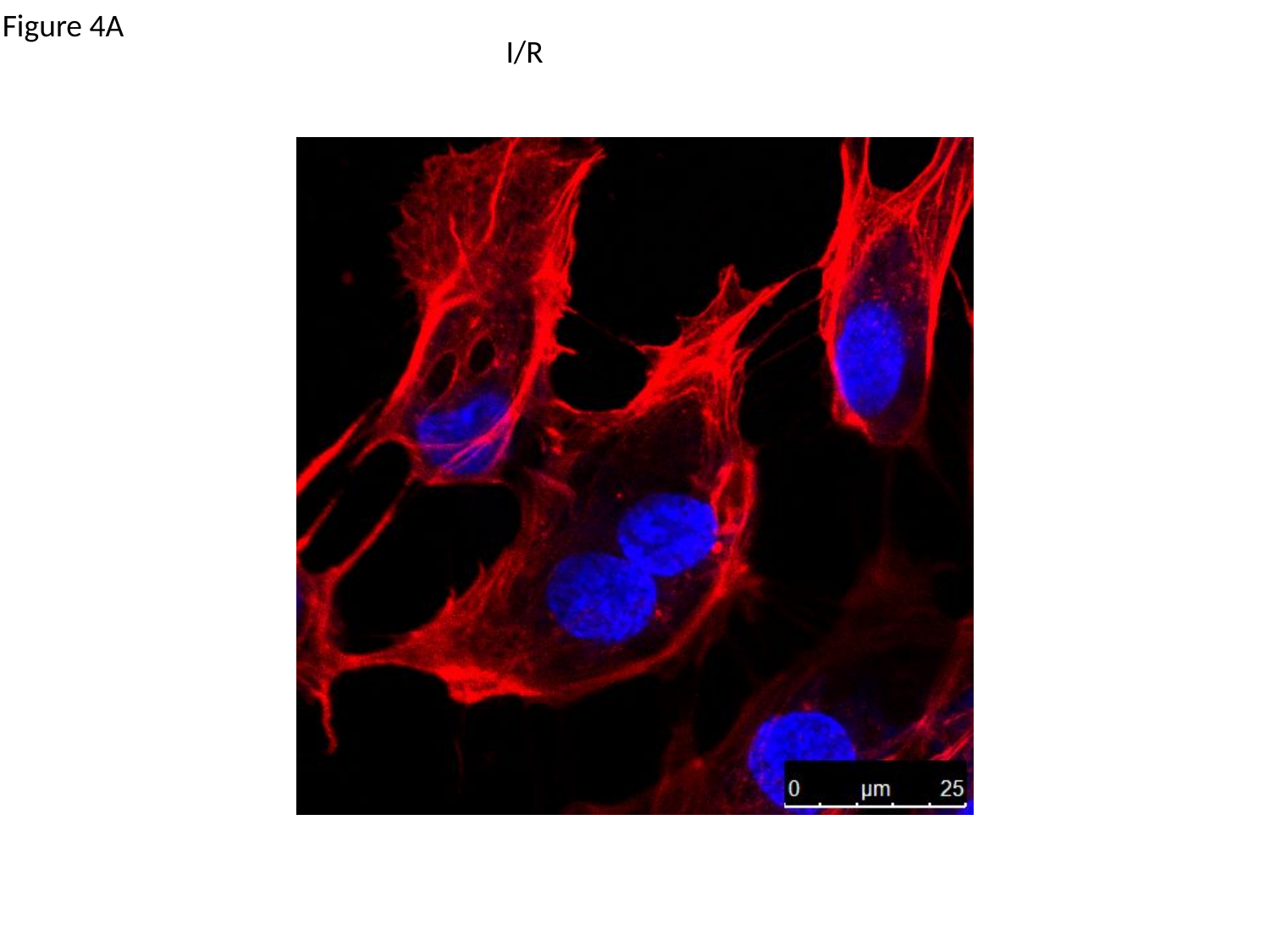

Figure 4A
I/R

## Slide 24
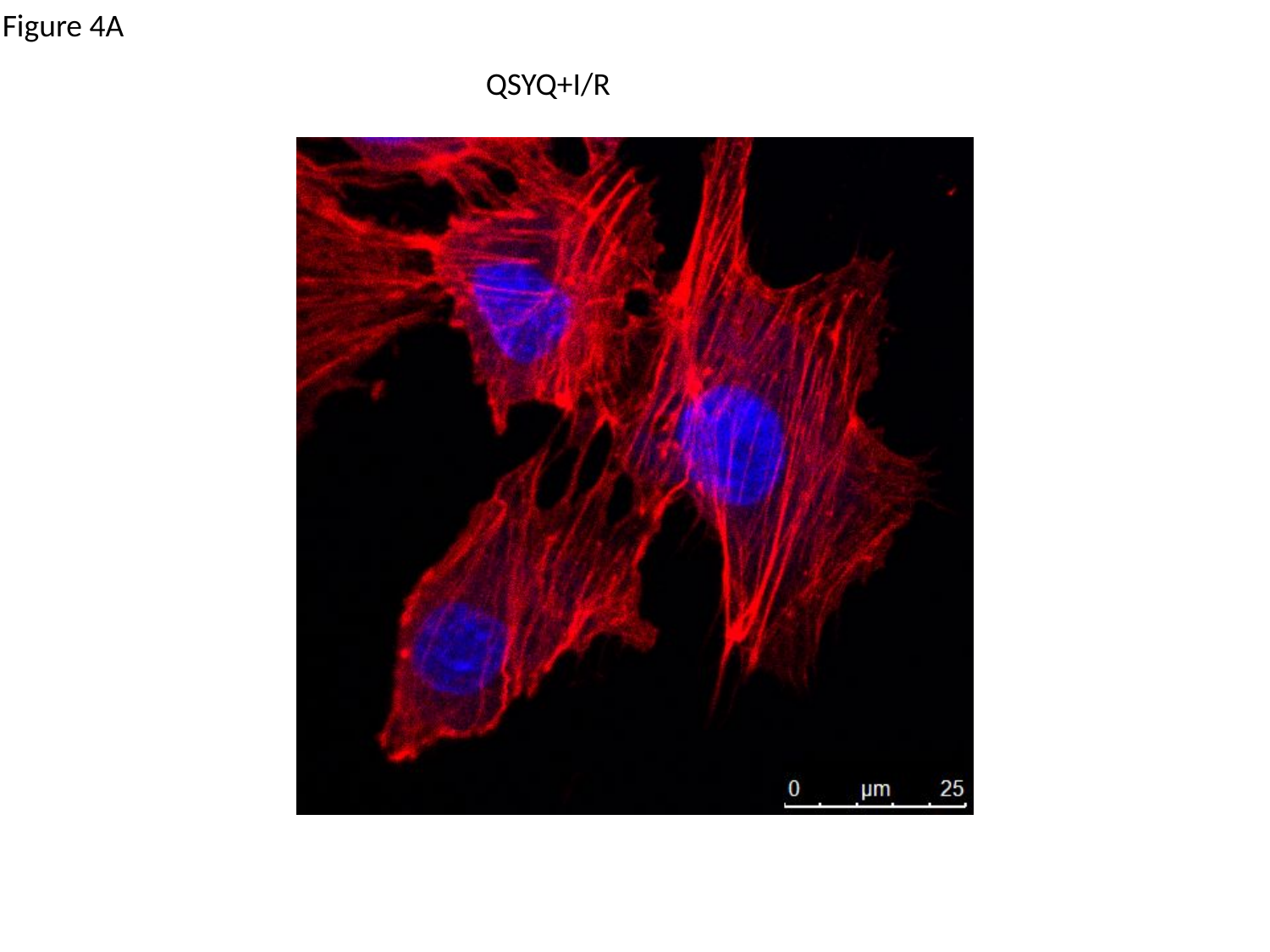

Figure 4A
QSYQ+I/R

## Slide 25
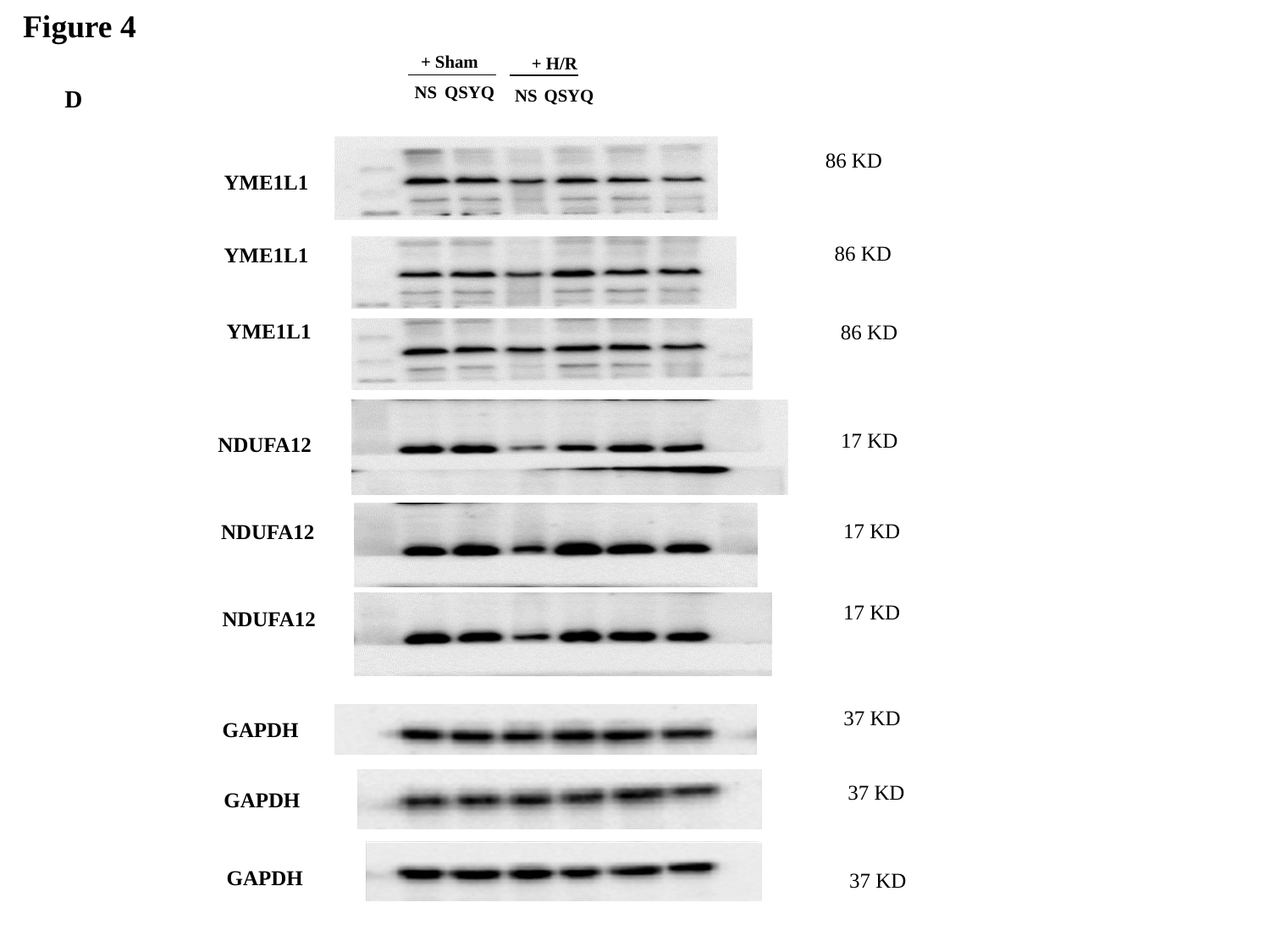

Figure 4
+ Sham
+ H/R
NS
QSYQ
QSYQ
NS
D
86 KD
YME1L1
86 KD
YME1L1
YME1L1
86 KD
17 KD
NDUFA12
17 KD
NDUFA12
17 KD
NDUFA12
37 KD
GAPDH
37 KD
GAPDH
GAPDH
37 KD

## Slide 26
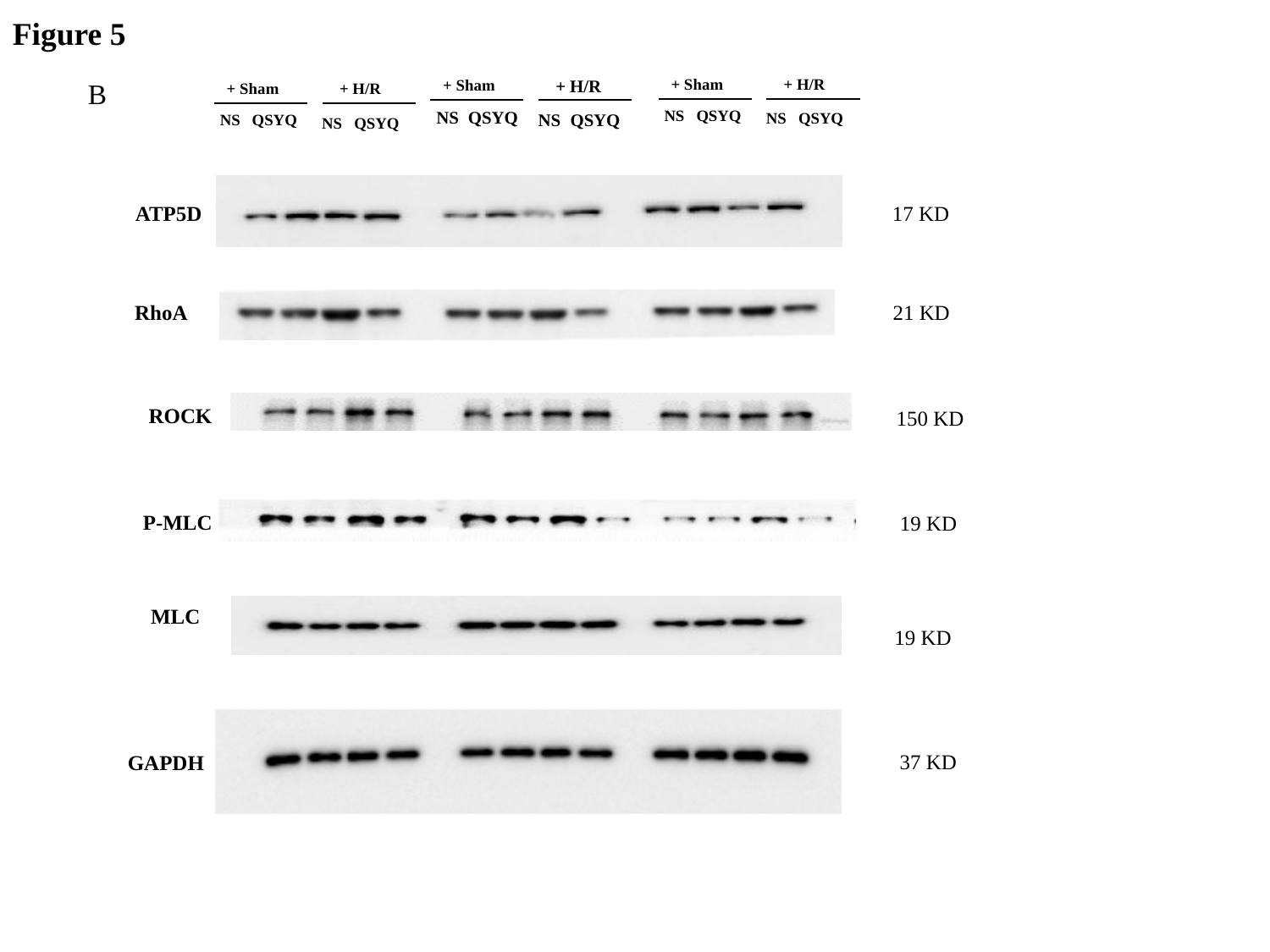

Figure 5
+ Sham
+ H/R
NS
QSYQ
QSYQ
NS
+ Sham
+ H/R
NS
QSYQ
QSYQ
NS
B
+ Sham
+ H/R
NS
QSYQ
QSYQ
NS
ATP5D
17 KD
21 KD
RhoA
ROCK
150 KD
P-MLC
19 KD
MLC
19 KD
37 KD
GAPDH

## Slide 27
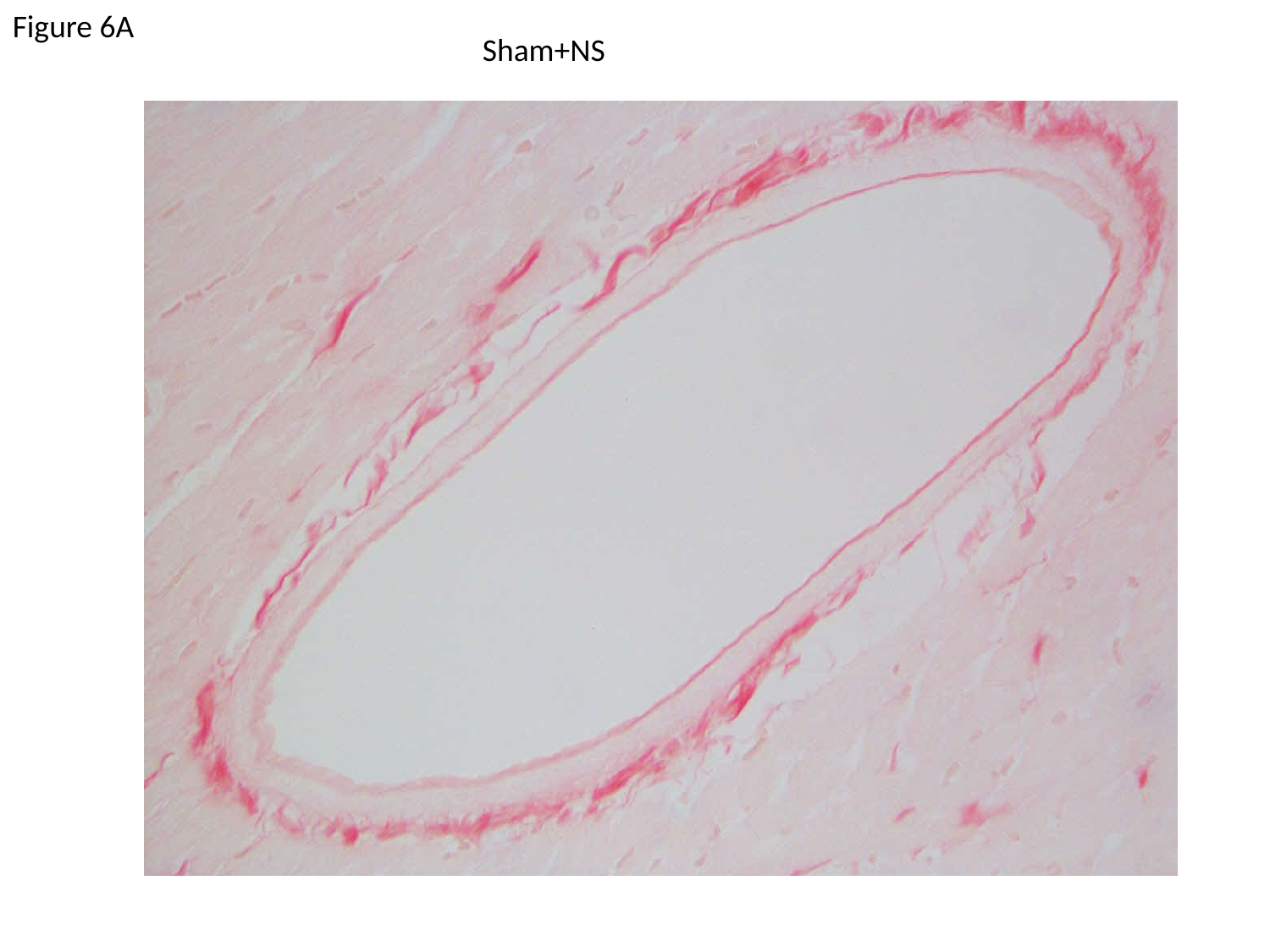

Figure 6A
Sham+NS

## Slide 28
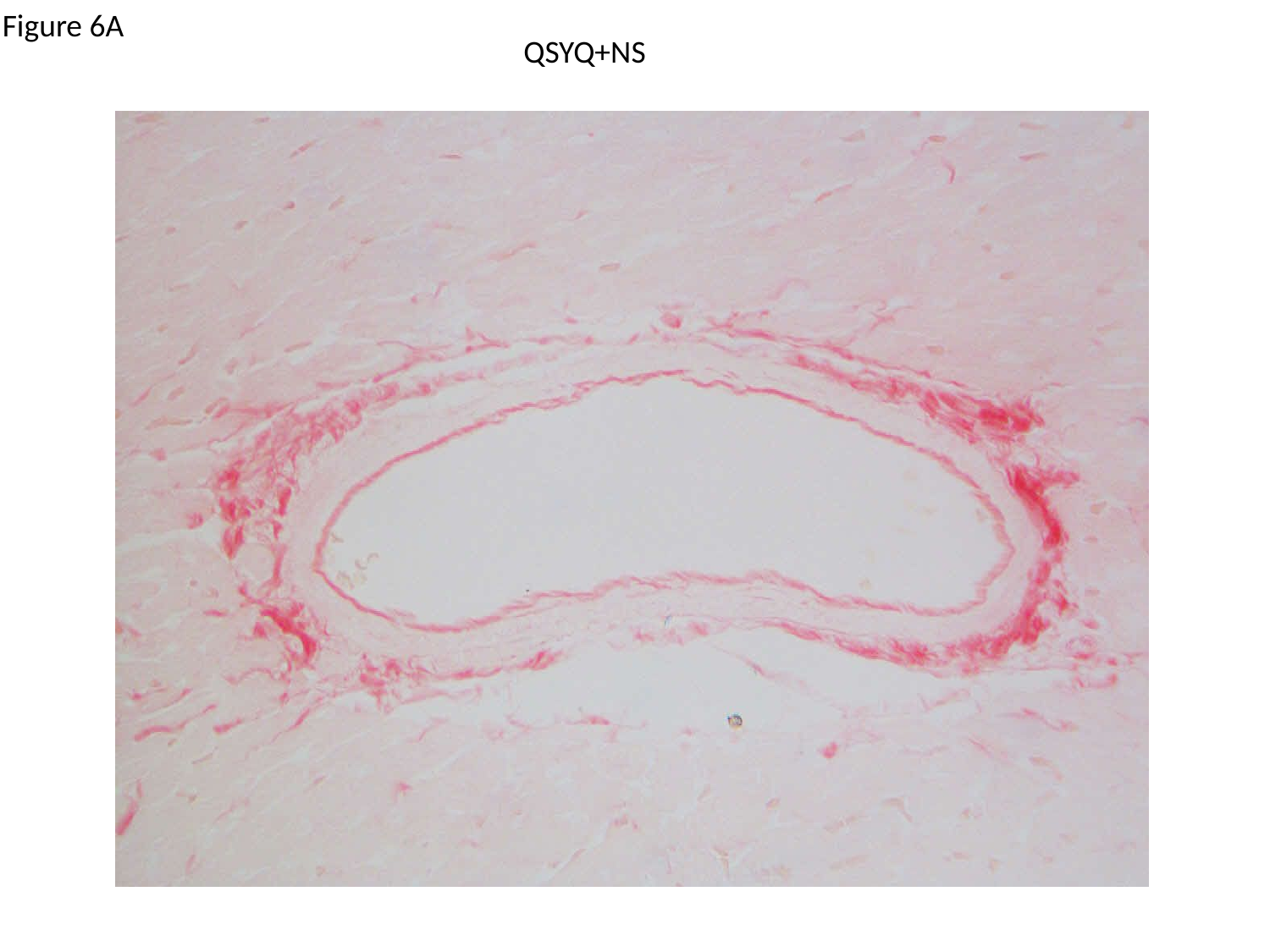

Figure 6A
QSYQ+NS

## Slide 29
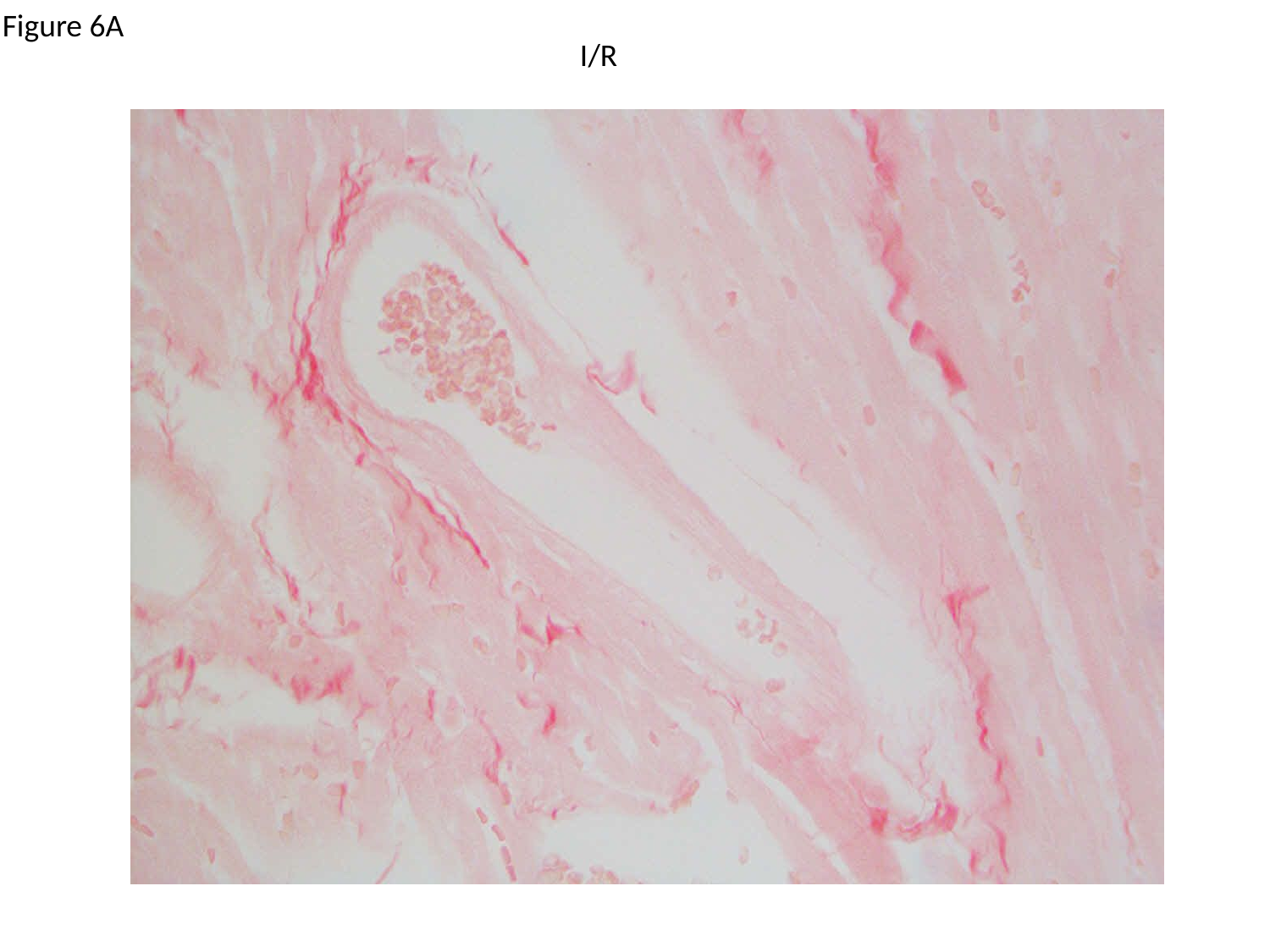

Figure 6A
I/R

## Slide 30
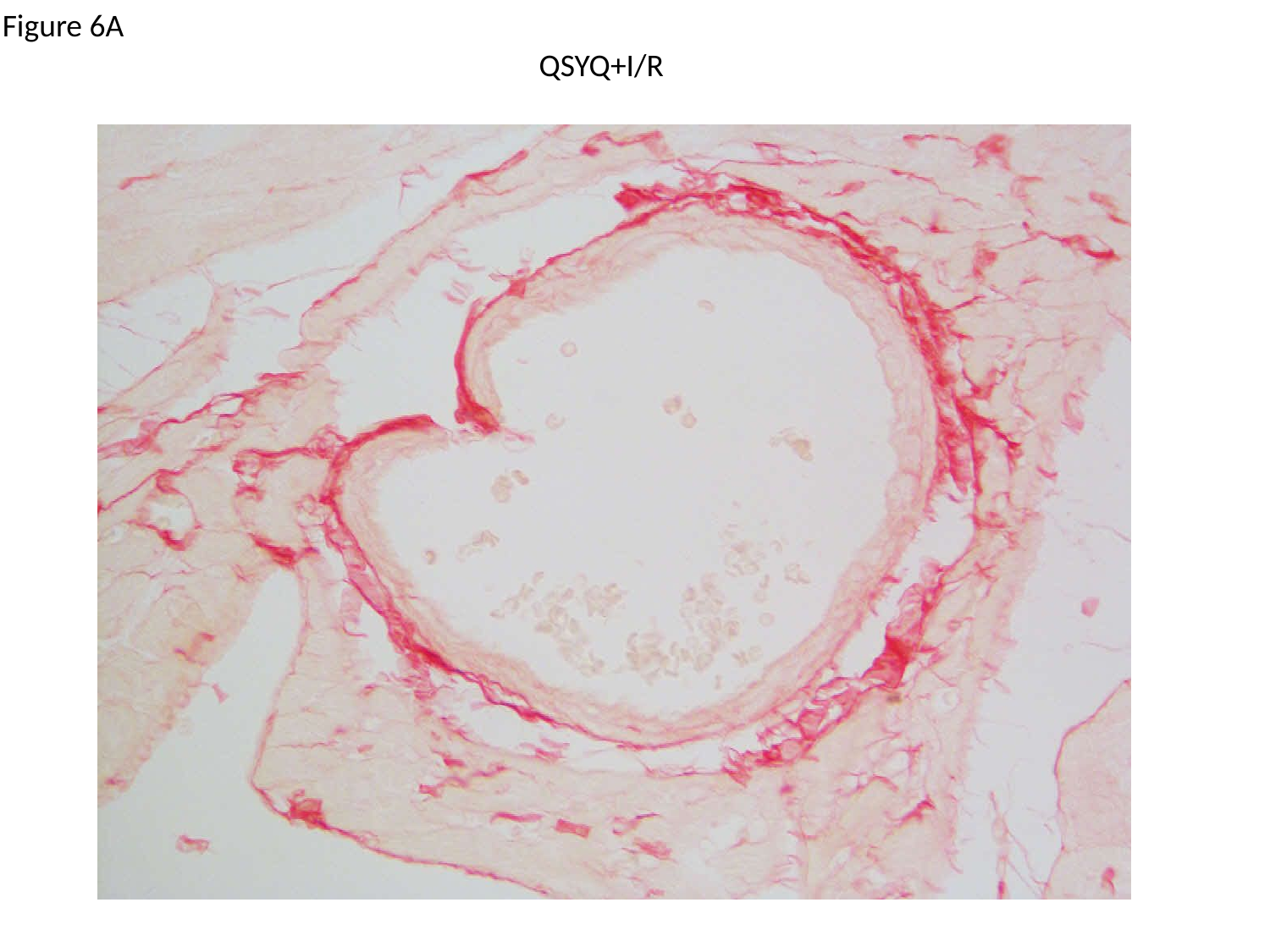

Figure 6A
QSYQ+I/R

## Slide 31
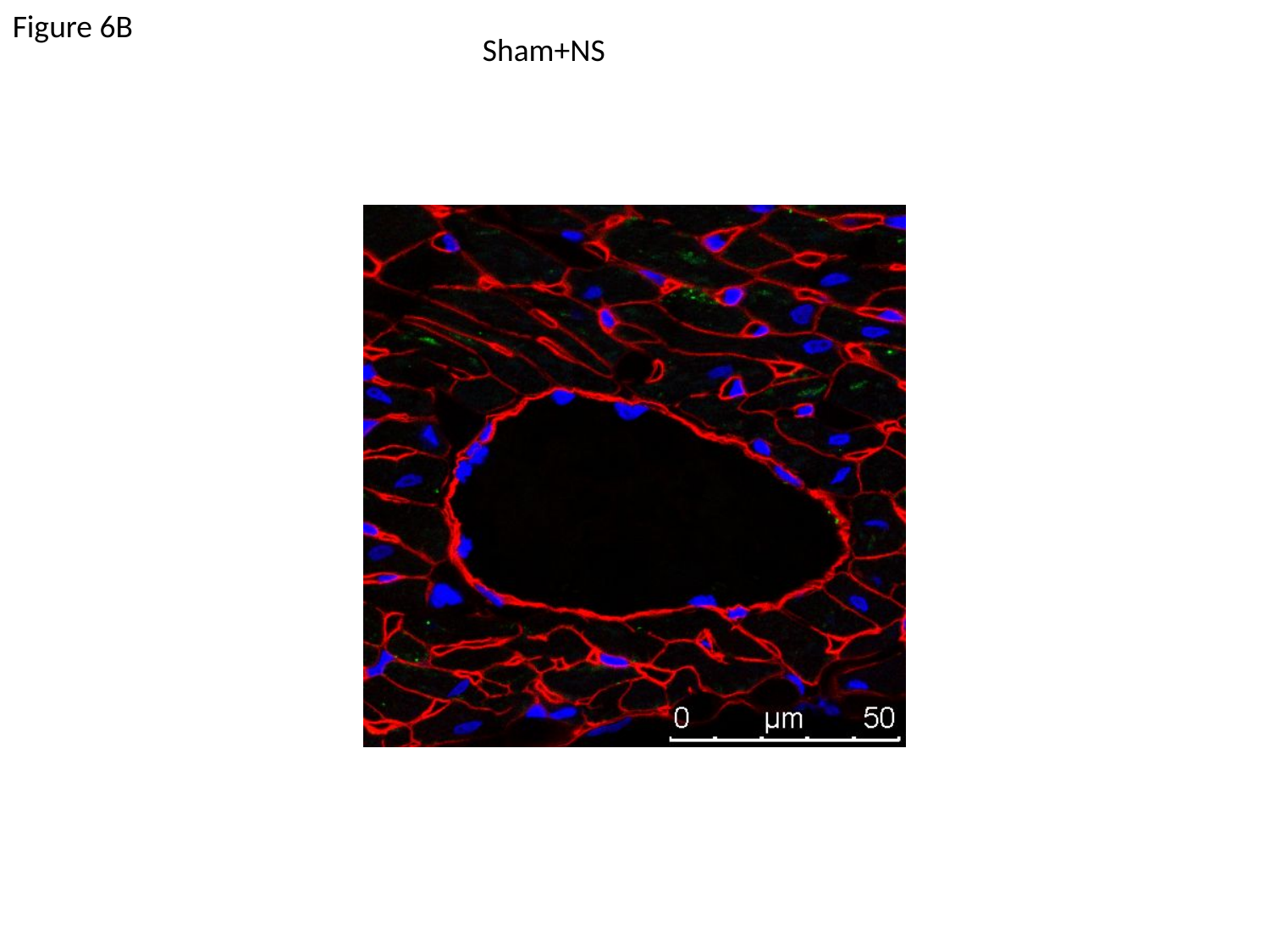

Figure 6B
Sham+NS

## Slide 32
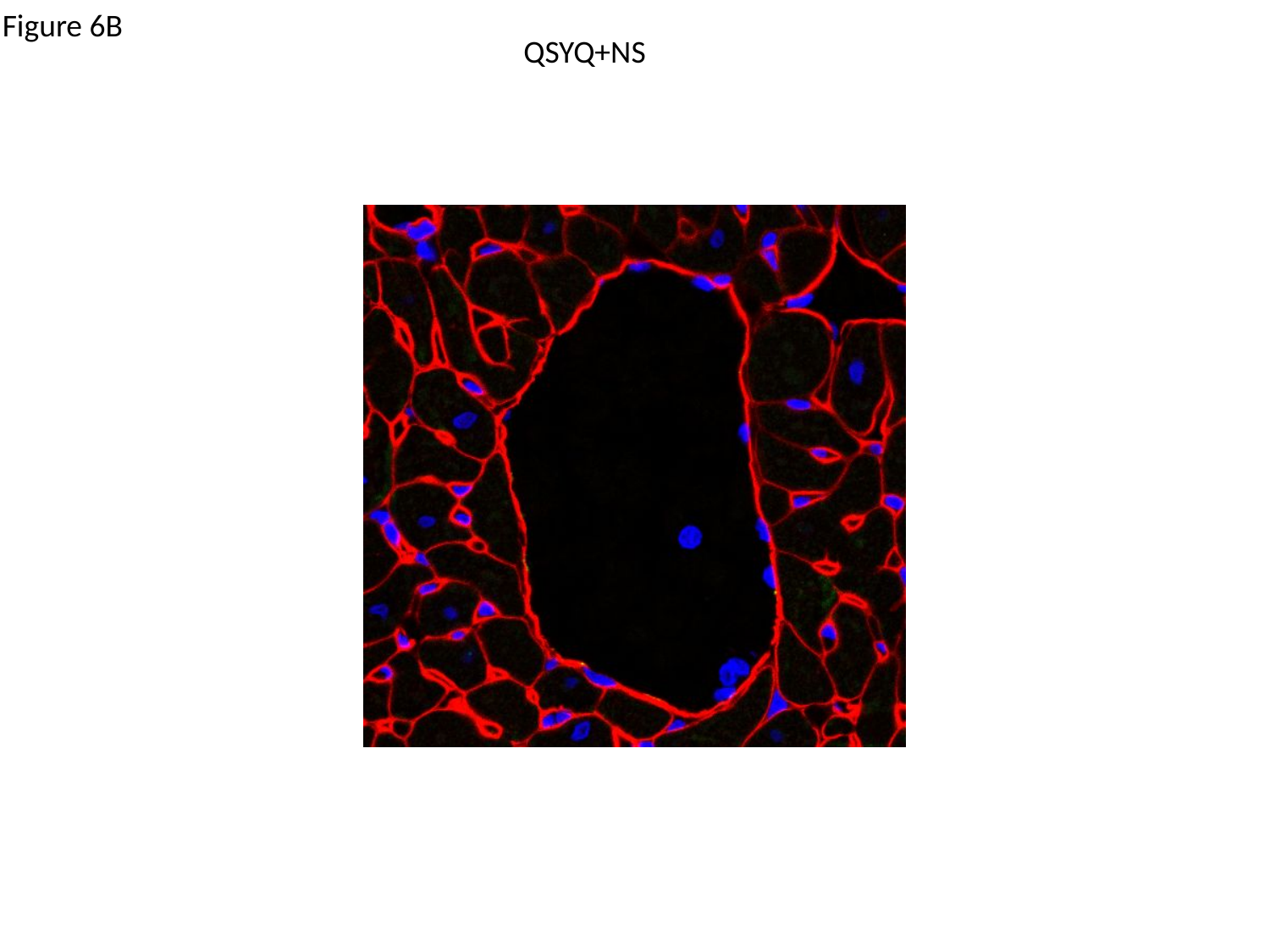

Figure 6B
QSYQ+NS

## Slide 33
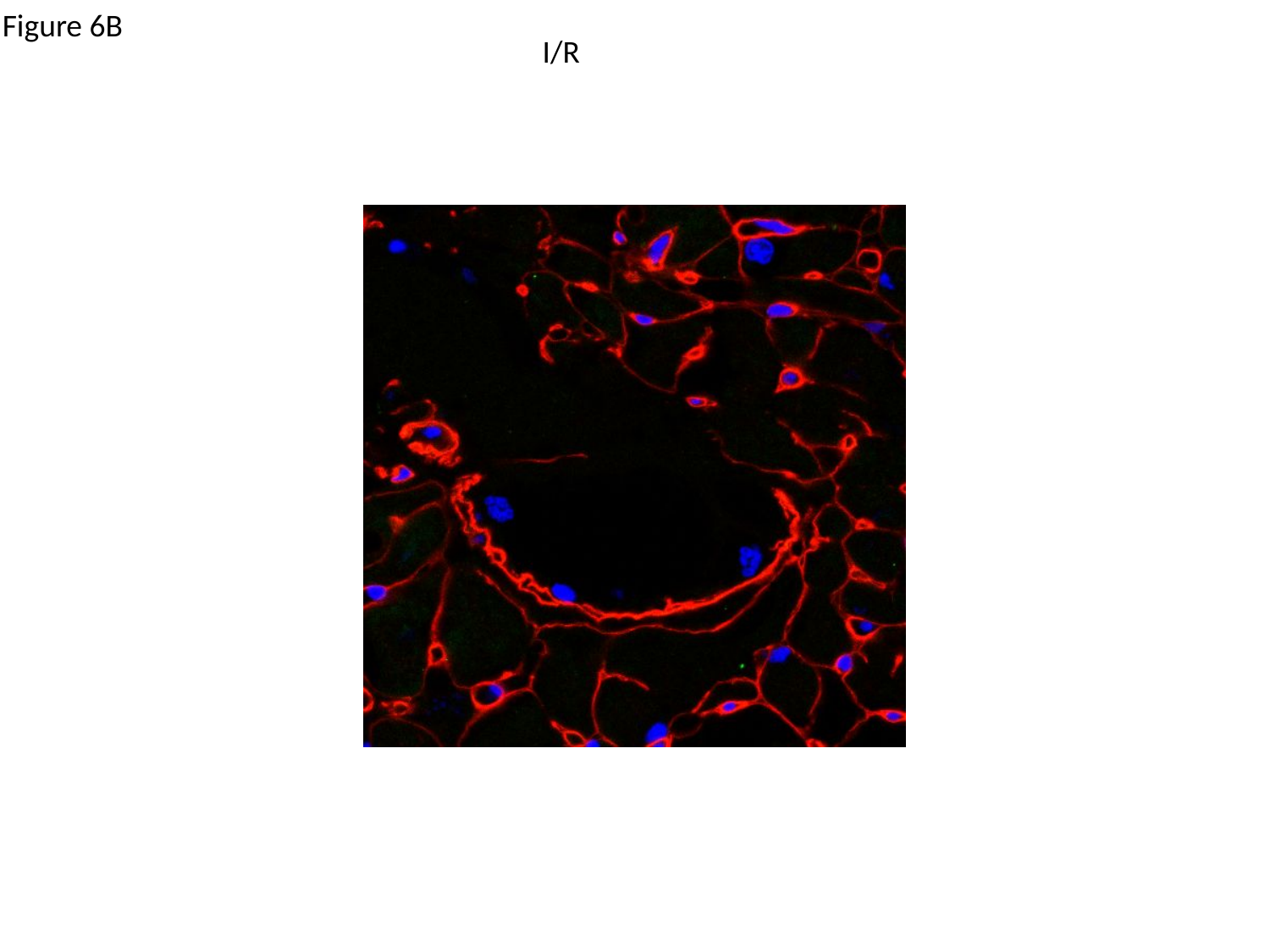

Figure 6B
I/R

## Slide 34
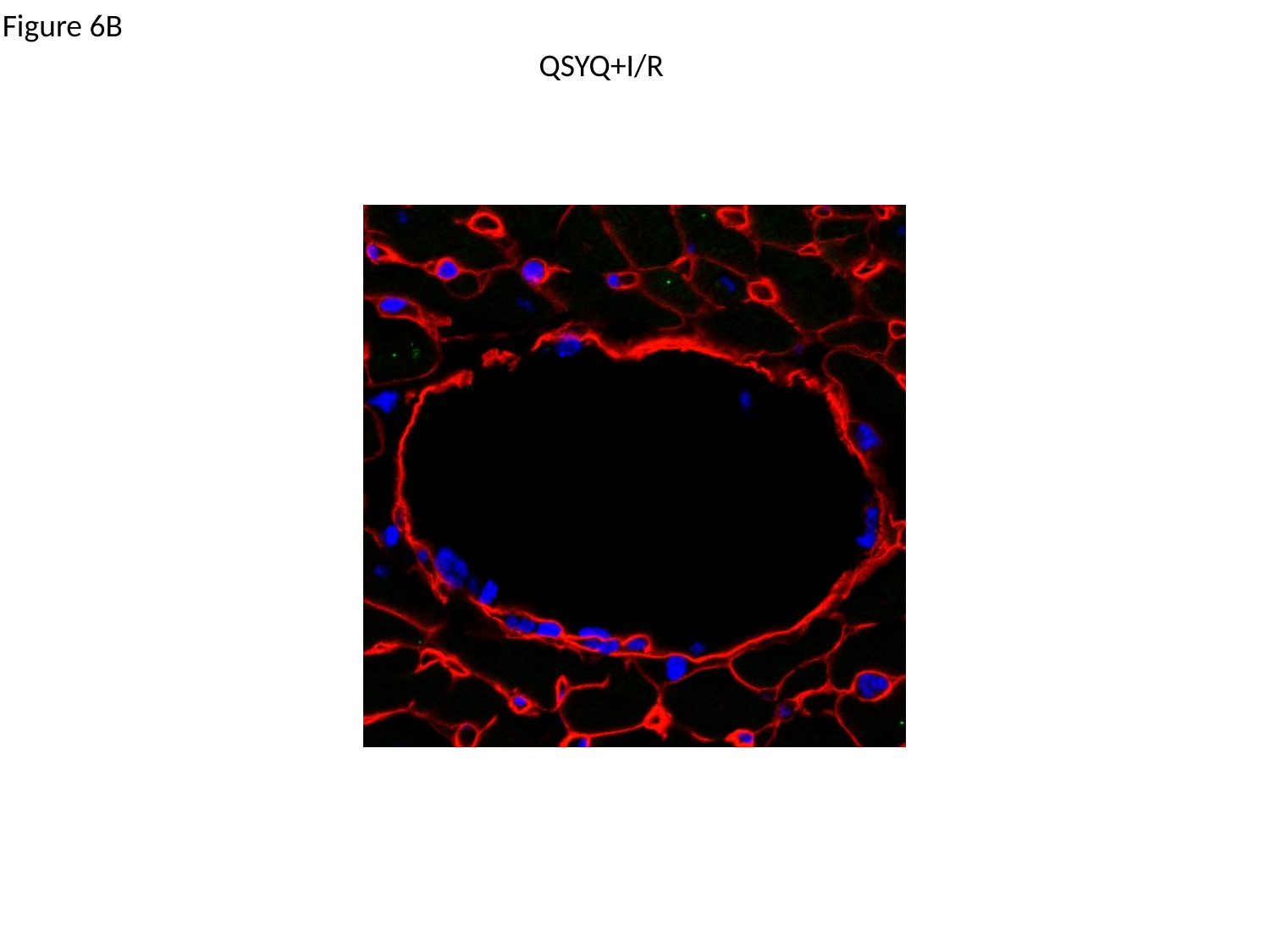

Figure 6B
QSYQ+I/R

## Slide 35
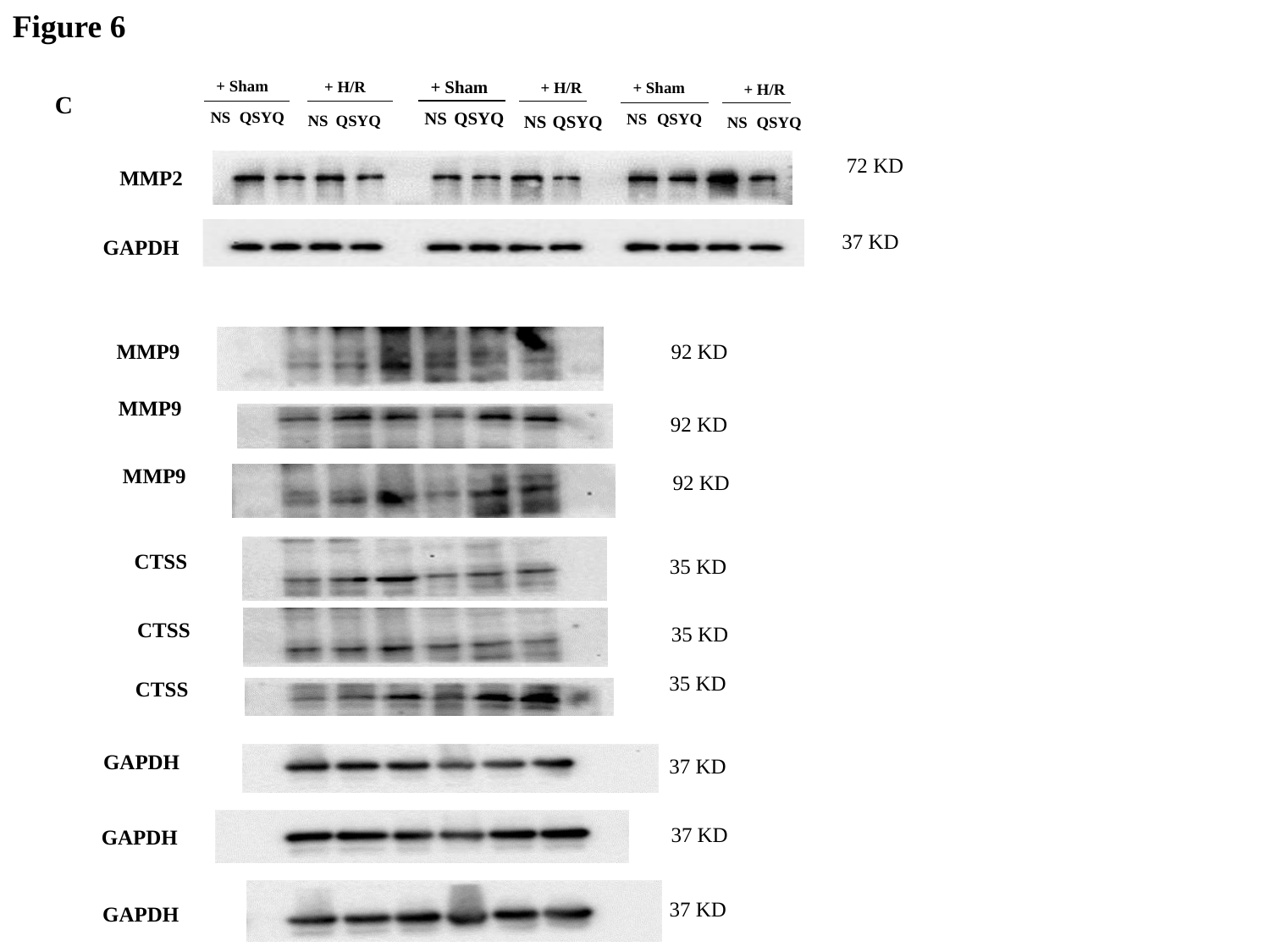

Figure 6
+ Sham
+ H/R
NS
QSYQ
QSYQ
NS
+ Sham
+ H/R
NS
QSYQ
QSYQ
NS
+ Sham
+ H/R
NS
QSYQ
QSYQ
NS
C
72 KD
MMP2
37 KD
GAPDH
MMP9
92 KD
MMP9
92 KD
MMP9
92 KD
CTSS
35 KD
CTSS
35 KD
35 KD
CTSS
GAPDH
37 KD
37 KD
GAPDH
37 KD
GAPDH

## Slide 36
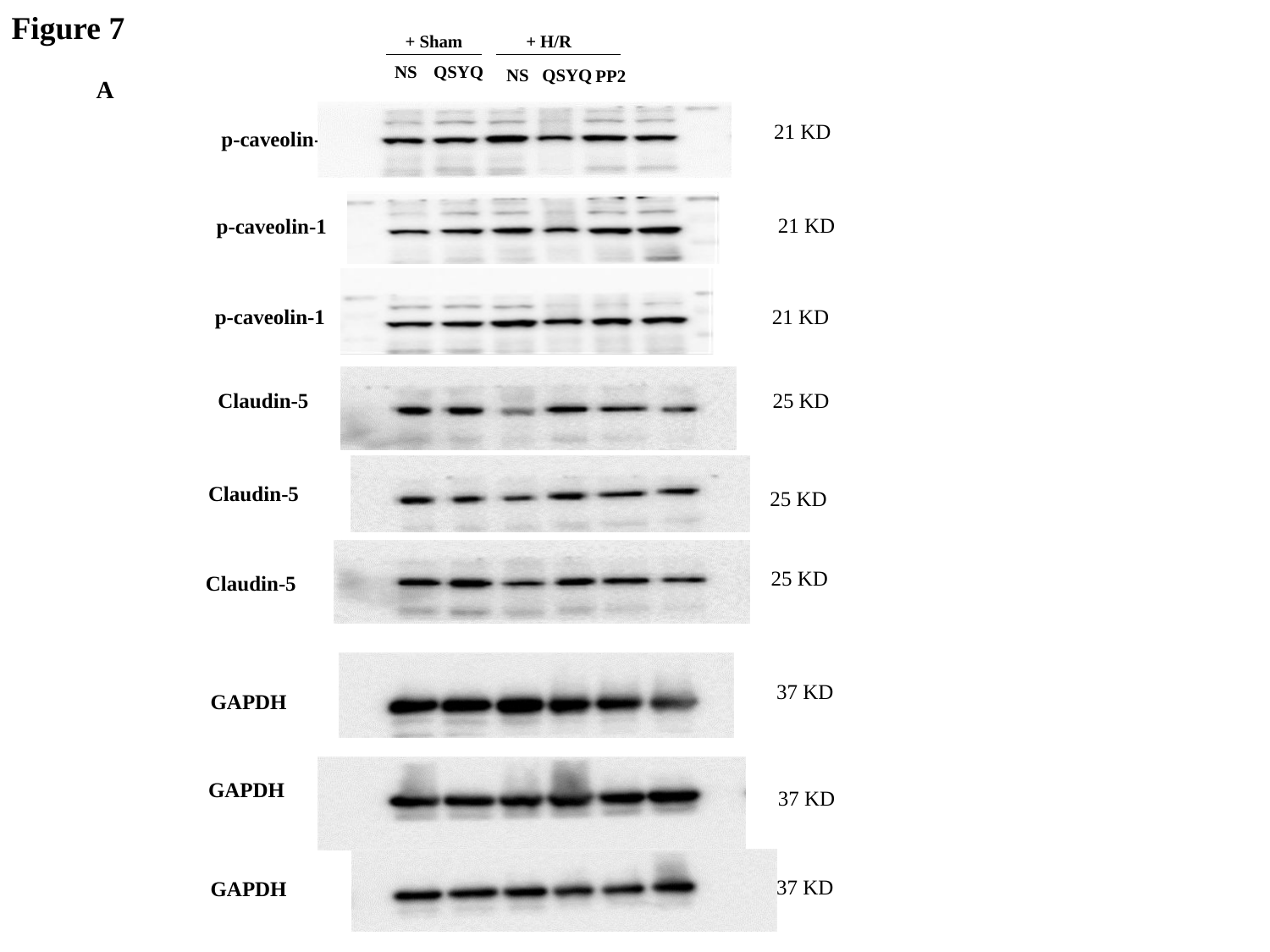

Figure 7
+ Sham
+ H/R
NS
QSYQ
QSYQ
NS
PP2
A
21 KD
p-caveolin-1
21 KD
p-caveolin-1
21 KD
p-caveolin-1
25 KD
Claudin-5
Claudin-5
25 KD
25 KD
Claudin-5
37 KD
GAPDH
GAPDH
37 KD
37 KD
GAPDH
